# How communitization begets and endures *sarkarikaran*: a witnessed history of community action for health in India’s national rural health mission

**DOI:** 10.1186/s12913-025-13058-0

**Published:** 2025-07-08

**Authors:** Devaki Nambiar, Neymat Chadha

**Affiliations:** 1 George Institute for Global Health, 308 Elegance Tower, Jasola District Centre, New Delhi, 110025 India; 2 George Institute for Global Health, University of New South Wales, Sydney, Australia; 3 Prasanna School of Public Health, Manipal Academy of Higher Education, Manipal, India

**Keywords:** Community action for health (CAH), India, National health mission (NHM), Community participation, Health policy and systems research (HPSR), Oral history, Witness seminar

## Abstract

The legacy of Community Action for Health (CAH) in India traces back to the global momentum for primary health care galvanized by the Alma Ata Declaration and post–World War II social movements. In 2024, the World Health Assembly endorsed a resolution on institutionalising CAH and other forms of social participation as a core pillar of health reform. The Indian experience of institutionalising CAH under its erstwhile National Rural Health Mission offers an example of sustained national-scale implementations of CAH globally, yet its lessons—both successes and blind spots—remain under-analysed. This paper aims to fill that gap by critically examining the trajectory of CAH since 2005 and exploring its implications for the operationalization of the SPH Resolution. In 2021, two virtual Witness Seminars and four in-depth interviews were conducted with leaders of Civil Society Organizations (CSOs), program implementers, and policy decision-makers involved in the institutionalization of Community Action for Health (CAH) under the National Rural Health Mission (NRHM). Participants included. Seminars and interviews explored key events, actors, processes, and contextual factors that shaped the evolution of CAH. All sessions were fully transcribed and analysed using ATLAS.ti (version 22). Our analysis of the evolution of CAH in India suggests four phases – leading to and flowing away from governmentality. While initially communitization involved collaborative dialogue, debate and system redesign, pilots of CAH rolled out across Indian states with varying strategies, ownership, and stakeholdership. Roles of community began to shift into that of agents and providers as part of “sarkarikaran” or governmentalization of community processes. A final phase suggests that like flowing water, CAH may continue to reconfigure state-society relations in the wake of emerging challenges, less formalised, more embedded interactions. This suggests that institutionalising, standardizing and centralising CAH is neither lasting, nor desirable.

**Supplementary Information:**

The online version contains supplementary material available at 10.1186/s12913-025-13058-0.

## Highlights

Community action for health has a vaunted legacy and remains highly relevant across contexts today.

Witness seminar methodology can help understand how community action for health models have evolved in a decentralized social democratic context like India’s.

Analysis reveals four (nautical) phases in the evolution of community action for health in India: building the ship, setting sail, floundering waters, and flowing where there’s space (or even when there’s not).

In the current context, where institutionalization of social participation for health is being called for, understanding these histories and trajectories offers many insights.

**Supplementary Information:**

The online version contains supplementary material available at 10.1186/s12913-025-13058-0.

## Introduction

Key milestones in global health, such as the Alma-Ata Declaration (1978), the formation of the People’s Health Movement (2000), and the International Conference on Population and Development (1994), have inspired calls for democratization of decision-making, calling for citizens to be given voice to make governments more responsive, cost-effective and accountable [1, 2]. These were in turn part of the legacy of the global Non-Aligned Movement, the New International Economic Order and moves to create alternative global development paradigms [3, 4]. Since the 1990 s, the association between increased community mobilization and improved accountability mechanisms across health systems governance has become progressively clearer [5–9]. In 2024, a watershed resolution was passed at the World Health Assembly, calling for institutionalisation of ‘social participation for health, ’ a process by which “social actors group their collective potential to achieve a collective good,” such as health reform [10, 11]. As countries seek to operationalise and domesticate this global resolution, it bears recognition that social participation or community action for health do not operate on a *tabula rasa*, rather there are important histories and legacies to acknowledge and more deeply understand so we may build on or surmount them [12].

Social participation and community action have a number of conceptual cousins including community participation, citizen engagement, social accountability and are associated with a range of terms like participatory governance, collaborative governance and deliberative democracy [10]. Normative theorization around these concepts find their legacy across cultures in the world, but were more formally developed as part of the global development literature of post World War period [13, 14]. This crescendoed in the 1990 s as across the world, the legal environment began to mandate citizen participation in reform in many, particularly de-colonizing countries of the world [15]. Some disenchantment with social participation and citizen engagement in governance was seen at the turn of the millennium [16, 17], with more recent developments or what is also called “the deliberative wave” in planning [18, 19], climate/environmentalist [20–22], public service management [23] and public health [12, 24–27] discourses. While much of the theorization focuses on typologies of participation and power [28–31], or on modes or levels of engagement [27, 32], others speak to features of these efforts as well as their failure and shortcomings at scaled or over time [16, 17, 30, 33, 34]. In terms of implications for governance, Fox and colleagues draw on examples across the world, for example to theorize that “openings from above” (invited spaces) or “pressure from below” (invented spaces) with triggers, openings, action, roadblocks, and finally power shifts in both state and society over time [35]. Much of the literature is synthesis or snapshot, with the evolution of forms of CAH, especially institutionalised or scaled up ones, not receiving research attention. Moreover, some of this literature is focused on India, but very little of it on the health space, with but a few noteworthy exceptions that are subnational in scope [35–39].

India’s context is unique but also shares features with other parts of the world. It has a federated governance structure, with the Indian Constitution specifying what components are state subjects (public health, sanitation, hospitals, dispensaries) and what are to be governed by central government (population control) or concurrently by state and central authorities (control of infectious diseases, medical professions, health insurance) [40]. There is a high level of privatization of the health sector in the country, the core design of which was formulated prior to independence using a three-tier structure and dominated, prior to sweeping reforms in 2005, by vertical health programmes [40]. Alongside this, community participation in health planning has been observed since before Independence through a variety of community-based health programs, and has been supported by moves toward decentralization, such as through the 73rd Constitutional Amendment of 1992, which “provided the necessary legal framework for devolution to take place” [41].

Political impetus for reform in India followed about a decade later. Adding onto the foundation for community participation created by various actors, the Indian national government launched the National Rural Health Mission (NRHM) in 2005, aiming to improve the provision of quality, affordable, accountable, accessible, and equitable health care for India’s rural populations, especially among marginalized communities [42].

Communitization was one of five major pillars of NRHM, and while not defined explicitly, is associated with the following statement: “the institutionalizing and scaling up of community led action for health has been a major gain due to civil society pressure and NGO and state level experimentation” [43]. Communitization was intended to institutionalize a rights-based approach to health and the sharing of political, administrative and budgetary decision-making power with village communities. Building on a range of pilots implemented by Civil Society Organizations, it came to be operationalised as ‘community action for health (CAH),’ which in turn comprises a number of processes (see Table 1) of heightening community awareness of health system entitlements, roles and responsibilities of service providers, training of agents of CAH like patient welfare committee members, village health sanitation and nutrition committees with a focus on collection and use of data, use of *jan samvads* or hearings to increase health system accountability [36, 42], the Accredited Social Healthcare Activist (ASHA) programme [1, 36, 44] (see Table 1). Civil society organizations were responsible for creating and building the capacity of these village committees, while the government ensured the involvement of frontline staff in sharing monitoring results and health planning. The findings from these processes were expected to be incorporated into the states’ annual health plans [45]. To facilitate communitization through NRHM, the Indian government constituted the Advisory Group on Community Action (AGCA), whose role, since its establishment in 2005, has been to provide advice and technical support for CAH activities as a committee of the Ministry of Health and Family Welfare (MoHFW) [46].

**Table 1:** Key forms of community action for health in India

Community Action for Health in India encapsulates a number of activities which are summarized below:
Village Health, Sanitation, and Nutrition Committees (VHSNCs) are institutional mechanisms established at the village level to inform communities about health programs and government initiatives, ensuring their active participation in planning and implementing these programs. They serve as platforms for convergent action on social determinants and public services related to health, providing avenues for communities to voice their health needs, experiences, and issues with access to health services, enabling local governments and public health service providers to respond appropriately. They support and facilitate the work of ASHAs and other frontline healthcare providers, contributing to the overall improvement of health outcomes at the village level [47, 48]. This model involved the formation of village health and sanitation committees, capacity building of these committees, and facilitation of their key tasks in community monitoring and planning. Civil society organizations were responsible for creating and building the capacity of these village committees, while the government ensured the involvement of frontline staff in sharing monitoring results and health planning. The findings from these processes were expected to be incorporated into the states’ annual health plans. The project was piloted in nine states (Assam, Chhattisgarh, Jharkhand, Karnataka, Madhya Pradesh, Maharashtra, Odisha, Rajasthan, and Tamil Nadu) with the aim of familiarizing state governments with the process and encouraging them to adopt and upscale [45].
Community-Based Monitoring and Planning (CBMP) is a regular, participatory audit of public health services that facilitates the involvement of community members in assessing the public health system and advocating for improvements. Implemented as a pilot in nine states (Assam, Chhattisgarh, Jharkhand, Karnataka, Madhya Pradesh, Maharashtra, Odisha, Rajasthan, and Tamil Nadu), this approach captures information often missed by routine reporting, such as the presence and availability of doctors in health centers, the conduct of outreach visits, the behavior of health staff towards patients, and instances of illegal charging or prescribing medications for purchase [36].
Launched in 2005 as a key component of India’s National Rural Health Mission (NRHM), the Accredited Social Health Activist (ASHA) programme aimed to strengthen rural government service delivery and enhance community engagement and ownership in health programmes. The programme involved selecting one woman per village (approximately 1 per 1000 population) who received 23 days of initial training on basic health topics. These ASHAs linked community members to health services, provided basic first aid and supplies, and mobilized the community around water, sanitation, nutrition, and health issues. In 2015, the programme matured into the National Health Mission (NHM) and was extended to marginalized urban areas. With nearly one million ASHAs now selected and trained, it has grown to become one of the largest community health worker (CHW) programmes in the world [49].
Patient Welfare Committees (Rogi Kalyan Samitis), also known as Hospital Management Committees, were introduced in 2005 to improve the functioning and service provision in public health facilities. They serve as consultative bodies to enable active citizen participation for the improvement of patient care and welfare in health facilities. The committees include eminent citizens and civil society representatives and are empowered to perforIm social responsibility functions related to patient welfare. They receive annual corpus grants to achieve their objectives, which include ensuring no user fees for certain treatments, deciding on minimal user fee structures, and providing non-clinical services like safe drinking water, clean toilets, and patient navigation support [50, 51]. Under the recent reworking of Health and Wellness Centres as part of India's Ayushman Bharat reforms, these committees have been given purview at higher level facilities (Community Health Centres, District Hospitals), while People's Health Committees (Jan Arogya Samitis) play this role at the primary healthcare facility level (i.e. for Health and Wellness Centres) [52].

NRHM has been described as a landmark flagship programme of the Government of India, as it was associated with reductions in infant mortality, and maternal mortality, although not enough for India to achieve the Millennium Development Goals [53]. Nevertheless, CAH under NRHM in India has had an instructive journey [54]. Since 2005, the establishment of “invited” spaces within formal governance and planning, considering stakeholder needs and empowering key relationships, processes, and learning, has been a significant milestone within the National (Rural) Health Mission (NRHM). As pointed out elsewhere in the literature, NRHM itself was the result of a powerful mobilisation and synergies across advocacy coalitions. Actors from across a range of institutions (the Planning Commission, NGOs, health providers, academic public health experts and international donor agencies) successfully pushed for a more interventionist role in the health sector [55]. The evolution of CAH within the NHM has been nonlinear and variegated, taking many forms across states. High degrees of citizen power are difficult to achieve and sustain, especially in the case of community participation programmes which are process intensive and come to fruition only in the long term. At the same time, over the past decade, holding onto these institutional spaces has proven to be progressively challenging.

Between 2007 and 2009, the central government funded a pilot project of Community Based Mobilisation (CBM)—spearheaded by the AGCA—to help state governments learn about CAH activities and adopt them before spearheading their post-pilot implementation and upscaling [37]. A total of 1,620 villages of 36 districts across the states of Assam, Chhattisgarh, Jharkhand, Karnataka, Madhya Pradesh, Maharashtra, Orissa, Rajasthan, and Tamil Nadu were selected to participate in the pilot phase [56]. Evaluations of this phase found that the CAH activities showed promise; for example, CBM was associated with improved quality and use of village-level health services in multiple states [57]. However, following the pilot, only two of the nine participating states continued implementing CAH activities in accordance with the original model (see Table 1, Community Based Monitoring and Planning). In recent times, across the remaining states, CAH implementation has either been modified drastically or halted entirely [37].

Scholars in India have employed various methodologies and frameworks to understand CAH. For instance, Gaitonde et al. (2017) analysed CAH- related documents and implementers’ testimony using Advocacy Coalition Framework (ACF). Their analysis revealed developments across different stages of policy, program formulation, and the post-pilot phase [38]. For instance, Gaitonde et al.. identified two advocacy coalitions: state-level bureaucrats and NGO representatives. These groups held fundamentally different beliefs about the purpose of CAH, highlighting dissonance between these coalitions. While the [state-level] bureaucrats viewed the communitization process as a tool for gathering information and addressing service gaps, in contrast, the NGOs envisioned CAH as a means to enable a shift in the balance of power in the health sector, in favour of people [38]. During policy formulation at the national level, NGOs had the opportunity to shape the pilot model in alignment with their core beliefs regarding communitization. However, their influence waned in some states after the pilot due to various factors including a lack of trust between the government and NGOs [38]. For instance, in some states, CBM was implemented in a limited way, reflecting bureaucrats’ beliefs about the role of communities- which was primarily seen as identifying service delivery gaps within villages, rather than the broader governance role envisaged by NGOs [38].

Gaitonde, Sebastian and Hurtig (2020) additionally examined the pilot, implementation, and discontinuation of CAH activities in Tamil Nadu, using Atun’s framework [37, 58]. This framework is built on the recognition that the functioning/uptake of health system innovations is dependent upon: (1) their intrinsic characteristics and (2) their compatibility with the implementing system’s characteristics and broader social, cultural, and political contexts [37]. While Gaitonde et al. discovered factors that facilitated CAH integration into Tamil Nadu’s health system, these were eventually overtaken by CAH’s deep complexity as an intervention, divergent perceptions of the policy problem among implementers, incompatible adoption system and broader context characteristics, resulting in intervention termination [37]. Shukla and colleagues further theorize the initial pilots in Maharashtra, noting the use of a sandwich strategy of national advocacy enabling local mobilisation and escalation of problem solving also forging links across levels, but also alliance building between the frontline and community – indeed the lines between supply and demand side quite literally blurring thereby, and knowledge generation and sharing playing a critical role in advocating for change [39].

These works have built our understanding of the state evolution of CAH activities during and beyond the pilot of communitization. What is absent is a national level analysis that draws on the perspectives of a wide range of national players involved with this history. Our study sought to examine temporally the evolution, and way ahead for institutionalised CAH activities at the national level, drawing directly from people who participated in, and were actively involved with shaping the CAH agenda within NRHM.

## Method

### Approach

We used the oral history methodology of Witness Seminars. This methodology, developed by Tilli Tansey and formalised at the Institute of Contemporary British History (now the Centre for Contemporary British History) [59], invites key witnesses of a past event or process to share their perspectives on that event, holding space for participants to debate, dialogue, and agree or disagree with each other’s views. Guided by prompting questions and a chairperson, the method encourages witnesses to raise relevant issues they think pertinent to the event under discussion, acknowledging that more structured questioning can limit the memories and understandings shared. The methodology lends itself for holding space for participant reflections, including room for potential disagreement among participants, recognizing that there are multiple ways in which ‘reality’ can be experienced and understood [60]. As it involves multiple witnesses—in our case, civil society members, retired government officials, researchers, practitioners, and advisors—co-producing historical knowledge, the Witness Seminar methodology was well placed to mitigate the pattern we saw in literature, namely of NRHM’s piecemeal historical documentation and analysis—especially relating to the evolution, impact and scaling up of community accountability processes and models of community participation—stemming from the use of limiting analytical lenses. Our application of this method involved review of the literature to understand methodological aspects, as well as guidance from and interaction with organisers of Witness Seminars in India [61] who have subsequently gone on to create a methodological resource on the conduct of this method [62]. We are mindful, however, of our unique positionality vis a vis this method and domain of inquiry (see Positionality Statement).

### Participant sampling and ethical approval

For the selection of witnesses, one female researcher (MK) supervised by another female senior health systems researcher (DN) identified witnesses involved with community participation and accountability mechanisms within NRHM and at the national level through publicly available sources, researcher networks and snowball sampling. Eligible participants included serving and retired government officials who were involved with NRHM in its early days, members of the Action Group for Community Action (AGCA) throughout its existence, Civil Society Organisation leaders who had been involved with meetings that took place even before the formulation of NRHM, and so on. Prospective witnesses were contacted via e-mail and telephone. In addition to sharing a concept note outlining the theme and approach of the study, one-on-one virtual conversations were held with all the witnesses to align them with the methodology and to gather their feedback on the overall approach to the documentation. Ethical approval for this study was issued by the Institutional Ethics Committee of the George Institute for Global Health (Project Number 27/20).

### Witness seminar tool and recruitment

In the lead up to the seminars, based on guidance from and consultative meetings with witnesses, we developed nine generalised prompting questions on the community-based accountability mechanisms institutionalized nationally under NRHM (Supplementary File 1). The prompts consisted of three questions each across the thematic areas of (1) Emergence, (2) Evolution and (3) Evaluation/impact of CAH within the NRHM (this structure also suggested by a witness who helped guide our process). Upon finalising a mutually suitable date and time with the witnesses, the session format (agenda), prompting questions, participant information sheet and consent forms were shared via email with witnesses and wherever possible, written informed consent emailed back. For individuals who were unable to send their forms beforehand, written informed consent was obtained prior to the actual scheduled meeting, with a verbal confirmation at the start of proceedings.

Two virtual Witness Seminars were organised in November and December 2021 respectively. During the Seminars, after opening remarks by the chairperson, each witness was given 8–10 min to verbally express their narrative(s), before the space was opened to the semi-structured dialogue/responses. One participant shared their reflections via written responses, two participants engaged in both a seminar and a one-on-one interview, and one other participant, who could not participate in the seminar consented to a one-on-one individual interview online. While the seminars were loosely guided by the prompting questions and slotting witnesses according to their experience with the themes, all participants had the flexibility to address questions across the themes in any order. Each seminar was facilitated by a chairperson who was selected considering their involvement and experience with health systems and policy processes in the context of NRHM. Owing to the constraints posed by the ongoing COVID-19 pandemic, we organised the seminars virtually via Zoom. Each Seminar was around 1.5 h in length and was conducted in English.

To help reduce biases introduced by the limited time provided for this exercise, power imbalances between participants, and potential groupthink, all witnesses were provided the option of elaborating on their narratives in one-on-one interviews outside the main Seminar. There were two refusals of participation with one participant requesting re-designation as an ‘anonymous observer’ of the seminar proceedings.

### Audio-visual recordings and transcripts

The seminars and in-depth interviews were recorded and stored electronically on an encrypted network drive at The George Institute for Global Health, New Delhi, India Office. The securely stored recordings were shared with a professional agency that followed due confidentiality protocols to transcribe, in full, the Witness Seminars verbatim in English.

To increase the transcripts and written responses’ suitability for and comprehensibility in the written word, MK and SS made minor edits related to grammar, spelling, etc., in line with recommended practices [63]. The verbatim transcript was then circulated to all Witnesses, who, at this point, could exercise their right to delete, restrict and/or redact their portions as they saw fit. After receiving approval by the witnesses, MK and SS incorporated amendments and the transcripts were annotated with information regarding key events, persons, organizations, sources, and technical terms mentioned by witnesses. The annotated report was then shared with participants for any further modifications as applicable. After this second round of review and approval by the witnesses, the reports were prepared for publication with the support and review of DN and other colleagues (see Acknowledgements).

### Analysis

After the second round of review and approval of the annotated manuscripts by the witnesses, text versions of the reports were entered into ATLAS.ti. Following this, SS, and MK iteratively devised codes corresponding to each of the prompting questions to help categorize witness contributions by question/thematic area. For capturing witness responses to questions beyond the guiding questions, and to be inclusive of new or unexpected topics during the organizational stage, SS and MK added new, inductive codes and definitions over multiple rounds of revision and discussion. This could be broadly categorized as a reflexive thematic analysis approach [64], with a number of coders and analysis coordinating as a group.

Following the initial coding and organization, SS, and MK, in consultation with DN, conducted inductive thematic analysis of the reports, identifying emergent patterns in witness reflections. Analysis-related conversations were held as spaces for reflection on researcher alignment with witness accounts, relationships with witnesses, and role within Witness Seminars (e.g., as moderator), allowing the team to further understand the diverse lenses brought to the analysis. In subsequent sections, we present findings from our combined secondary analysis of both Witness Seminar reports, which include the seminar transcripts and corresponding written/one-on-one interview responses.

## Results

**Fig. 1:**
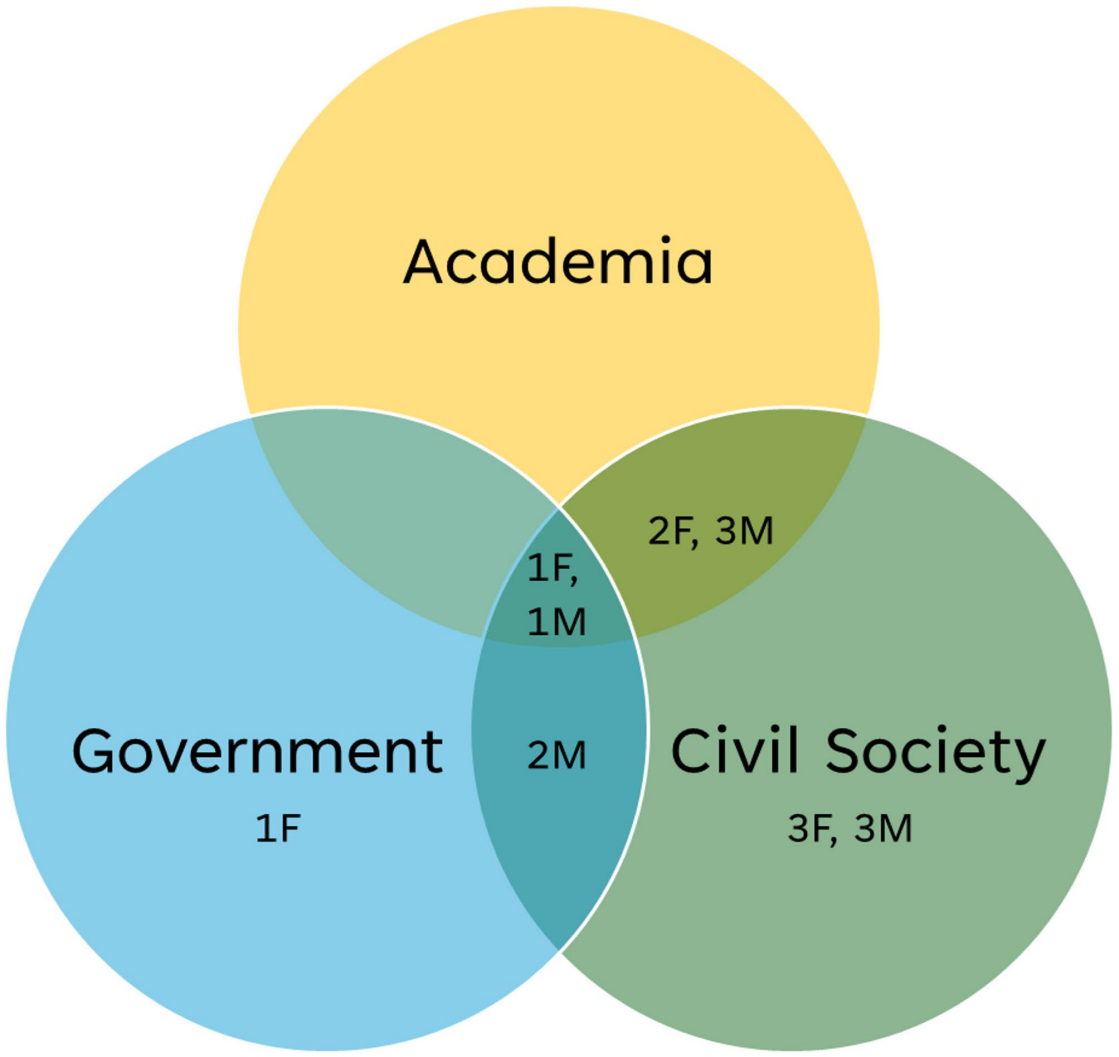
Study participant details (*N* = 17). F-Female, M- Male

A total of 17 participants were involved, of which ten witnesses engaged in in the first seminar, and five in the second and two through an in-depth interview and written comments only. We had representation of academia, government and civil society, with some participants occupying multiple positionalities and a great deal of collaboration (see Fig. 1). For example, one participant had held a position as an academic, a technocrat-administrator supporting community action for health at the national level and also been an active member of national and state level people’s movements working in health. There were also persons who had worked shoulder to shoulder for many years in the early years of NRHM, working to develop guidelines and formulate decisions on the shape of CAH initiatives at the national and subnational level.

To map the evolution of NRHM in India, this paper utilizes the metaphor of ‘communitization’ as a ship to highlight the various phases and aspects of its history. The following sections lay out the evolution of NRHM leading up to the CBMP pilots (up through 2005–2006), expansion of CAH activities across nine states in India with CAH activities eventually pivoting from CSOs to state governments (2006–2009 and up through 2013); and finally, the contemporary situation of CAH in the wake of competing political priorities at the local and national level from 2012 onwards.

### Building the ship: debates and priorities during establishment of CAH

Witnesses indicated that one of the first tasks undertaken by the Ministry of Health and Family Welfare (MoHFW), was the constitution of an Advisory Group on Community Action (AGCA) to provide guidance on community action initiatives under the National Rural Health Mission (NRHM) at the national level. Alongside this, a total of eight task groups were set up within NRHM with one task group solely for community participation. With each of the task groups, emphasis was placed on tracking public accountability and program implementation. As one witness said, “One of the agendas [of incorporating community action in NRHM] was to contribute as well as keep a critical eye on how the [NRHM] implementation was taking place.” Witness 1.

Members within the AGCA advocated for the creation of a Community Based Monitoring and Planning (CBMP) of health services as a key strategy of NRHM. The objective of the CBMP was to place people at the centre of the process for ensuring that the health needs and rights of the community were being fulfilled. This framework was conceptualised such that it would allow active and regular monitoring of NRHM interventions by the communities in their areas.

*AGCA was initially getting bogged down by discussing NGO projects and one of the things that [members] pushed for is that*,* instead of AGCA being a monitor of which grant goes to an NGO which will lead to some members within AGCA applying for the grants having conflict of interest*,* why don’t we design more deliberate accountability processes? AGCA set up a committee and created the framework for community monitoring…[in the subsequent meetings] AGCA presented the CBMP framework… It [CBMP] was kind of pushed in - it was kind of coming together and we turned around the AGCA’s agenda a bit. It [CBMP] started happening in different states*,* and each state started having an anchor person*,* and we worked with the anchor organisations. That is how*,* in many ways*,* from my perspective*,* the pilot program of community monitoring started. – Witness 2*.

Alongside CBMP were various other forms of communitization, including the Accredited Social Health Activist (ASHA) mentoring groups, as well as planning and oversight committees such as the Village Health, Sanitation and Nutrition Committees (VHSNCs), Rogi Kalyan Samitis (Patient welfare committees) and more. However, the question of how these groups would achieve CAH objectives within NRHM remained.

*One major area of contestation in these [communitization] discussions was the tension between defining the role of village committees. Was it making the government health system accountable or reaching entitlements to marginalised people? Or were village committees to undertake collective action in solidarity with health staff*,* (e.g.*,* in vector control). Or were village committee and ASHAs*,* to be low paid extension workers? – Witness 15*.

Inasmuch as various components of CAH were interlinked, these contestations extended not just within but beyond health departments. For example, the VHNCs as committees were designed to have representation from and chaired by the Panchayats. However, this framing was met with resistance from the Panchayati Raj Departments who viewed this an as infringement on the autonomy of the Gram Panchayat standing committees in managing local health facilities.

*I recall the very early discussions with the Village Health Sanitation Nutrition Committee and the ASHAs and the communitization process of NRHM*,* who is not just CBMP. It was ASHAs*,* Village Health Sanitation Nutrition Committee and the CBMP all put together to enhance this communitization process. I think the original intent is*,* one can see from the framework document that the Village Health Sanitation Nutrition Committees’ capacity would be built to serve as the bodies including membership of the Panchayat*,* chaired by the Panchayat. There was so much fierce resistance in the early 2005*,* ‘06*,* ‘07 from the Panchayati Raj department saying why do you want to create a VHSNC when you also have the standing committee of the Gram Panchayat to serve this goal? – Witness 6*.

### Setting sail through state variation, ownership and scale-up

From the get-go, there was a formal role for CSOs to support, train, handhold and facilitate communitization processes nationally, The MoHFW’s National Health Systems Resource Centre (NHSRC) was directed to collaborate with CSOs to oversee the involvement of NRHM’s sub-committees and provide technical assistance to states for CAH rollout. There was strong alignment of the leadership of the time with the mandate and NRHM in general, and of communitization specifically.

Beyond the national level, the way CAH mechanism’s function varies by state. In some states, a primary CSO at the state level works closely with the state’s health department to manage the programs. In other states, there are community process that support units under the state health missions that manage CAH [36]. As a consequence, given that health is a state subject, it was not unexpected that the rollout of the various components of communitization was highly variable and customized across Indian states:

*We tend to make a general statement about the entire programme*,* but actually there is difference between states in how the programs were understood*,* implemented and the way they went*,* even after the second phase expansion. Himachal*,* for example*,* came into the community processes and the ASHA program very late. Goa came into it even later. Uttar Pradesh had one particular take on it and took a long time to decide on whether to expand. In fact*,* they insisted that the state must be allowed to do local modification*,* state-level modification of the material. They took the material and removed every little bit on gender that was there. There wasn’t much that was there on gender. At some point you had all sorts of things going on across states and sometimes it was progressive. There are very interesting things that you can get as feedback and learnings from one state to the other. – Witness 15*.

The variation across states reflects the existing networks, activities, and power possessed by community-based organisations and civil society actors. It also is an artifact of India’s larger governance architecture within which communitization is nested: since health is constitutionally designated a state subject. Variation in *how* even a national program would be operationalised, is by design, expected.

Notwithstanding this state-specific variation, witnesses noted the seriousness with which the communitization process was integrated into NHM processes. Witness 8 pointed out that by 2010, communitization began to be reflected in Program Implementation Plans (PIPs) with dedicated allocations in state budgets: “The Program Implementation Plans (PIPs) were being organised in a very robust, structured manner. The discussions were very detailed and very enlightening, both for the States who are coming to present their plans and for the Government of India, which was to give the funds. I believe I could see the process of decentralisation actually being born in those [Program Implementation Plan] discussions and become stronger and stronger.” Witness 8.

However, witnesses also noted that despite the progress of CAH in certain states and with the AGCA providing technical support, overall, there was “a simultaneous absolute constriction of participation at the national level -hyper-centralisation- and also in most states, state governments also moved into a military mode, and the State Health Department, State Health Minister, Health Secretary, and maybe a few other close circle officials essentially took over all decision making and blocked all other kind of inputs.” - Witness 3.

#### Floundering waters - ‘communitization’ versus ‘sarkarikaran’

From 2012, with the scaling up process, the CBMP component of CAH began to encounter challenges. In a sense, a part of the ‘communitization’ of government processes was a process of ‘governmentalisation’ of community processes; one witness referred to this as “sarkarikaran.” The word *sarkarikaran* literally means governmental work suggesting the integration and enmeshing of the community action agenda in the rituals and institutional design of government. Witness 2 explained it thus: “as Civil Society was part of the Mission Steering Groups - all the operational parts became sarkarikaran-ed, even NHSRC became sarkarikaran-ed.” Witness 2. Witness 3 lamented this change, saying: “If the state starts shaping actions by communities, rather than community shaping action by the state, then the spirit of communitization is finished. That is sarkarikaran [government(alised) work,] not communitization.”

A prime example of this was the eventual shift in the roles and power dynamics between community entities like VHSNCs comprising community members, local representatives and frontline health providers, convened by Accredited Social Health Activists (ASHAs) [47]. These would at times serve not as community actors, but as ‘sub-contractors’ and local implementers of NHM.

*One part of communitization has been seeing how untied funds and other funds have been given over to VHSNCs*,* Rogi Kalyan Samitis and equivalently named entities. …. From being a body that is meant to assess whether a health facility is doing well or badly*,* and to suggest improvements*,* this body instead becomes more of a local implementing agency. The accountability relationship reverses*,* and the tables turn when the health department says*,* “Look*,* have you spent the money correctly because the auditor is asking*,* and have you followed the rules?” – Witness 4*.

While these existing community structures and processes appear to have a flipped accountability, the role of CSOs appears to have waned in the past decade. Another witness raised,

*The whole involvement of NGOs in NHM or NRHM actually faltered after about 2012-13. We have been part of developing guidelines for NGO involvement for over a period*,* and originally the NGO involvement guidelines were very focused towards strengthening community processes. Over time*,* they became much more towards strengthening service delivery*,* especially primary healthcare*,* and now they have disappeared from the scene. I think the last guideline for NGO engagement was written in 2013 and that is gone*,* and nobody is questioning why 5% of the funds of the NHM that were meant to be for NGO support are actually being used…. NGOs have merged into the private sector as well. So private - for profit*,* private - not for profit has all been amalgamated into one whole.” -Witness 6*.

This reduction in roles of and funding for CSOson the one hand, and reframing of civil society as private sector on the other, appears to have paved the way, witnesses noted, for public-private partnerships focused on service delivery. Witness 9 remarked that budgets for the AGCA secretariat had reduced and required subsidization for about 50% of costs. “So, do we take external funding, but then it dilutes government ownership accountability in a way. There is the funding question and dilemma that AGCA experiences.” Witness 1 noted that “this funding issue is also very critical for the survival of CSOs. Because I think with the changes in FCRA [Foreign Contribution (Regulation) Act, which governs the receipt of foreign funds for Indian entities], there is quite a bit of flux within the Civil Society Organisations. There are great concerns about their future, their survival, including, you know, what sources of funding they can access and how it can be utilised. At this moment, we are in a certain kind of a flux…” It was pointed out that funding allocations to states for facilitating CAH have been increasing (up from [Rs.] 270 crores (roughly 32 million USD) in FY20-21 to [Rs.] 342 crores in financial year 2021-2022yet the programming that is funded is in the direction of privatisation of social services through public-private engagements. A witness pointed out how ‘public-private engagement’ appeared to have eclipsed communitization in most recent programming:

*Coming to the contemporary situation*,* what are the lessons for the Swasth Bharat Yojanas [PM Atmanirbhar Swasth Bharat Scheme] that are being rolled out. Largely*,* you will have to realise that these new Yojanas that are coming out fill gaps existing in public health infrastructure especially in primary and critical care facilities. They also look at IT-based surveillance systems*,* diagnostic labs*,* lab-based surveillance systems and empowering primary workforce to deal with emergencies that may arise. There is no debating about the importance of this. However*,* communitisation has sort of been replaced in these schemes by a new buzzword - ‘public-private engagements. ‘Public-private engagements’ is the new buzzword with the word ‘communitization’ jargon seems to be slightly in the background*,* if not completely out. So [with regard to] communitisation*,* no debating [it]*,* is essential for any Yojanas and schemes*,* even if one is infrastructural and technical leading one. – Witness 12*.

#### Flowing where there’s space (or even when there’s not) to address emerging challenges

The prognosis of institutionalised CAH in India was somewhat bleak from the perspective of many of the witnesses. However, as one of the Witness noted, the fate of CAH does not need to be tied to this particular history of “communization” – not in the past, nor going forward:

*Rights based based community action for health has been a constant throughout this process. It is like flowing water which will continue to flow even if there are obstacles whereas the state support for the community accountability has been variable and depends on the wider political climate. As far as the social accountability processes are concerned*,* communities are primary and state is secondary*,* and it is not the other way around and I would like to emphasise this point. Community action will continue whether there are spaces or not*,* but the state’s ability to digest and appetite to tolerate community action might be quite variable. - Witness 3*.

From the perspective of another witness, in contrast, there had been a handover to a different type of “communitized” government structure in some states.

*So*,* from the pilot phase*,* which was primarily led by CSOs*,* state governments have taken initiative to scale up implementation of community action processes through state level institutions. I would like to take the examples of State Health Resource Centres in Chhattisgarh and Odisha as well as Kerala Institute of Local Administration (KILA) where systematic community monitoring and action are being done at scale. [an observer] also mentioned the social audit carried out by the Department of Rural Development. The AGCA has facilitated partnerships between the State Social Audit Units and National Health Mission Meghalaya*,* Uttarakhand and in Jharkhand to conduct social audit of health services*,* which has been very promising. – Witness 10*.

Witness 12, looking ahead, noted that there were new and emerging challenges for civil society and for community action for health initiatives to tackle; she called for “broader sensitisation of the community of the workforce at the grassroots and taking into account not only the traditional persistent health threats but newer emerging ones which cannot be limited today to only in the local context. We can refer to climate change, antimicrobial resistance or the pandemic we are living through.”

## Discussion

### Broadly what we found

Nautical themes are quite common in the health systems governance context, given the etymology of the word “governance” itself (it derives from the Greek root for the word “steer” in the context of sea navigation) [65]. It is worth mentioning another reflection on CAH in India uses the metaphor of “sailing below the waves” where “most of the time we sailed in the deep dark troubled waters far below the surface? We didn’t sail following a straight pre-planned route but meandered, guided by our compass, by wind and stars, and by opportunities and obstacles. We are still underway towards our final destination [66]. ” So could also be said of the journey reflected upon in our Witness Seminars at the national level under India’s flagship NRHM reform process.

Our analysis of India’s ‘communitization’ process saw that as the CAH scaling up process progressed from 2012, there was a shift from ‘communitization’ towards “governmentalization” of community processes. Civil Society Organizations (CSOs) that were part of Mission Steering Groups became more integrated into government operations, where government processes absorbed community initiatives. The roles and power dynamics of community entities, such as Village Health and Sanitation Committees (VHSNCs) and Accredited Social Health Activists (ASHAs), evolved. Initially aimed towards strengthening community processes and accountability, after 2012–2013, the focus of NHRM’s guidelines shifted towards service delivery.

We theorise therefore that at the national scale, the sheer lack of human resources in service delivery result in a concentrated focus of CAH mechanisms – with community actors having to take more and more responsibility for this – whether formations like VHSNCs or RKS or NGOs. This has turned them into *providers* of care and thereby enabled the conflation of their role as the “private sector.” The closing of funding avenues for civil society and the provision of institutionalised (ironically named untied) funding has increased, in turn, increasing the reliance on government structures. This has had the effect of increasing deference to supply side priorities and reducing the attention to and flexibility required of a focus on demand side priorities.

At smaller scales, however, there remain spaces and formulations that allow for a focus on demand side priorities – particularly emerging ones like climate change and antimicrobial resistance – that may be where the flow of CAH currently rests, or flows. Critically, in these spaces, there are new problems, new knowledges to generate and share. That said, as another analysis points out “it’s up to the ‘indigenous actors’ at the different levels, from community up to national level to really sustain something which has the potential of being sustainable. It’s their choice since they are the owners of the learnings and doings which emerged” [66].

### Sailing into governmentality: changed roles and positionality of community actors

Frameworks by Abimbola et al. and Brinkerhoff et al. are relevant in emphasising the crucial role of non-state actors in health governance [67, 68]. These frameworks highlight the importance of multi-level governance in achieving social accountability and CAH goals. Abimbola et al. identify three levels within the health system: the operational level (comprising citizens and healthcare providers), the collective level (encompassing community groups and coalitions), and the constitutional governance level (governments at various tiers). This framework highlights the significance of evaluating governance beyond a single level. Operational and collective governance play a vital role, particularly when constitutional governance falters. Importantly, this framework positions actors at all levels as “potential governance practitioners,” including community groups and coalitions. Similarly, health system governance frameworks by Brinkerhoff et al. [7, 69, 70] underscore the importance of interactions among these three levels and their impact on service delivery. They conceptualize governance as an outcome of interactions between “principals” (citizens) and “agents” (state and healthcare providers) at these levels, who may possess diverse interests.

However, as seen in our study, challenges arise when the constitutional level begins to overpower and control the collective level, and when the operational level blurs boundaries between citizens, community organizers and providers, turning them all into providers. This, we believe is what is happening with ‘governmentalization’ or “sarkarikaran’ of communitization of health in India over time. This enmeshment erodes the distinction between the levels, and vexes the “rules of the game” [65, 67]. Just as principals and agents play distinct roles and have diverse, sometimes conflicting interests, so too do the different levels of governance. This separation is crucial because with governmentalization, agents (state and healthcare providers) may overstep their bounds by becoming too involved in the affairs of principals (citizens) and taking over their roles. Furthermore, the extent of conflation of the three levels differs in each state. The dilemma lies in finding a balance: each level of governance needs to be valued and resourced, but each also needs to remain distinct and retain its own value. The muddling of relationships between and within each of these levels raises concerns about the community becoming a mere extension of the government, a private (non profit) provider, leading to issues of representation, legitimacy, and tokenism in health governance. As our study of the NRHM case demonstrates, this can lead to the construction of an (unnecessary) tension between ‘efficiency’ (in health system delivery) and ‘equity’ (read as community ownership) [67, 69, 71]. As part of this, involvement of communities is seen as the contribution of “private” provider to optimizing ‘efficiencies’ on the supply side, with little recourse or limited *locus standi* to issue critique, seek grievance on the demand side.

‘Sarkarikaran’ or ‘governmentalization’ provides a valuable lens to explore the scholarly debate concerning the shift from ‘governance’ to ‘governmentality’ [72, 73]. In the context of CAH, this shift can be characterized as a dual transition. The first transition marks a movement from implicit state control or ‘governing’ towards a more formalized system of ‘governance’ achieved through collaboration of state and non-state actors, i.e. dispersion of accountability from the constitutional level to the operational and the collective level. However, as exemplified above by Witness 12 above, for instance, the rise of public-private partnership, becomes an exemplar of governance as not being focused merely on the state but also as being dispersed multisector actors and agents, rather than solely the State [74]. The second transition, fuelled by the increasing influence of government structures and processes, with the melding of all three levels of governance i.e. the operational, collective and constitutional can be traced as a shift from ‘governance’ to ‘governmentality’.

This shift to ‘governmentality’ or sarkarikaran carries the inherent risk of marginalising CSOs and diluting the core principle of citizen-centric engagement. Here, it is crucial to recognize that communitization does not merely exemplify the transference of power from the State to CSOs. Rather, it represents a more systematic reshaping and redefinition of civil society itself. This reshaping renders civil society ‘both as an object and subject of government’, a dynamic evident in the case of VHSNCs and ASHAs discussed above [72]. Thus, the trajectory from ‘governing” to ‘governmentality’ can enter communities, civil society organizations (CSOs), and community-based organizations (CBOs) into a vicious cycle of co-optation into the “supply” side. Roychoudhury makes similar observations of how claims-making for survivors of domestic violence has transformed through in a context of neoliberal governmentality, where they are recast as caseworkers for their own survival by leveraging their organizational connections [75]. Pyysiäinen and colleagues call this the responsibilization – where we would call it the responsibilisation of principals such that they become agents [76].

### Navigating (away from) governmentality - Embeddedness and state-society accountability relationships over time

Another key aspect of what we find is the temporal element: community action for health in India has undergone a significant evolution, from being scaled up from state-specific programs spearheaded by individual states to a national initiative. While national policies require accountability, as discussed above, the implementation of CAH has been inconsistent and scaling this approach nationwide has presented challenges. Bussu et al. (2022) underscore the temporality of participatory governance noting that institutionalisation is when we cause “participation to be formalized into a permanent and cyclical structure, whilst failing to embed within the broader political or policy systems it is intended to connect with.” [77] They argue instead for “embeddedness” of community participation, drawing on examples in Catalan Spain, Brazil, and Scotland involving social experimentation, fragility and “constant political work” [77]. Embeddedness (with its legacy in Polanyi’s economic sociology), involves a constant negotiation between invented and invited spaces, and invites inquiry into various forms, segments, periods and windows of productive interaction between governance actors, principals and agents, rights holders and duty-bearers.

As we explored the vicissitudes of India’s experience, according to scholars, may be rooted in different interpretations of ‘accountability’, reflected in the variation in how it is implemented [36, 78, 79]. While some note that the idea of fuzzy definitions and ambiguity allow for garnering of political consensus [31, 39, 80]. With health being a state subject, variation in definition and implementation is almost guaranteed, with fuzzy definitions affecting operational exigencies. One could ask if a more effective strategy would involve documenting and learning from state-run programs, rather than what they signify as an institutional whole at the national level. In doing this, we are able to acknowledge that the National Rural Health Mission (NRHM) is an example of “openings from above” – which Fox and colleagues argue are “invited spaces created from above can be claimed and transformed from below” [35]. An academic focus on ‘aligning’ or ‘standardizing’ such efforts raises dilemmas of governmentality, and thereby, of accountability, and yet still having some local spaces to flow suggest that there may be an enduring power shift where space for ‘communitization’ is not fully centralized, absorbed nor co-opted over time [81–86].

### Implications for policy

Our analysis suggests that a plural approach to community action for health is needed, but also one with more trust and resources reposed in such initiatives at subnational levels. Witness accounts underscored the pressing need to scale up CSO and CBO engagement in health across the country, generate additional evidence, strengthen institutional mechanisms, and support the activities therein through sustainable financing. The discussions on strengthening the base for the participation of CSOs and CBOs beyond the Advisory Group on Community Action could be expanded further, which could prove important for lessons and opportunities, both to improve governance and accountability in the health sector and to accelerate progress towards UHC. In addition to the role of CSOs and CBOs, there may be an emerging role for parastatal organisations like State Health Systems Resource Centres and decentralised governance entities like the Kerala Institute for Local Administration, which have been at the frontlines of developing and operationalising community action initiatives in various forms and for fairly long periods of time.

### Implications for research

There is limited research that tracks the longer term trajectories of community action for health initiatives. This is in part due to the demands of method and positionality of researchers themselves; newer approaches like coincidence analysis may offer some promise [87]. Longer term studies on community action for health are needed, particularly ones that can identify, mindful of context, the mechanistic drivers of these initiatives and the outcomes they effectuate through linked and complex pathways. Further research should delve more into the perspectives not just of brokers and “witnesses” of reform processes for CAH, but CAH initiatives themselves – i.e. the community members doing the action for health! A greater breadth of perspective from government actors would also be critically important and may yield much more nuance to our theorization of governmentality, indicating what pressures drive toward it, and what interpretations exist in government with respect to sarkarikaran. Based on the model, these perspectives may be quite variable and involve different types of constituencies in variable ways – capturing and contextualising this variation could be a major contribution that research is able to provide.

### Limitations and strengths

This study aimed to address the acknowledged need for documentation of CAH experiences and evidence relating to its evolution under the NHM. The study spotlights key learnings from the progression of CAH within NHM India that may have policy implications for implementing such initiatives in other low-and-middle income countries across the world. In conducting this study, there were certain limitations. A Witness Seminar – by design – will constrain overall participation to those who were most visible or known to be part of a history, those who are able to align schedules, and those may feel comfortable participating together in a format of this sort. This privileged more “expert” or “senior” persons in this history, those who are alive and able to participate, and those who primarily could communicate in English. Further, the online format of the witness seminars format, owing to the constraints posed by the COVID-19 lockdown in India reduced the time witnesses had to provide their reflections. Each witness was given 8–10 min to verbally express their narrative(s), to gather the detailed histories of the topics under discussion. Holding a longer seminar may have allowed for a richer discussion. Secondly, due to significant variations of CAH across states, witnesses found it difficult to broadly generalise its evolution as a whole at the national level. Relatedly, a chronological account was rarely produced in these witness seminars and, instead, the past, present and future direction of CAH were amalgamated into the conversation with each other. Regardless of the extent to which the topic guide was followed, its circulation gave participants a sense of how we understood the issue under discussion, and how we envisioned the seminars as a whole. Lastly, our analysis is but a slice of how CAH has evolved under the aegis of NHM – there are certainly more parts and nuances to its history that the constraints of this methodology and time did not allow us to explore.

For instance, we were not able to delve too deeply into how community, social accountability and participatory governance of health systems are among the key elements for Universal Health Coverage (UHC). We were also not able to explore the intricacies of the crucial role CSOs and CBOs play in developing accountability mechanisms in countries with mixed healthcare systems (i.e. those in which out-of-pocket payments and market provision of services predominate as a means of financing and providing services in an environment where publicly financed government health delivery coexists with privately financed market delivery. In their recent publication - Allotey and colleagues highlight the importance of the political, social, and environmental contexts in enabling the success of community engagement as a mechanism to make progress toward UHC [9].

These will be explored in newly initiated trajectories of work these authors have underway, in partnership with the Civil Society Engagement Mechanism for Universal Health Coverage (UHC) 2030 (see www.spheretogether.org) – seeking to use a realist approach that seeks to both understand and intervene upon contexts, mechanisms and outcomes of community action for health (this project is called PATANG and is supported by the India Aliance). We will explore community participation as a *process* to influence better health outcomes while dealing with issues of power or control, representation, resource and sustainability, the importance of which has been emphasised recently by Haldane et al. [27]. In conjunction, this and future analyses seek to strengthen the case for citizen engagement in health to support global advocacy movements towards advancing community participation for achieving UHC.

## Conclusion

Our study analysed a pair of Witness Seminar transcripts to understand the evolution of community action for health in India. We found four moments in this evolution. The first, of “building the ship,” comprised a stage of institution-building in the early days of the National Rural Health Mission, as models were being established and governance modalities were put in place; at this point there was a lot of debate over what the design of mechanisms should be and resistance from local governance institutions. Following this initial phase, as the model(s) “set sail,” there was great variation and another set of tensions between central and state-specific priorities and processes. These tensions came to a head in the “floundering waters” stage where governmentality appears to have vitiated the spirit and momentum of community action for heath, turning it into ‘sarkarikaran’ or government(alized) work. Community action is seen as flowing water, that can goes beyond floundering waters to find others shapes and pathways to move forward. Documenting these journeys of community action for health at the national level opens up many questions about subnational processes, invites comparison with such efforts and initiatives internationally, with demands of method and process (involving academic-advocacy partnerships). These authors are already navigating these waters; we invite fellow sailors and navigators!

## Supplementary Information

Supplementary Material 1.

Supplementary Material 2.

Supplementary Material 3.

## Data Availability

All data used in this manuscript is available at this link: https://www.georgeinstitute.org/our-impact/policy-and-recommendations/community-action-for-health-evidence-from-india

## Abbreviations

AGCA: Advisory Group on Community Action
ASHA: Accredited Social Health Activist
CAH: Community Action for Health
CBMP: Community-Based Monitoring and Planning
CBO: Community-Based Organization
CSOs: Civil Society Organizations
KILA: Kerala Institute of Local Administration
NGOs: Non-Governmental Organizations
NHM: National Health Mission
NRHM: National Rural Health Mission
RKS: Rogi Kalyan Samiti
UHC: Universal Health Coverage
VHSNC: Village Health, Sanitation and Nutrition Committee

## References

[1] Seshadri SR, Parab S, Kotte S, Latha N, Subbiah K. Decentralization and decision space in the health sector: a case study from karnataka, India. Health Policy Plann. 2016;31(2):171–81. 10.1093/heapol/czv034 25967105

[2] International Conference on Population and Development (ICPD). Available from: https://www.unfpa.org/events/international-conference-population-and-development-icpdhttps://www.unfpa.org/events/international-conference-population-and-development-icpd. Cited 2022 Sep 6.

[3] History. and Evolution of Non-Aligned Movement. [cited 2022 Sep 6]. Available from: https://mea.gov.in/in-focus-article.htm?20349/History+and+Evolution+of+NonAligned+Movement.

[4] Gathering a body of global agreements. Available from: http://www.un-documents.net/s6r3201.htm. Cited 2022 Sep 6.

[5] Ciccone DK, Vian T, Maurer L, Bradley EH. Linking governance mechanisms to health outcomes: A review of the literature in low- and middle-income countries. Soc Sci Med. 2014;117:86–95. 25054281 10.1016/j.socscimed.2014.07.010

[6] Ogden J, Morrison K, Hardee K. Social capital to strengthen health policy and health systems. Health Policy Plann. 2014;29(8):1075–85. 10.1093/heapol/czt087 24277736

[7] Brinkerhoff DW, Wetterberg A. Gauging the effects of social accountability on services, governance, and citizen empowerment. Public Adm Rev. 2016;76(2):274–86.

[8] Edward A, Osei-Bonsu K, Branchini C, Yarghal TS, Arwal SH, Naeem AJ. Enhancing governance and health system accountability for people centered healthcare: an exploratory study of community scorecards in Afghanistan. BMC Health Serv Res. 2015;15(1):1–15. 26227814 10.1186/s12913-015-0946-5 PMC4521484

[9] Allotey P, Tan DT, Kirby T, Tan LH. Community engagement in support of moving toward universal health coverage. Health Syst Reform. 2019;5(1):66–77. 30924744 10.1080/23288604.2018.1541497

[10] WHO TEAM. Voice, agency, empowerment - handbook on social participation for universal health coverage 2021. Available from: https://www.who.int/publications-detail-redirect/9789240027794. Cited 2021 Oct 1.

[11] Chan Kman. Civil Society and Social Capital in China. In: Anheier HK, Toepler S, editors. International Encyclopedia of Civil Society. New York, NY: Springer US; 2010. pp. 242–7. Available from: 10.1007/978-0-387-93996-4_735. Cited 2025 Apr 29.

[12] Koonin J, Mishra S, Saini A, Kakoti M, Feeny E, Nambiar D. Are we listening? Acting on commitments to social participation for universal health coverage. Lancet. 2023;402(10416):1948–9. 37738996 10.1016/S0140-6736(23)01969-4

[13] Cornwall A, Scoones I, editors. Revolutionizing development: reflections on the work of Robert chambers. London: Routledge; 2022. p. 336.

[14] Mansuri G, Rao V, Localizing Development DC. The World Bank; 2013. Available from: https://www.worldbank.org/en/research/publication/localizing-development-does-participation-work. Cited 2025 Apr 30.

[15] Gaventa J, Valderrama C, Participation. Citizenship and Local Governance. In 1999. Available from: https://www.uv.es/~fernandm/Gaventa,%20Valderrama.pdf.

[16] Cooke PB, Kothari U, editors. Participation: The New Tyranny? 4th ed. edition. London; New York: Zed Books Ltd; 2001. 224 p.

[17] Williams G. Evaluating participatory development: tyranny, power and (Re)Politicisation. Third World Q. 2004;25(3):557–78.

[18] Place-bound planning support systems for deliberation. Affording better communication and comprehension - Raz Weiner, Filipe Mello Rose, Batel Yossef Ravid, Jörg Rainer Noennig, Meirav Aharon-Gutman, 2024. Available from: https://journals.sagepub.com/doi/abs/10.1177/23998083231217784. Cited 2025 Apr 29.

[19] Beard VA, Miraftab F, Silver C. Planning and decentralization: contested spaces for public action in the global South. London; New York: Routledge; 2008. xiv + 233.

[20] Innovative Citizen Participation and New Democratic Institutions. 2020. Available from: https://www.oecd.org/en/publications/innovative-citizen-participation-and-new-democratic-institutions_339306da-en.html. Cited 2025 Apr 29.

[21] Zhang Y, Xiao X, Cao R, Zheng C, Guo Y, Gong W, et al. How important is community participation to eco-environmental conservation in protected areas? From the perspective of predicting locals’ pro-environmental behaviours. Sci Total Environ. 2020;739:139889. 32534312 10.1016/j.scitotenv.2020.139889

[22] Hauck J, Stein C, Schiffer E, Vandewalle M. Seeing the forest and the trees: facilitating participatory network planning in environmental governance. Glob Environ Change. 2015;35:400–10.

[23] Waddington H, Sonnenfeld A, Finetti J, Gaarder M, John D, Stevenson J. Citizen engagement in public services in low- and middle‐income countries: A mixed‐methods systematic review of participation, inclusion, transparency and accountability (PITA) initiatives. Campbell Syst Rev. 2019;15(1–2):e1025. 37131473 10.1002/cl2.1025 PMC8356537

[24] Nakkash R, Abla R, Saleh R, El Jardali F. Citizen engagement in health policymaking: challenges and recommended solutions. Eur J Public Health. 2022;32(Suppl 3):ckac131539.

[25] Accountability, and Monitoring in Health Initiative, Open Society Foundations. Summary report of the proceedings from the Practitioners Convening on Community Monitoring for Accountability in Health held in Johannesburg, South Africa from 18th– 20th July 2011. Open Society Foundations; 2011.

[26] Cyril S, Smith BJ, Possamai-Inesedy A, Renzaho AMN. Exploring the role of community engagement in improving the health of disadvantaged populations: a systematic review. Global Health Action. 2015;8(1):29842. 26689460 10.3402/gha.v8.29842 PMC4685976

[27] Haldane V, Chuah FLH, Srivastava A, Singh SR, Koh GCH, Seng CK, et al. Community participation in health services development, implementation, and evaluation: A systematic review of empowerment, health, community, and process outcomes. PLoS ONE. 2019;14(5):e0216112. 31075120 10.1371/journal.pone.0216112 PMC6510456

[28] Gaventa J, Joshi A, Anderson C. Citizen action for accountability in challenging contexts: what have we learned? Dev Policy Rev. 2023;41(S1):e12697.

[29] Gaventa J. Power and participation. In: revolutionizing development. Routledge; 2022.

[30] Head BW. Community engagement: participation on whose terms?? Australian J Political Sci. 2007;42(3):441–54.

[31] Fox J. Accountability Keywords. Accountability Research Center; 2022. Available from: https://accountabilityresearch.org/publication/accountability-keywords/. Cited 2025 May 1.

[32] Karuga R, Kok M, Luitjens M, Mbindyo P, Broerse JE, Dieleman M. Participation in primary health care through community-level health committees in Sub-Saharan africa: a qualitative synthesis. BMC Public Health. 2022;22(1):1–17. 35183154 10.1186/s12889-022-12730-y PMC8858504

[33] Danhoundo G, Nasiri K, Wiktorowicz ME. Improving social accountability processes in the health sector in sub-Saharan africa: a systematic review. BMC Public Health. 2018;18(1):497. 29653531 10.1186/s12889-018-5407-8 PMC5899409

[34] Kilewo EG, Frumence G. Factors that hinder community participation in developing and implementing comprehensive Council health plans in Manyoni district, Tanzania. Glob Health Action. 2015;8. 10.3402/gha.v8.26461. 10.3402/gha.v8.26461 PMC4452651 26037041

[35] Fox J, Sullivan Robinson R, Hossain N. Pathways towards power shifts: State-society synergy. World Dev. 2023;172:106346.

[36] Lahariya C, Roy B, Shukla A, Chatterjee M, Graeve HD, Jhalani M, et al. Community action for health in india: evolution, lessons learnt and ways forward to achieve universal health coverage. WHO South-East Asia J Public Health. 2020;9(1):82–91. 32341227 10.4103/2224-3151.283002

[37] Gaitonde R, San Sebastian M, Hurtig AK. Dissonances and disconnects: the life and times of community based accountability in the National rural health mission in tamilnadu, India. BMC Health Serv Res. 2020;20(1):1–12. 10.1186/s12913-020-4917-0 PMC7003366 32024516

[38] Gaitonde R, San Sebastian M, Muraleedharan VR, Hurtig AK. Community action for health in india’s National rural health mission: one policy, many paths. Soc Sci Med. 2017;188:82–90. 28732238 10.1016/j.socscimed.2017.06.043

[39] Activating, Spaces. Scaling Up Voices: Community-based Monitoring and Planning of Health Services in Maharashtra, India. Accountability Research Center. Available from: https://accountabilityresearch.org/publication/activating-spaces-scaling-up-voices/. Cited 2025 Apr 17

[40] Selvaraj S, Karan A, Srivastava S, Bhan N, Mukhopadhyay I. India health system review. New Delhi: World Health Organization, Regional Office for South-East Asia; 2022 [cited 2025 Apr 15]. Available from: https://apo.who.int/publications/i/item/india-health-system-review

[41] The Constitution (Seventy-third Amendment) Act. 1992| National Portal of India. Available from: https://www.india.gov.in/my-government/constitution-india/amendments/constitution-india-seventy-third-amendment-act-1992. Cited 2022 Sep 6.

[42] National Rural Health Mission:. National Health Mission. Available from: https://nhm.gov.in/index1.php?lang=1&level=1&lid=49&sublinkid=969. Cited 2022 Sep 6

[43] Communitization| SOCHARA. Available from: https://www.sochara.org/perspectives/Communitization. Cited 2023 Feb 6.

[44] Community Action for Health. https://nrhmcommunityaction.org/.

[45] Gaitonde R, DIVERGENCES. DISSONANCES AND DISCONNECTS Implementation of Community-based Accountability in India’s National Rural Health Mission. [Umea]: Umea University; 2020. Available from: http://umu.diva-portal.org/smash/record.jsf?pid=diva2%3A1390255&dswid=5739 10.1186/s12913-020-4917-0 PMC7003366 32024516

[46] AGCA–. Community Action for Health. Available from: https://nrhmcommunityaction.org/agca/. Cited 2022 Sep 6.

[47] Ved R, Sheikh K, George AS. VR R. Village Health Sanitation and Nutrition Committees: reflections on strengthening community health governance at scale in India. BMJ Glob Health. 2018;3(Suppl 3). Available from: https://www.ncbi.nlm.nih.gov/pmc/articles/PMC6195149/. Cited 2020 Nov 23 10.1136/bmjgh-2017-000681 PMC6195149 30364368

[48] National Health Mission. Village Health Sanitation and Nutrition Committee. Available from: https://nhm.gov.in/index1.php?lang=1&level=1&sublinkid=149&lid=225

[49] Scott K, George AS, Ved RR. Taking stock of 10 years of published research on the ASHA programme: examining india’s National community health worker programme from a health systems perspective. Health Res Policy Sys. 2019;17(1):29. 10.1186/s12961-019-0427-0 PMC6434894 30909926

[50] Press Information Bureau. Patients’ Welfare Committees. Government of India, Ministry of Health and Family Welfare; 2019. Available from: https://pib.gov.in/newsite/PrintRelease.aspx?relid=192105.

[51] National Health Mission. Guidelines for Rogi Kalyan samities in public health facilities. Ministry of Health & Family Welfare, Government of India; 2015.

[52] National Health Mission. Jan Arogya Samiti: Handbook for Members. NewDelhi: National Health System Resource Centre: National Health Mission; 2023. Available at: https://nhsrcindia.org/sites/default/files/2023-04/Jan%20Arogya%20Samiti%20Handbook%20for%20Members%202023.pdf. Accessed 12 Apr 2025.

[53] Hove J, D’Ambruoso L, Mabetha D, Van Der Merwe M, Byass P, Kahn K, et al. Water is life: developing community participation for clean water in rural South Africa. BMJ Global Health. 2019;4(3):e001377. 31263583 10.1136/bmjgh-2018-001377 PMC6570987

[54] Mukherjee K. Selective universalism: the Paradoxical strategy to achieve universal health coverage in India. J Health Manage. 2019;21(1):154–9.

[55] Community Action for Health. Available from: https://nrhmcommunityaction.org/. Cited 2022 Sep 6

[56] Singh S, Das A, Sharma S, Parija E, Ghosh M. Reviving Hopes Realising Rights A Report on the First Phase of Community Monitoring under NRHM. 2010.

[57] Atun R, De Jongh T, Secci F, Ohiri K, Adeyi O. Integration of targeted health interventions into health systems: a conceptual framework for analysis. Health Policy Plann. 2010;25(2):104–11. 10.1093/heapol/czp055 19917651

[58] Doel RE, Söderqvist T. Witnessing the witnesses: potentials and pitfalls of the witness seminar in the history of twentieth-century medicine. Technology, and Medicine: The Historiography of Contemporary Science; 2006. pp. 276–94.

[59] Nicholls EJ. The witness seminar: a research note. Qual Res. 2020;22(1):166–73. 10.1177/1468794120974153.

[60] Chakravarthi I, Hunter BM. Witness seminar on regulation of formal private healthcare providers in Maharashtra journey of Bombay nursing homes registration act and the clinical establishments act AND journey of Pre-Conception and Pre-Natal Diagnostics Techniques (Prohibition of Sex Selection) Act, 1994. 2019.

[61] Marathe S. SATHI Conducting the witness seminar. https://fmesinstitute.org/wp-content/uploads/2021/11/Witness-seminar-ppt.Shweta.pdf.

[62] Maas H. The method of the witness seminar. Hist Polit Econ. 2018;50(3):571–7.

[63] Campbell K, Orr E, Durepos P, Nguyen L, Li L, Whitmore C, et al. Reflexive thematic analysis for applied qualitative health research. Qualitative Rep. 2021;26(6):2011–28.

[64] Abimbola S, Negin J, Martiniuk AL, Jan S. Institutional analysis of health system governance. Health Policy Plann. 2017;32(9):1337–44. 10.1093/heapol/czx083 28981658

[65] Gyselink K, Das A, James A, Basu B, Putturaj M, Criel B et al. Sailing Below the Waves: A 20-year journey of Strengthening Local Health Systems in India. 2022. (Studies in Health Services Organisation & Policy). Report No.: 35. Available from: https://www.researchgate.net/publication/365361355_Sailing_Below_the_Waves_The_Story. Cited 2025 Apr 22

[66] Abimbola S, Negin J, Jan S, Martiniuk A. Towards people-centred health systems: a multi-level framework for analysing primary health care governance in low- and middle-income countries. Health Policy Plann. 2014;29(suppl 2):ii29–39. 10.1093/heapol/czu069 PMC4202919 25274638

[67] Pyone T, Smith H, Van Den Broek N. Frameworks to assess health systems governance: a systematic review. Health Policy Plann. 2017;32(5):710–22. 10.1093/heapol/czx007 PMC5406767 28334991

[68] Brinkerhoff DW. Accountability and health systems: toward conceptual clarity and policy relevance. Health Policy Plann. 2004;19(6):371–9. 10.1093/heapol/czh052 15459162

[69] Brinkerhoff DW, Bossert TJ. Health governance: principal-agent linkages and health system strengthening. Health Policy Plann. 2014;29(6):685–93. 10.1093/heapol/czs132 23411121

[70] Saltman R, van OC. Introduction. In: Implementing planned markets in health care: balancing social and economic responsibility. Buckingham: Open University; pp. 1–21.

[71] Jacob O, Neuman IB. Governance to governmentality: analyzing ngos, states, and power. Int Stud Quart. 2006;50(3):651–72.

[72] Janet N, Barnes M, Sullivan H, Knops A. Public participation and collaborative governance. J Social Policy. 2004;33(2):203–23.

[73] van Mr André R, Asta J, Pieter R, Piet F. B. Power and integrated health care: shifting from governance to governmentality. International journal of integrated care. 2016;16(3). Available from: https://www.ncbi.nlm.nih.gov/pmc/articles/PMC5350653/. 10.5334/ijic.2480 PMC5350653 28435425

[74] Roychowdhury P. VICTIMS TO SAVIORS: governmentality and the regendering of citizenship in India. Gend Soc. 2015;29(6):792–816.

[75] Pyysiäinen J, Halpin D, Guilfoyle A. Neoliberal governance and ‘responsibilization’ of agents: reassessing the mechanisms of responsibility-shift in neoliberal discursive environments. Distinktion: J Soc Theory. 2017;18(2):215–35.

[76] Bussu S, Bua A, Dean R, Smith G. Introduction embedding participatory governance. Crit Pol Stud. 2022;16(2):133–45.

[77] Gaitonde R, Muraleedharan VR, San Sebastian M, Hurtig AK. Accountability in the health system of Tamil nadu, india: exploring its multiple meanings. Health Res Policy Sys. 2019;17(1):44. 10.1186/s12961-019-0448-8 PMC6487063 31029173

[78] Shukla A, Kakde D, Scott K. Community monitoring of rural health services in Maharashtra. Economic Political Wkly. 2011;46(30):78–85.

[79] Stone D. Policy Paradox – The Art of polical decision making. New York: W. W. Norton & Company; 1997. p. 408.

[80] Molyneux S, Atela M, Angwenyi V, Goodman C. Community accountability at peripheral health facilities: a review of the empirical literature and development of a conceptual framework. Health Policy Plann. 2012;27(7):541–54. 10.1093/heapol/czr083 PMC3465752 22279082

[81] Kakoti M, Srivastava S, Chatterjee P, Mishra S, Nambiar D. Understanding the emergence of ‘communitization’ under india’s National rural health mission (NRHM): findings from two witness seminars. Glob Public Health. 2024;19(1):2306466. 39626045 10.1080/17441692.2024.2306466 PMC7617677

[82] Cornwall A, Lucas H, Pasteur K, Introduction. Accountability through participation: developing workable partnership models in the health sector. IDS Bull. 2000;31(1):1–13.

[83] Standing H. Understanding the ‘demand side’in service delivery. Definitions, frameworks and tools from the health sector. Issues Paper Private Sector, DFID Health Systems Resource Centre, London. 2004.

[84] World Bank. World development report 2004: making services work for poor people. Washington, DC: World Bank.

[85] Cleary SM, Molyneux S, Gilson L. Resources, attitudes and culture: an Understanding of the factors that influence the functioning of accountability mechanisms in primary health care settings. BMC Health Serv Res. 2013;13(1):320. 23953492 10.1186/1472-6963-13-320 PMC3844434

[86] Whitaker RG, Sperber N, Baumgartner M, Thiem A, Cragun D, Damschroder L, et al. Coincidence analysis: a new method for causal inference in implementation science. Implement Sci. 2020;15(1):108. 33308250 10.1186/s13012-020-01070-3 PMC7730775

[87] Vellakkal S, Gupta A, Khan Z, Stuckler D, Reeves A, Ebrahim S, et al. Has india’s National rural health mission reduced inequities in maternal health services? A pre-post repeated cross-sectional study. Health Policy Plann. 2017;32(1):79–90. 10.1093/heapol/czw100 PMC5886191 27515405

