# Supplementary material for: How communitization begets and endures sarkarikaran: a witnessed history of community action for health in India’s national rural health mission
Source: BMC Health Serv Res. 2025 Jul 8;25:940. doi: 10.1186/s12913-025-13058-0 (PMC12235939; doi:10.1186/s12913-025-13058-0)
Supplement: Supplementary file 1 — Supplementary Material 1. [file 12913_2025_13058_MOESM1_ESM.pdf]

Witness Seminar on Community Action for Health in India

‘Communitization’ and community-based accountability mechanisms under the National Rural Health Mission (NRHM)

First of Two Witness Seminars

Held online via Zoom on 13th November 2021

4

HeaL
Health equity action Lab

The George Institute for Global Health

Contents

**Instructions for Citation**	**3**
**Acronyms**	**3**
**Background and Purpose**	**4**
Community participation in health in India – key antecedents	4
The community-based accountability mechanisms under the National Rural Health Mission (NRHM)	4
References	5
**Witness Biographies**	**6**
**Proceedings of the Witness Seminar**	**9**
**Annexure**	**30**
In-depth Interview with Dr. Abhay Shukla	30
Responses from S. Jalaja	38

Edited by

Misimi Kakoti, Siddharth Srivastava, Shraddha Mishra, Gloria Benny, Hari Sankar, Devaki Nambiar

Publishing

First published in 2022 by
The George Institute for Global Health | INDIA
308, Third Floor, Elegance Tower
Plot No. 8, Jasola District Centre
New Delhi 110025 | India
T +91 11 4158 8091-93 | F +91 11 4158 8090
www.georgeinstitute.org.in

In support of an ongoing research collaboration with the Civil Society Engagement Mechanism (CSEM) for UHC2030 globally, the George Institute for Global Health India conducted Witness Seminars to document community action and social participation for health in India using internal funds.

All rights reserved. This material is made available for use for personal research and study. We give permission for the entire files to be downloaded to your computer for such personal use only. For reproduction or further distribution of all or part of the file (except as constitutes fair dealing), permission must be sought from the editors.

Download

This document is available to download as a free PDF and in other formats at: www.georgeinstitute.org/witness-seminar-reports

Acknowledgements

We would like to acknowledge Shweta Marathe, Indira Chakravarthi, Rakhal Gaitonde and Rajani Ved for their guidance at the early stages of this project. Ms. Rajani Tatineni provided support for the organisation of the Witness Seminar as well as preparatory meetings. Mr Jason Dass and Mr Alexander Baldock helped with graphics and design. We are also grateful to the team at Auxohub, led by Yashasvini and Vandana, for their careful work on the transcriptions.

Instructions for Citation

If you are using this document in your own writing, our preferred citation is:

Please cite the publication as a whole as: < Misimi Kakoti, Siddharth Srivastava, Shraddha Mishra, Gloria Benny, Hari Sankar, Devaki Nambiar (eds), 'Communitization' and community-based accountability mechanisms under the National Rural Health Mission (NRHM). Transcript of a Witness Seminar. (New Delhi: The George Institute for Global Health India, 2022)

References to direct quotations from this Witness Seminars should follow the format below:

[Witness name], in the 'Communitization' and community-based accountability mechanisms under the National Rural Health Mission (NRHM). Transcript of a Witness Seminar, held 13 November 2021, (New Delhi: The George Institute for Global Health India, 2022) www.georgeinstitute.org/witness-seminar-reports, [page number of reference]

Acronyms

**AGCA**	Advisory Group on Community Action
**ANM**	Auxiliary nurse midwife
**ARTH**	Action Research and Training for Health
**ASHA**	Accredited Social Health Activist
**CBM**	Community Based Monitoring
**CBMP**	Community Based Monitoring and Planning
**CEO**	Chief Executive Officer
**CHC**	Community Health Centres
**CHSJ**	Centre for Health and Social Justice
**CMHO**	Chief Medical Health Officer
**CNA**	Community Needs Assessment
**COVID**	Coronavirus disease
**CRM**	Common Review Mission
**CSO**	Civil Society Organization
**DFID**	Department for International Development
**DGHS**	Directorate General of Health Services
**FCRA**	Foreign Contribution Regulation Act
**IAS**	Indian Administrative Service
**ICPD**	International Conference on Population and Development
**IRS**	Internal Revenue Service
**JAA**	Jan Arogya Abhiyan
**JNU**	Jawaharlal Nehru University
**JSA**	Jan Swasthya Abhiyan
**MIS**	Management Information Systems
**MoHFW**	Ministry of Health and Family Welfare
**NGO**	Non-Government Organization
**NHM**	National Health Mission
**NHRC**	National Human Rights Commission
**NRHM**	National Rural Health Mission
**PFI**	Population Foundation of India
**PHC**	Primary Healthcare Center
**PIP**	Programme Implementation Plans
**PMO**	Prime Minister's Office
**RCH**	Reproductive Child Health
**RKS**	Rogi Kalyan Samiti
**RMRS**	Rajasthan Medical Relief Society
**RTI**	Right to Information
**SATHI**	Support for Advocacy and Training to Health Initiatives
**UN**	United Nations
**UNFPA**	United Nations Population Fund
**UPA**	United Progressive Alliance
**VHNSCs**	Village Health Sanitation and Nutrition Committee

Background and Purpose

Community participation in health in India[a] – key antecedents

Various global developments, including the Alma Ata declaration, the establishment of the People's Health Movement in 2000, and the International Conference on Population and Development (ICPD), have shaped the discourse around community participation in health. More broadly, the geopolitical context of Non-Aligned Movement, the New International Economic Order, and attempts to create an alternative paradigm for global development have centre-staged social participation, redistribution of power, and a rights-based approach for health.

Such has also been the case in India, where community participation in health and health reform precedes Independence. A range of individuals, institutions, and collectives set the stage for community action for health.[1] Building on these was the National Rural Health Mission (NRHM), launched in 2005 and widely lauded as a major health policy achievement, particularly for its emphasis on the role of community participation, and for resulting in major gains in India's advancement with the Millennium Development Goals.[2] NRHM created several institutional arrangements for community ownership and leadership in health. These included one of the world's largest community health worker programs, village- and facility-level committees with delegated financial powers, community monitoring, an action group tasked with supporting community action nationwide, and more.[3,4]

NRHM itself was designed to promote bureaucratic or programmatic decentralization in the health sector: decentralization of funds, functions, and functionaries to subnational government levels were part of the operational framework.[5] NRHM also recognized the importance of decentralization and district management of health programs, conceiving the district[b] as the core unit of planning, budgeting, and implementation.[6] In each state or union territory of India, however, existing contexts, path-dependent processes, and stakeholders were imbricated in the 'communitization' process in unique ways. We sought to understand these processes and history at the national and state levels using the Witness Seminar methodology.

Our methodological appendix is provided on our project landing page.

The community-based accountability mechanisms under the National Rural Health Mission (NRHM)[c]

Globally, since the 1990s, community participation has been increasingly linked to health systems accountability and governance. This is also reflected in NRHM's tenets, wherein it is recognised that the achievement of Health for All is possible "only when the community is sufficiently empowered to take leadership in health matters."[5] This policy intent was translated into interventions in the form of the Community Based Monitoring and Planning (CBMP) processes under NRHM.

The CBMP pilot was launched in 2007-08 by the Government of India and includes initiatives such as the Village Health Sanitation and Nutrition Committees (VHSNCs)[d] at the village level, the

a. This section is reproduced in each of five Witness Seminars that were carried out in 2021 with a focus on community participation in NRHM.

b. In the Indian administrative scenario, the nation is subdivided into states, and each state is further divided into districts. The districts are then made into smaller subdivisions of village and blocks in rural areas, and urban local bodies exist in urban areas.

c. This section is reproduced in each of two Witness Seminars that were carried out in 2021 with a national-level focus on community participation in NRHM.

d. Village Health Sanitation and Nutrition Committee (VHSNC) is a key institution introduced under NRHM to facilitate community participation in supporting, implementing, and monitoring health projects. It is formed at the level of the revenue village, and if the population of the revenue village is more than 4000, it can be formed at the level of a Ward Panchayat as it is in Kerala. From Government of India. (n.d.). Handbook for members of Village Health Nutrition and Sanitation Committee. Ministry of Health and Family Welfare.

Village Report Cards[e], and the Jan Samwads[f], among others. These were developed and implemented with involvement of NGOs, resource institutions and local communities; and the ASHA Mentoring Group, the Advisory Group on Community Action, and the Regional Resource Centres offering inputs to facilitate the process.[5] An evaluation of the CBMP pilot in 2008 reported improvements in health services from community-based monitoring in the states. Some major highlights from the evaluation include: VHSNCs' enhancement of knowledge on rights and entitlements in the community, the Jan Samwads' leading communities to demand better services, and an active engagement between the community and health departments.[6] CBMP–later renamed Community Action for Health (CAH)–was scaled up to cover more states from 2009 onwards.

Universal Health Coverage (UHC) has started occupying greater prominence in India's policy aspirations since 2010 and with the launch of the Ayushman Bharat programme in 2018. Community-based accountability and participatory governance of health systems are recognised as key elements for UHC.[7] Civil society has a crucial role in facilitating such accountability mechanisms in collaboration with the Government.[7] Since the National Health Policy of 2017 grants weightage to the role of the private not for profit sector in achieving UHC, the role of community-led accountability has become important to protect patients' interests and rights. Given this policy imperative, it is important to understand and determine how existing community accountability mechanisms–many innovated before but scaled up under NRHM–may be better leveraged to advance UHC commitments. Thus, we sought to deeply understand how CBMP structures have waxed and waned since their emergence in 2005. We organised a series of two Witness Seminars to document the provenance, features, achievements, challenges and lessons learnt from NRHM's CBMP/CAH processes.

References

1. Prachitha J, Dhume A, Subramanian S. India in Pursuit of Millennium Development Goals: Were the Targets Really Feasible? J Dev Soc. 2019 Mar 1;35(1):105–33.
2. Gaitonde R, San Sebastian M, Muraleedharan VR, Hurtig A-K. Community Action for Health in India's National Rural Health Mission: One policy, many paths. Soc Sci Med. 2017 Sep 1;188:82–90.
3. Seshadri SR, Parab S, Kotte S, Latha N, Subbiah K. Decentralization and decision space in the health sector: a case study from Karnataka, India. Health Policy Plan. 2016 Mar;31(2):171–81.
4. Raut M, Sekher TV. Decentralization of Health Care Systems: Findings from Odisha and Gujarat, India. J Health Manag. 2013 Jul 24;15:235–51.
5 Ministry of Health and Family Welfare. Framework of Implementation National Health Mission 2012-17 [Internet]. New Delhi: MoHFW; 2012 [cited 2020 Oct 22]. 59 p. Available from: https://nhm.gov.in/New_Updates_2018/NHM/NHM_Framework_for_Implementation__08-01-2014_.pdf
6 Singh S, Das A, Sharma S. Reviving Hopes, Realising rights- A report on the first phase of community monitiring of NRHM [Internet]. New Delhi: CHSJ; 2010. Available from: https://nrhmcommunityaction.org/wp-content/uploads/2017/06/A_report_on_the_First_phase_of_Community_Monitoring.pdf
7. Lahariya C, Roy B, Shukla A, Chatterjee M, Graeve HD, Jhalani M, et al. Community action for health in India: evolution, lessons learnt and ways forward to achieve universal health coverage. WHO South-East Asia J Public Health. 2020 Apr;9(1):82–91.

e. Some community action for health projects under NRHM use report cards to collect information from community members, such as for the monitoring of service quality at local health facilities. See: https://nrhmcommunityaction.org/wp-content/uploads/2016/11/Community_-Action_for_Health.pdf

f. Jan Samwads are public dialogues through which community members can share feedback on health services under the National Health Mission (NHM), which encompasses both NRHM and the National Urban Health Mission (NUHM). They owe their legacy to Jan Sunwais public hearings which were initially used for monitoring and grievance redressal purposes by civil society organisations. For more information, see: https://nrhmcommunityaction.org/bridging-the-digital-divide-connecting-communities-with-health-systems-through-virtual-jan-samwad/

Witness Biographies

Note: Biography information reflects the position of witnesses at the time of the seminar. Some designations and/or roles may have changed.

CHAIR:

Professor Rama V. Baru

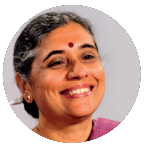

Professor Rama Baru is Professor at the Centre of Social Medicine and Community Health, Jawaharlal Nehru University, and an Honorary Fellow, Institute of Chinese Studies, Delhi, India. She is also an Honorary Professor at the India Studies Centre, Central China Normal University, Wuhan, China. Her major areas of research work include infectious diseases, comparative health systems, commercialisation of health services, and health inequalities. She is a member of the Ethics Committee at the All India Institute of Medical Sciences, the Technical Appraisal Committee for Health Technology Assessment, Department of Health Research at the Ministry of Health and Family Welfare (MoHFW), and the Scientific Advisory Group, Indian Council of Medical Research, New Delhi.

WITNESSES:

Dr. Abhijit Das

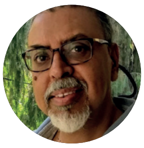

Dr Abhijit Das is the Founder and Managing Trustee of the Centre for Health and Social Justice, a policy research and advocacy institution on health and human rights, and gender equality. Dr Abhijit is a medical doctor with 35 years of experience in grassroots work, training, research and policy advocacy in the field of public health. He is the founder member of MenEngage, a global alliance of NGOs working with men and boys on gender equality, and Co-Convenor of COPASAH (Community of Practitioners on Accountability and Social Action in Health), a global health rights and social accountability network, and member of the Advisory Group on Community Action (AGCA). He is Clinical Associate Professor, Department of Global Health, University of Washington.

Dr. Abhay Shukla

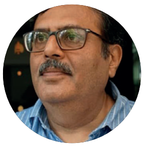

Dr Abhay Shukla, a public health physician, is Senior Programme Coordinator at SATHI-CEHAT. He is the national Co-convener of Jan Swasthya Abhiyan (People's Health Movement – India) and member of the Advisory Group on Community Action (AGCA). Dr. Abhay has edited and co-authored several books and reports on health care, including the Review of Health Care in India, Report on Health Inequities in Maharashtra, Nutritional Crisis in Maharashtra, and the Rights Approach to Health and Health Care. He has been involved in developing the framework of Community Based Monitoring and Planning of Health Services at the national level and is mentoring its implementation in Maharashtra. He is actively involved in campaigns for the regulation of the private medical sector, patients' rights, the Right to Health Care Campaign and initiatives for developing a system for Universal Health Care.

Dr. Sharad Iyengar

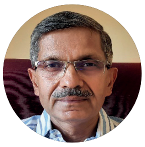

Dr. Sharad is a pediatrician and public health professional, involved in research and community interventions for RCH-FP at Postgraduate Institute of Medical Education & Research (Chandigarh, 1987-90) and UNFPA (Shimla, Delhi, 1990-98) in India. He is Co-founder, Action Research & Training for Health (ARTH), based in Rajasthan (1997 'til date), Co-Chair of Gender & Rights Advisory Panel (2000-07), and Member, Scientific & Technical Advisory Group (2008-13) of Human Reproduction Programme (HRP/RHR) of World Health Organization, Geneva. He has served on policy, guidance, and review groups of the National Health Mission and MoHFW, Government of India, and Government of Rajasthan. These include National Maternal Health Technical Resource Group, Core Committee to amend the MTP Act, State Population Commission and the Advisory Group on Community Action. Adjunct Professor, Sanford School of Public Policy, Duke University (2011-17)

Mr. A.R. Nanda

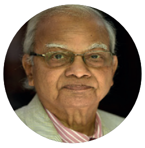

Mr. A.R. Nanda is former Executive Director (ED) of Population Foundation of India and was the ED when the Secretariat of the Advisory Group on Community Action (AGCA) was set up at PFI. He is a member of the AGCA. He has held many important positions in the Government, including Secretary, MoHFW, and Registrar General & Census Commissioner of India. Mr. Nanda has been a member/trustee of several organizations, including the Indian Association for Study of Population and the National Commission on Population. He has been an honorary fellow at the Indian College of Obstetricians and Gynaecologists. Mr. Nanda was the board chair at CHETNA for 15 years, and also at Freedom from Hunger India and Coalition for Food and Nutrition Security. He has served as an advisor on the International Advisory Committee for Population Programme of the David & Lucile Packard Foundation and the WHO Scientific and Technical Advisory Group of the Department of Reproductive Health and Research.

Dr. Rajani Ved

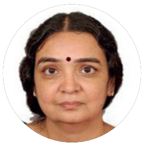

Dr. Rajani Ved has over 30 years of experience as a practitioner-researcher in the area of women and children's health and nutrition, and health systems.
As former ED of the National Health Systems Resource Centre (NHSRC) of the MoHFW, she led the institutionalisation of India's ASHA (Accredited Social Health Activist) program, and later the design and implementation support for India's flagship primary health reform, Ayushman Bharat (Health and Wellness Centres). Her expertise spans policy development, implementation research, health system strengthening, and design and evaluation of large-scale health programs. She is a visiting scientist at the Harvard T.H. Chan School of Public Health.

Dr. M.R. Rajagopal

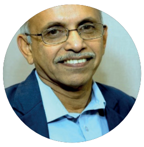

Dr. M.R. Rajagopal is an Indian palliative care physician. He is the founder chairperson of Pallium India, a palliative care NGO based in Kerala, India. He is a recipient of the Padma Shri Award (2018). Dr. Rajagopal's advocacy has contributed to the amendment of the Narcotic Drugs and Psychotropic Substances (NDPS) Act of India in 2014 and its implementation. He was also the prime mover in the creation of the National Program for Palliative Care (NPPC) by the MoHFW.

Dr. Tarun Seem

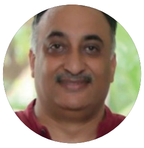

Dr. Tarun Seem is a medical graduate from New Delhi. He served with the Central Himalayan Rural Action Group (CHIRAG), an NGO at Uttarakhand, before joining the Indian Revenue Service (IRS) in 1992. He is presently serving as a Commissioner of Income Tax at New Delhi. Dr. Seem has served as Director in Ministry of Health, Government of India, during the formative years of the National Rural Health Mission. He has been Head of the Health Systems Support Unit at Public Health Foundation of India, where he steered the National Initiative on Allied Health Services. Dr. Seem has also served the Government of Delhi as Director Health Services and as Secretary (Health & Family Welfare). He has mentored the program for free, assured access to medicines at Delhi hospitals and the Mohalla Clinics project, both widely recognized as pathbreaking public service initiatives in the health sector. Dr. Seem has been trained in professional courses at IIM Ahmedabad, Chulalongkorn University (Bangkok), Duke University (North Carolina), Sciences Po, (Paris), Hertie School of Governance (Berlin) and University of Indonesia (Bali). He has a Diploma in Public Policy and Management from MDI, Gurgaon where he was awarded the School of Public Policy and Governance, Gold medal. Dr. Seem completed his LLB from Lucknow University. He is presently enrolled for PhD at IIT, Delhi in the stream of Information Systems for Primary Health Care.

Ms. Poonam Muttreja

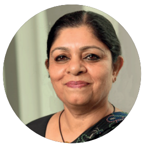

Poonam Muttreja, ED of the Population Foundation of India, has for over 40 years been a strong advocate for women's health, reproductive and sexual rights, and rural livelihoods. She has co-conceived the popular transmedia initiative, Main Kuch Bhi Kar Sakti Hoon—I, A Woman, Can Achieve Anything. Before joining PFI, she served as the India Country Director of the John D. and Catherine T. MacArthur Foundation for 15 years, and has also co-founded and led the Ashoka Foundation, Dastkar, and the Society for Rural, Urban and Tribal Initiative (SRUTI). An alumna of Delhi University and Harvard University's John F. Kennedy School of Government, Poonam serves on the governing council of several non-governmental organisations and is a regular commentator in India and globally for television and the print media.

Mr. Bijit Roy

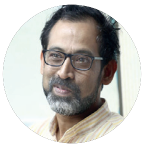

Bijit Roy has designed, led and delivered large and complex public health and nutrition programmes. Over the last twelve years, Bijit has led the institutionalization and scaling up of community action and accountability processes under the National Health Mission (NHM) on behalf of the MoHFW across 25 states in India. He has represented in various national level advisory/ expert committees. He is a Fellow with the Lancet Citizen's Commission on Reimagining India's Health Systems. Bijit has an overall experience of twenty-three years, working with the Population Foundation of India since 2007 and earlier with CARE from 1998 to 2006.

Proceedings of the Witness Seminar

Proceedings start

Devaki Nambiar: Welcome, everyone. So, I will keep trying [name of observer], but I think in the interest of time, we probably should get started. So, I will hand over to Misimi who has been emailing so frantically about this to get this evening started, followed by our Chair, Professor Baru. So, over to you, Misimi.

Misimi Kakoti: Thanks, Devaki. A very good morning to all the participants present with us here today, on behalf of The George Institute for Global Health. Thank you all for taking out time for this, and we are extremely glad to have you all together for this session. Before we begin the seminar, I would like to quickly go through a round of welcoming all the witnesses present with us here today. [an observer] is not here. I will start with Dr. Rajani Ved. Ma'am is the former Executive Director at the National Health System Resource Centre[1]. Then we have Shri A. R. Nanda here, who is a retired IAS officer[2], former Executive Director of the Population Foundation of India. We have Dr. Abhijit Das, who is the former Director of the Centre for Health and Social Justice. Then, we have with us Dr. Abhay Shukla, who is the Senior Program Coordinator at SATHI-CEHAT[3]. We have Dr. M. R. Rajagopal, who is the Chairperson at Pallium India, and then we have Ms. Poonam Muttreja, who is the current Executive Director at the Population Foundation of India. We have Dr. Tarun Seem, who is an IRS officer. Then we have Bijit Roy, who is the Associate Director at the Population Foundation of India, and then we have Dr. Sharad Iyengar, the Chief Executive at the ARTH[4]—Action Research and Training for Health. The Chairperson for the session today is Professor Rama V. Baru. She is from the Centre for Social Medicine and Community Health, JNU[5]. Going over the theme of the Witness Seminars—that is ‘Community Participation in Health in India’—,we are focusing on the community-based accountability mechanisms under the National Rural Health Mission. The provenance, contexts, actors, challenges of the process. There are two key modalities of the method of the Witness Seminars that I would like to reiterate that you might have come across in the participant information sheet.

Misimi Kakoti: Yes, Sir [A.R. Nanda]?

A.R. Nanda: Yes, I have joined, signed the consent form.

Misimi Kakoti: Thank you Sir, thank you so much.

Misimi Kakoti: I will continue. So, the Seminar is recorded, and the audio recording will be transcribed. The transcription will be shared with all the witnesses for them to review, comment or edit their portions. And after they have approved the transcription, we finalise it in the form of a detailed annotated report and send it to all the witnesses for a final review. And the annotated transcript will be the main output of the seminar. It will not remain anonymous, and all the witnesses will be attributed and acknowledged for their responses and contributions. We intend to listen to you earnestly and document your side of the story and your narratives around a very significant history of India's policymaking. We will refrain ourselves from

1 The National Health Systems Resource Centre (NHSRC), established in 2007, supports the development of policy and strategy in “the provision and mobilization of technical assistance for health programmes to the states, and in capacity strengthening for the Ministry of Health [and Family Welfare]” in India. See: https://nhsrcindia.org

2 IAS officers work at the national level within India to enforce the law, collect and administer revenue, and shape policies, among others. See: https://cseplus.nic.in/Home/DisplayPDF?streamId=PCsUnEplvZihdzEe8FEMf/ozdm2Z1SV9EtWCRovKRC3lakWb5Az6zBHuA5aOBE/F63T7xmhv7oprudau8IxE7qwEbpfY7FdzRY38CgzX0Ow3cxPusYL1aWOaLZjLhzSg

3 The SATHI team, launched in October 1998, was originally a part of CEHAT. On 1 April 2005, SATHI was transformed into an action centre of Anusandhan Trust. Its headquarters are located in Pune, Maharashtra, India. It addresses health rights-related issues through civil society organization (CSO) partnerships. SATHI also facilitates local-, district-, state-, and national-level advocacy. See: https://sathicehat.org

4 Action Research and Training for Health (ARTH) is a not-for-profit organization whose work is centred around women's reproductive health. ARTH aims to “help communities access and manage health care according to their needs and capacity, by using research and training initiatives.” See: https://www.arth.in

5 Jawaharlal Nehru University (JNU), established in 1967, is a major postgraduate research university within New Delhi, India. Named after India's first Prime Minister, Jawaharlal Nehru, JNU is funded by the central government.

any difference of opinion that may arise during discussions. We hope you had a chance to go through the agenda and the key questions that we had shared with you. To ensure that everyone has an opportunity to cover their points, we request the witnesses to keep their points to around 7-8 minutes. Before handing the session over to Rama Ma'am, I think I would request consent individually from the participants who could not share their consent and I will just take a minute or so for that, I think. If you could unmute yourself and state your consent, please.

Sir A.R. Nanda has mentioned that he would share his consent after the meeting, and I would request Tarun sir to kindly unmute yourself and state your consent please, thank you.

Tarun Seem: I will do that, do you say, I do or something.

Misimi Kakoti: Sir, you just have to say, you consent for participating.

Tarun Seem: I consent for participating.

Misimi Kakoti: Thank you Sir, thank you so much to all of you. With this, I would hand over to our Chair. Rama Ma'am, over to you.

Rama Baru: Thank you very much, thank you to Devaki for persuading me to chair this session. I am delighted because I am seeing a lot of old friends come together, and I think we would all agree that the launch of NRHM[6] and what went behind it in reshaping its agenda was a very important point in the history of health policymaking in this country. It was also very important because civil society organisations really came together despite different ideological moorings and really defined the moment for some form of reform (in quotes as I put it), "to align ourselves to Alma-Ata Declaration and health for all[7]."

I think it was really a moment—a political moment—that provided that space due to the coalition politics of the first UPA government[8], and the presence of the Left and other democratic formations, which opened up spaces for this kind of a dialogue. I think the significance of especially community participation also provided the space for many NGOs that have been working for many years in building community processes and ensuring community participation, to actually bring their ideas on how one could mainstream it into program like NRHM, and subsequently the institutionalisation, visioning the need for trainings and for monitoring, etc. So, I am not going to elaborate any further. Each of you has been witness to this process. We all have made sense of it in different ways, and we also see its continuities and discontinuities as we speak in 2021. So, with those very few opening remarks, I would request Abhijit Das to make his preliminary remarks on the emergence of the NRHM as he has his perceptions and experiences. Thank you very much.

Abhijit Das: Thank you Rama, and a very good morning to all of you. As Rama said, it is a fabulous opportunity to meet up with old friends and colleagues. I think this is an excellent opportunity to go down memory lane. I will stick to the agenda that has been sort of suggested for me—that is, the emergence. What I would also like to start with is a caveat that this is from my perspective; my testimony as a witness participant. All of you have been different players in the same story. So, you have your story which will be different, and I would like to apologise in advance if there are places in which there are some disagreements in the way you see it vis-à-vis what I have seen and experienced. Because these are common spaces and we come with our individual and unique perceptions and experiences, I will begin with that caveat.

6 The National Rural Health Mission (NRHM) is a centrally sponsored scheme of the Government of India launched in 2005 to provide affordable, equitable, and quality health care to the rural population. The thrust of the scheme has been on setting up a community-owned and decentralized healthcare delivery system with intersectoral convergence to address determinants of health such as water, sanitation, education, nutrition, and gender equality. Since 2013, it has been integrated under the overarching National Health Mission (NHM) alongside the National Urban Health Mission (NUHM). See Government of India (n.d.). National Rural Health Mission: Framework for Implementation (2005-12). Ministry of Health & Family Welfare. https://nhm.gov.in/WriteReadData/l892s/nrhm-framework-latest.pdf

7 The Alma-Ata Declaration was adopted at the International Conference on Primary Health Care (PHC), Almaty, Kazakhstan, 6–12 September 1978. It was the first international declaration underlining the importance of primary health care. See: https://www.who.int/teams/social-determinants-of-health/declaration-of-alma-ata

8 The United Progressive Alliance (UPA) is a coalition of political parties in India formed after the 2004 general election. The largest party in the UPA alliance is the Indian National Congress. See: https://journals.sagepub.com/doi/10.1177/0974928416654367?icid=int.sj-abstract.similar-articles.1

I will start with the emergence as I see it. The story for me starts much earlier, actually after Cairo[9] and the HealthWatch[10] experience, [for] which Ms. Poonam and Mr. Nanda were very much there. I think at least for me, as a very young public health professional at that time, that started a conversation about the emergence of engaging with the public system. We started this network called the HealthWatch, and the HealthWatch was doing monitoring of the target-free approach. So, the idea of the civil society monitoring public programs sort of got embedded in my own practice, so that is a particular strand that I think is very important in the emergence [of] the CNA (Community Needs Assessment), which Mr Nanda was very much a part of. Engaging in the Community Needs Assessment process, using participatory processes to understand family planning needs, which was different from the devising of methods statistically which had started in Tamil Nadu – and I met Ram yesterday. So, that is one big difference. Another thing—I think as Rama mentioned—was the People’s Health Assembly. The People’s Health Assembly brought together the other strand of civil society which was committed to the Alma Ata agenda. The People’s Health Assembly created the Jan Swasthya Abhiyan[11].

On one side, I see myself being influenced by the HealthWatch process and by the Jan Swasthya Abhiyan People’s Health Assembly process. Both these contributed to the emerging confidence of the civil society to engage with public systems. Having said that, there were two other things that were important from my perspective. One was the JSA-NHRC (National Human Rights Commission[12] public hearing process) that Abhay was very closely involved in, and that’s where Abhay and I started closely working together. And we started getting involved in accountability of the public system and JSA did a fabulous piece of work there. So, that became a very practical example. On the other hand, as an individual, I was a part of what UNFPA[13] was trying to do in terms of doing reproductive health community engagement, gender sensitisation, and working on women’s rights and accountability through State Human Rights Commission in various states like Rajasthan, Orissa, and others in which I started working with Narendra and Renu and others. What I am trying to say is, the idea of working collaboratively emerged, and I think another thing that emerged from this process—and [an observer] was there at DFID[14] and I had known Tarun from much earlier... Tarun and I were colleagues—is that there was a sense of reciprocal respect between people in bureaucracy who wanted change and people like us in civil society who had sort of gotten the space and were also engaging in respectful relations with the public system. I think this was extremely important and it predates the formation of NRHM.

9 The United Nations coordinated an International Conference on Population and Development in Cairo, Egypt, from 5 September 1994 to 13 September 1994. Its resulting Programme of Action is the steering document for the United Nations Population Fund. See: https://www.unfpa.org/news/explainer-what-icpd-and-why-does-it-matter

10 HealthWatch was formed in December 1994 and comprised a group of Non-Governmental Organisations, academics, activists and concerned citizens involved with the pre-Cairo consultative process. The remit of this group was to provide criticism as part of ongoing dialogues with government stakeholders, to offer alternatives for implementation of sexual and reproductive health and rights programs and to help develop monitoring indicators. See: https://www.jstor.org/stable/2991871

11 “The Jan Swasthya Abhiyan (JSA) was formed in 2001, with the coming together of 18 national networks that had organised activities across the country in 2000, in the lead up to the First Global Peoples Health Assembly, in Dhaka, in December 2000. The JSA forms the Indian regional circle of the global People’s Health Movement (PHM). At present it is a major national platform that co-ordinates activities and actions on health and health care across the country.” See: http://phmindia.org/about-us/

12 The National Human Rights Commission (NHRC) of India, formed on 12 October 1993, is the autonomous public body “for the promotion and protection of human rights” in India. See: https://nhrc.nic.in/about-us/about-the-Organisation

13 UNFPA is the United Nations’ sexual and reproductive health agency. See: https://www.unfpa.org

14 The Department for International Development (DFID) of the British government was tasked with contributing to global poverty elimination efforts. DFID India, whose headquarters were located in New Delhi, was one of multiple DFID offices globally. It worked toward “strengthening the capacity of government to develop and implement pro-poor policies”, “promoting increased investment in education, health and clean water”, and more. On 2 September 2020, DFID and the Foreign and Commonwealth Office amalgamated to form the Foreign, Commonwealth & Development Office (FCDO) of the UK Government. See: https://ngosindia.com/department-for-international-development-dfid/ and https://assets.publishing.service.gov.uk/government/uploads/system/uploads/attachment_data/file/913355/India-Profile.pdf

During the RCH-2-[15] development process, there was a conversation about including social accountability. The DFID people got in touch with me. I said I won't do it alone; we will do it together. Because before that, we all worked together in the JSA platform to do social accountability and there was a UNFPA-DFID meeting in the UN office, and I think Abhay and Narendra and all others had attended. I forget the DFID gentleman's name, but Venkat and Ena Singh from UNFPA were there. This is before the NRHM. This is one process which leads up to civil society consolidating its thoughts and having experience and confidence to work with public systems. I think there is a reciprocal relationship happening.

Part two ([that is], NRHM), I think JSA had got the confidence because of its engagement with political processes before the election, and there was a space, which I will leave for friends who are more embedded in JSA to answer and to deal with that. What happened was that there was a meeting in Samrat Hotel and the NRHM conceptualisation was shared. I think Rama was also there in that meeting. One thing was clear: there was lack of clarity in the Ministry of Health and Family Welfare as to what exactly NRHM was going to achieve. In some ways, it was the articulation of a targeted program for 150 low performance districts, etc. So, there was a family planning push from there, and at the same time—I think from the PMO [Prime Minister's Office]—, there was a push to do a more of a community health worker-based public health program. So, there was a lack of agreement within the Ministry. At lunch, a lot of us [who were] part of the JSA sat down together and said: listen, we are discussing in September and the plan of rolling it out [is] in November. Mr. Ravi, who was hosting the JSA global—Ravi Narayan with CHC [Community Health Cell][16]—, I think he worked with Dr. Antia to arrange for a meeting with the Prime Minister, and the NRHM planning process was a bit delayed, and eight task groups[17] were set up. By that time, Mr. Sinha moved to the Ministry from DFID if I am not wrong, and the NRHM's planning started happening in a much more deliberate manner. Community participation became one task group. I think Abhay was a member of that task group. At the same time, CHSJ[18] was set up, and Mr. Nanda was very much part of the process, helping me set up CHSJ. One of the agendas was to contribute as well as keep a critical eye on how the implementation was taking place. I remember we did it with PFI[19]—Mr. Nanda was the Executive Director of PFI. We conducted an assessment on rollout of [the] NRHM public health program in one year. AGCA[20] [the Advisory Group on Community Action] was just in the starting stage. AGCA was initially getting bogged down by discussing NGO projects, and one of the things that Abhay and I pushed for is that, instead of AGCA

15 The Reproductive and Child Health (RCH) Programme was launched throughout India on 15 October 1997. The first phase of RCH worked toward "achieving a status in which women will be able to regulate their fertility, women will be able to go through their pregnancy and child birth safely, the outcome of pregnancies will be successful and will lead to survival and well being of the mother and the child." Mr. Abhijit Das refers to the development of RCH-2, which aimed to reduce India's total fertility rate, infant mortality rate, and maternal mortality rate. See: https://www.nhp.gov.in/reproductive-maternal-newborn-child-and-adolescent-health_pg and https://nhm.gov.in/index1.php?lang=1&level=1&sublinkid=794&lid=168

16 The Community Health Cell (CHC) "is a functional unit of Society for Community Health Awareness, Research and Action (SOCHARA)", collaborating with non-governmental and governmental organizations, campaign groups, and people's movements "to make them part of this 'Health for All' movement." See: https://www.sochara.org/clusters/Community_Health_Cell_CHC_Bengaluru

17 To ensure operationalisation of various activities under NRHM, the Government of India constituted eight task force groups. Each group was tasked with various activities of the NRHM, ranging from reviewing goals, strategies, timeframes, and outcomes, to technical assistance, providing training support to ASHAs, fostering community engagement, exploration of health financing mechanisms, reviewing public private partnerships, building institutional linkages with the government, etc. See: http://nhm.gov.in/images/pdf/communitization/task-group-reports/tasks-for-task-groups.pdf

18 The Centre for Health and Social Justice (CHSJ), established in 2005 as a Public Charitable Trust, helps "promote social justice with specific reference to the fields of health, human development, gender equality, and human rights". See: https://chsj.org/who-are-we/#foundingStory

19 Population Foundation of India (PFI), a nationwide non-governmental organisation (NGO), "advocates for the effective formulation and implementation of gender-sensitive population, health and development strategies and policies." See: https://populationfoundation.in/who-we-are/#OurPeople3

20 The Advisory Group on Community Action (AGCA), whose secretariat is hosted by Population Foundation of India, was amalgamated in 2005 by Ministry of Health and Family Welfare (MoHFW). The AGCA advises the community action for health activities under the National Rural Health Mission (NRHM). It consists of "eminent public health professionals associated with major NGOs." See: https://nrhmcommunityaction.org/agca/

being a monitor of which grant goes to an NGO, which will lead to some members within AGCA applying for the grants having conflict of interest, why don't we design more deliberate accountability processes? Mrs. Jalaja agreed that AGCA could start doing a design of how it should be done, and I think that is how AGCA started designing the community monitoring project. Once we designed it, Mrs. Jalaja said, "Why don't you pilot it?" Mr. Nanda was hosting the AGCA Secretariat, he asked me if CHSJ would be interested in doing the technical part and I agreed. That is how, in many ways, from my perspective, the pilot program of community monitoring[21] started. I will end there, thank you so much.

Rama Baru: Thank you so much and thank you for keeping it to the time. Now, may I request Abhay to come in?

Abhay Shukla: Thanks a lot to The George Institute and the entire team and Rama for giving me this opportunity. As I messaged Devaki today, to retrace a very complex, contentious, and exciting process, which has unfolded at multiple levels over the last 15 years, and to cover it in eight minutes is impossible. Even in a summary form. So, I will be telegraphic, and you will have to decode what I am saying and probably these points can be elaborated [upon] later on in a one-to-one discussion with the research team. I will talk about the emergence and a little bit further about institutionalisation. So, the story of community accountability under NHM is a complex story of contention between two kinds of forces related to communitization. It is a story of the social will for communitization from below, as struggling with the waxing, waning, and now nearly disappearing political will for communitization from above. It may be a provocative statement, but I am willing to defend this.

When it started, CBMP[21] was co-created by reformers within the government and activists in civil society. In contrast to external donor-driven projects (which are top heavy but will lack roots on the ground), or purely NGO initiatives (which are strong on the ground but lack leverages within the system), or government sort of formal committees like VHNSCs[22] (which are often empty signifiers), CBMP manages to connect health systems and communities together in a collaborative process which was quite unique, at least during the period when there was political and social will from both sides. "Do haath se taali bajti hai, ek haath se nahi," ["You cannot clap with one hand alone; you need two hands"] you know, and I will come to that later. I would like to emphasise what Abhijit has already very well pointed out: without the influence of the health movement in the broader sense and actions by rights-based Civil Society Organisations in a more specific sense, CBMP would not have emerged. I think this point needs to be underscored. Of course, there was a receptive environment within the government which very much allowed and encouraged this process. So, Abhijit has already mentioned about public hearings organised by the Jan Swasthya Abhiyan with the National Human Rights Commission [NHRC][12]. I would like to add that Mrs. Jalaja was a joint secretary in NHRC when we organised public hearings, and then she was in the Ministry when the NRHM was launched. So, there was a continuity between the health rights outside the system or margins of the system and in bringing health rights into the system. You know, at least an attempt was made through the NRHM.

As Abhijit has mentioned, [we were] this small group of health activists which included Abhijit, Narendra Gupta and Renu Khanna and myself. We interacted and Amarjeet Sinha ji had set up a task force on district health planning where Dr. Sundararaman and I were members, and that gave us the opportunity to shape the CBMP framework. Now, I will shift to actual design and institutionalisation in Maharashtra. The CBMP experience in Maharashtra was collaborative social accountability. Community monitoring itself, to

21 The Community Based Monitoring and Planning (CBMP) process was introduced in June 2007, as part of the NRHM, to facilitate community-led monitoring of health programmes and services to ensure that people's health needs and rights are fulfilled. CBMP's pilot phase was guided by the Advisory Group on Community Action (AGCA) and was implemented in nine Indian states between 2007 and 2009. See: https://nrhmcommunityaction.org/ and https://nrhmcommunityaction.org/pilot-phase/

22 Village Health Sanitation and Nutrition Committee (VHSNC) is a key institution introduced under NRHM to facilitate community participation in supporting, implementing, and monitoring health projects. It is formed at the level of the revenue village, and if the population of the revenue village is more than 4000, it can be formed at the level of a Ward Panchayat as it is in Kerala. From Government of India. (n.d.). Handbook for members of Village Health Nutrition and Sanitation Committee. Ministry of Health and Family Welfare.

begin with, was a deliberately fuzzy concept, and, later on, the idea of community action for health was an even fuzzier concept. Fuzzy concepts have some advantages— they allow some very diverse actors with different perspectives to work together. We all say we are all here for community action for health, but each actor understands [it] quite differently. Anyway, in Maharashtra at least, the CBMP process had to skilfully combine CSO advocacy from above at state level to open up participatory spaces, and very active community mobilisation from below to occupy and activate these spaces. This is what we actually did in a very concerted way, and this required autonomous civil society facilitation at multiple levels (from grassroot level to state level, and also supported by AGCA at the national level). That is what enabled us to work with the government with a multilevel layer sandwich strategy kind of approach. You must be aware of that sandwich strategy idea, like a sandwich with multiple layers, with each level there were actors appealing to the higher level and also opening up spaces at the lower level. This enabled us to convert what are called 'vacant signifiers', like the Village Health and Sanitation Committee, which is a very great idea but on ground, it was almost empty of content. We enabled community-based actors to occupy these vacant signifier spaces and activate them, start using them and also Rogi Kalyan Samitis[23]. I can spell this out later. You know, at the same time, a very flexible approach was required, and we had to constantly innovate during this process. Throughout 12-13 years, the policy environment was also changing, the community expectations were changing, and the health system responses were also changing.

Actually, what happened is that... in this course of 15 years in Maharashtra, we had completed an entire cycle of change. In 2004, before NRHM, we had six Jan Sunwais[24], probably the first Jan Sunwais in Maharashtra organised by the JSA. Then, with NRHM opening up and CBMP being institutionalised, we had 600 Jan Sunwais in Maharashtra as a part of CBMP. Between 2008-2015, we were able to move from six to 600. After 2015, we have seen a progressive constriction. I would not say progressive... we have seen a major constriction of the CBMP process with the whittling down of financial support [and] waning political support. Ultimately now, we had converted what were hegemonized spaces into participatory spaces. Now, we are seeing those participatory spaces being hegemonized and constricted by the state. I will not elaborate on this; I think you can understand what I am talking about. Rights-based community action for health has been a constant throughout this process. It is like flowing water which will continue to flow even if there are obstacles, whereas the state support for the community accountability has been variable and depends on the wider political climate. As far as the social accountability processes are concerned, communities are primary and state is secondary, and it is not the other way around and I would like to emphasise this point. Community action will continue whether there are spaces or not, but the state's ability to digest and appetite to tolerate community action might be quite variable. So, that is why we can talk about contentious spaces, unequal forces, and persistent voices in the whole process. Coming to what we did in Maharashtra... Maharashtra has been a positive outlier for CBMP, I would say because we managed to maintain many of the critical elements in the CBMP process from the pilot phase into the post-pilot phase and even beyond that, and [have been able] to continuously expand it while ensuring that Civil Society Organisations remained in co-leadership roles at all

23 Rogi Kalyan Samitis (RKSs), also known as Hospital Management Committees and established under the National Rural Health Mission (NRHM), act as fora to enhance the operations and service delivery in public health facilities, and to advance community participation and accountability from health services. From Government of India. (n.d.). Guidelines for Rogi Kalyan Samitis in Public Health Facilities. Ministry of Health and Family Welfare. Retrieved from https://nhm.gov.in/New_Updates_2018/communization/RKS/Guidelines_for_Rogi_Kalyan_Samities_in_Public_Health_Facilities.pdf

24 Jan Sunwai is a Hindi phrase which means 'Public Hearing'. It is like an informal court composed of local people as its judges, wanting accountability. The practice of Jan Sunwai, an instrument of a social audit, can be dated back to the pre-Independence era, and it has been widely appreciated as a democratic means of bolstering participation. Jan Sunwai is a democratic way to familiarise local people with government policies and the activities of the public authorities so that they can understand what the government is doing towards the development of their communities. NRHM recognizes the Jan Sunwai as a community advocacy and monitoring mechanism. See: https://journals.sagepub.com/doi/abs/10.1177/2321023018797537 and https://nhm.gov.in/WriteReadData/l892s/nrhm-framework-latest.pdf

levels (from the state level to the village grassroots level).

Through this process we did at least three things. We developed CBMP as a participatory collaborative problem-solving network and promoted transformations at three levels. One, changes within communities in terms of awareness and mobilisation. Two, changes in interaction between communities and the health system in terms of various participatory forums. Three, changing the functioning of the health system in terms of access and in some cases, quality of care. This process also challenged us to find ways to sustain our autonomy while depending on the government for our funding and official standing, which is a very complex process, and I am not going into the details.

Rama Baru: You have a minute.

Abhay Shukla: Okay. So, as I call it, the 'red queen' dilemma[25]: we have to keep running as fast as we can just to remain in the same place. The CBMP process encouraged shifts in power at the micro level and we have seen those shifts in power across different places. Today, with the collapse of support for rights-based civil society organisation at the national level, I am being frank with all of you: our long march of the last 14 years to institutionalise community health rights within the health system has [been] met with a stone wall. Now, we are forced to step outside the system to continue our struggle for community health rights and as a part of Jan Arogya Abhiyan campaign[26] in Maharashtra. Now, we have launched a new phase of the Right to Health Care campaign. Institutionalisation has been a double-edged process for us. It reduced our sharpness but expanded our reach, but now we are back to another phase of the struggle. [This is] mostly outside the system. Without countervailing power in some form, there is no genuine community accountability. This is the crux. However, this countervailing power is often not digested by the government. They are often treated as foreign bodies within the system, either to be absorbed or rejected from the system. But we must remain as a countervailing power, working with the system and also constantly challenging the system. Otherwise, [the] government's control on community action can be like the touch of Midas[27] or embrace of Dhritarashtra[28], which can completely constrict the entire process. We have seen multiple examples of that. If the state starts shaping actions by communities, rather than community shaping action by the state, then the spirit of communitization is finished. That is sarkarikaran [governmentalisation], not communitization. I will conclude here by saying that the COVID epidemic has shown us how co-production of health needs to be the way forward for the next phase of community action for health. Thank you.

Rama Baru: Thank you, Abhay. May I request Sharad Iyengar to make his remarks?

Sharad Iyengar: Thank you Rama, and thanks to the George Institute for providing me with this opportunity to learn and share some experiences. I am very glad that I am following them, but in contrast to what Abhijit and Abhay have spoken about, I must confess that I have not been involved in [the] formulation of community action work in the initial stages or the discussion with people in the Ministry. Perhaps part of this is a feature of living and working outside—in one of the states

25 The Red Queen hypothesis, proposed by Leigh Van Valent in 1973, originates from evolutionary biology. It posits that species must continuously adapt, evolve, and proliferate to survive while pitted against ever-evolving competing species. See: https://www.ascm.org/ascm-insights/the-red-queen-hypothesis/

26 Jan Arogya Abhiyan is the Maharashtra chapter of People's Health Movement India (i.e., Jan Swasthya Abhiyan). Jan Arogya Abhiyan has been involved in a series of campaign actions in Maharashtra to demand the Right to healthcare, including during the COVID pandemic. see: www.janarogya.org

27 Midas is the name of one of at least three members of the royal house of Phrygia. The most famous King Midas is popularly remembered in Greek mythology for his ability to turn everything he touched into gold, which became a curse because his own child turned to gold because of his touch. This came to be called the golden touch, or the Midas touch. See: https://www.greeka.com/greece-myths/king-midas/#:~:text=The%20story%20of%20King%20Midas,a%20blessing%2C%20but%20a%20curse

28 Dhritarashtra was "featured heavily in the Hindu epic Mahabharata as the interim King of the Kuru Kingdom with its capital at Hastinapur."The embrace of Dhritarashtra refers to a curse placed on the King wherein anyone he would embrace would be destroyed. It refers to the danger in embracing someone or something without fully examining the costs that may be involved. See: https://thewire.in/politics/dhritirashtra-embrace-modi-embrace

and not in the national capital, not even by way of visiting the national capital regularly. What I would do is, having been invited on and having been a member of the AGCA from its very early days, I would look at what were the design elements in light of the context of the states. Like Abhijit stated his biases, my perspective comes from living in Rajasthan and northern states. I feel that there are important differences in the extent to which health systems are able to address the issue. And here, we are talking of the public health system or the department of Medical Health and Family Welfare, as it is termed in the state of Rajasthan, and how they relate to communitization and community action. More clearly, the National Rural [Health Mission]—and renamed as National Health Mission [NHM]—has been seen as this very large bag of money with new ideas, innovations, and flexibility in order to achieve better health outcomes. There are various points or locations in a state, and by extension in a district or sub-district level, where you have a health department and a National Health Mission or a mix of the two. NHM is seen as a project within its parent health department. It has funds, it has purpose, [it is] more of time-bound action, and it can get things done. However, it rests essentially on the parent platform that is the health agency. Rajasthan has a different form of Panchayat Raj system as compared to Maharashtra; and it is my personal observation that in states in which Panchayat Raj system [has] evolved more, there is more devolution of power, especially as applied to health. There is perhaps greater space for community processes to come into the work of both the Health Mission and the health department. In Maharashtra, the District Health Officer says he/she is the district health officer of the Zila Parishad[29]; of a particular district. In Rajasthan, a Chief Medical Health Officer, says that "I am CMHO[30] [Chief Medical Health Officer] of the Department of Medical Health and Family Welfare, Government of Rajasthan". So, there is that difference, difference in openness, perhaps a little less openness to look at community elements and accountability. I feel that one of the processes that supported the acceptance of communitization was the coming of the Right to Information Act[31] (RTI). It essentially signalled to the people down the rank and file of government implementing agencies that openness and transparency is important… Not just important, it is the right of the people, and they can go around demanding it, and you are supposed to share. Another matter is of how well it is done or what are the inefficiencies and unwillingness in specific instances. But the openness is about sharing and letting the community know what we are doing and telling them all about it.

At the same time, in a society that is quite hierarchical, there is this continuing relentless way of looking at the community as being far less informed about health issues. There is information asymmetry and health issues have been very technically framed. Therefore, the feeling among health personnel [is]: what does the community—what do less educated, illiterate people—understand about healthcare, while they have all these unreasonable expectations which cannot be delivered upon? Whereas there is a different way of doing public health. This is the kind of sentiment that has guided the communitization process and the pilot in the early stages. Which is that there has been a very strong scepticism about, "What does the community know, how can you get involved and who are they to tell us what to do?" There has been a lack of devolution of powers, political powers, in other fora which would be conducive. The RTI has been a positive factor. The other positive factor has been the formation of

29 The Zila Panchayat or District Development Council or Mandal Parishad or District Panchayat is the third tier of the Panchayati Raj system, and functions at the district levels in all states. A Zila Parishad is an elected body. Block Pramukh of Block Panchayat are also represented in Zila Parishad. The members of the State Legislature and the members of the Parliament of India are members of the Zila Parishad. The Zila parishad is the highest tier of the Panchayati Raj system and acts as the link between the state government and the village-level Gram Panchayat. See: http://north24parganas.gov.in/zila_parishad

30 Under the National Health Mission (NHM), each district within India has a District Medical Officer (DMO). The offices of DMOs are "responsible for planning and managing all health and family welfare programmes in the district, both in the rural as well as urban areas." The Chief Medical Health Officer (CMHO) is the DMO equivalent within Rajasthan, among other states. See: http://nhm.gov.in/index1.php?lang=1&level=3&sublinkid=1136&lid=144 and http://rajswasthya.nic.in/PDF/CMHO.pdf

31 The Right to Information (RTI) of 2005 is an act of the Parliament of India which sets out the rules and procedures regarding citizens' right to government information. See: https://rti.gov.in

what Abhay called the ‘vacant spaces’. The Village and Sanitation Committee have been there for long years. The Rajasthan Medical Relief Society, as a body, was constituted around various health facilities which then became the placeholder for the Rogi Kalyan Samitis as we went down the line. However, there has been one important difference, and this difference has affected the manner in which community action has rolled out. RMRS[32] (Rajasthan Medical Relief Society) was seen as a non-treasury mechanism for flexible expenditure for funds raised in whatever way, including local contributions—and I won’t go into the merits and demerits of local contributions for running the health system. That is a different debate. However, some money [is available] and to spend that, write up contracts, appoint some people on short-term basis, and get some work done with some planning and implementation capacity at the level of facility and powers around them. To a great extent, RMRS is an implementing body and therefore needs to confer and consult and discuss only in order to take decisions on how to spend those resources. Whereas the RKS (Rogi Kalyan Samiti) was visualized as a body that would review the overall performance of the health facility.

One part of communitization has been seeing how untied funds and other funds have been given over to these bodies. It includes VHSNCs,[33] Rogi Kalyan Samitis, and equivalently named entities. There is a certain amount of money handed over in effect from NHM to these bodies, and they are meant to spend it and achieve certain outcomes. This fundamentally alters the power relationship. From being a body that is meant to assess whether a health facility is doing well or badly, and to suggest improvements, this body instead becomes more of a local implementing agency. The accountability relationship reverses, and the tables turn when the health department says, “Look, have you spent the money correctly because the auditor is asking, and have you followed the rules?” So, we have seen [that] the VHSNCs and other similar bodies are involved in buying tables for antenatal care, chairs, and other things and reporting faithfully that money was correctly and properly spent. The money has to be correctly and properly spent. My point is that, once we go around handing over funds to these bodies, handing over a smaller part of a much larger fund—which is the fund to run the health agency and its facilities—, the accountability relationship changes and the people in these bodies are seen as contractual or implementing bodies or sub-contractors. Similar thing happened in the early formation stages when a pilot was rolled out. Abhijit has mentioned CHSJ being the technical agency supporting the pilot, and on behalf of my organisation, I was in participation with the AGCA while rolling it out in Rajasthan along with Narendra[34] and PRAYAS[35]. There were a selected number of districts. Even there we had a NRHM Mission Director of the time seeing the pilot as a kind of project wherein NGOs were trying to come in to help achieve goals of the National Rural Health Mission as it was known back then. He happened to see this and said, “You are going to help us achieve our goal. So, if you are there for carrying out community action activities, we should get higher immunisation coverage, higher antenatal care coverage, and more institutional deliveries. And I would like to see this happen.”

Rama Baru: One minute left.

Sharad Iyengar: If these aspects don’t change or improve and if the pilot is not succeeding, so is the matter of the state seeing communitization processes as helping to achieve public health

32 In November 1995, the Government of Rajasthan established the Rajasthan Medicare Relief Society (RMRS), which works to “provide various diagnostic and treatment facilities on nominal cost to general patients and free of cost to very poor (BPL) and dependent patients.” See: https://www.sarkaridoctor.com/rmrs-rajasthan-medicare-relief-society/

33 As per footnote d from earlier in this report, the Village Health Sanitation and Nutrition Committee (VHSNC) is a key institution introduced under NRHM to facilitate community participation in supporting, implementing, and monitoring health projects. It is formed at the level of the revenue village, and if the population of the revenue village is more than 4000, it can be formed at the level of a Ward Panchayat as it is in Kerala. From Government of India. (n.d.). Handbook for members of Village Health Nutrition and Sanitation Committee. Ministry of Health and Family Welfare.

34 Dr. Narendra Gupta, a community health physician, is a founding member of the NGO named Prayas (see note 34). Dr. Gupta is a national organizer of Jan Swasthya Abhiyan and the convener of its Rajasthan chapter. See: https://www.bmj.com/content/348/bmj.g2814.full

35 Prayas (Endeavour), established in 1979, is “a voluntary organization working for social, political and economic development in Chittorgarh district of Southern Rajasthan.” Prayas has advocated around a range of issues using a diverse set of approaches. See: http://prayaschittor.org/ourbrief.php

goals and objectives, which would again change the power picture. I feel that the positives have been in the very doing of this activity in the form of community action, coming first to pilot areas and then into larger areas. It has taken a grudging system, which has not been used to looking at sharing of plans and issues, time to accept and adapt. A system that has been compelled to recognise that decentralised planning at district and sub-district level is a reality and must be made a reality. Unfortunately, a lot of it remains on paper. At least there is an open window to look at it. The system has to take more steps to see that there is greater community engagement in the National Health Mission, which is a window to the health department. It has taken more openness, more transparency, more sharing, but going around saying that tables have turned, and power balance has changed is a far cry. I don't think that has come about. It is another discussion on whether it is likely to come about given the social, political, and economic circumstances. Especially with COVID not yet gone. Thank you.

Rama Baru: Thank you. Sorry that I have to keep interrupting about the time.

Rama Baru: I would request Mr. Nanda to share his reflections.

A.R. Nanda: Thank you Rama, I hope I am audible.

Rama Baru: Yes.

A.R. Nanda: I mean, it is a very interesting discussion that has been going on and we have got quite a lot, almost 90% or more of both about the genesis and evolution, functionality, and evaluation of the communitization process of NRHM. I don't want to repeat except just to fill in the gaps, because I was in the government when the initial, what you call, policy prescriptions were being built. Both in the wake of the movement of health which started in 1978, health policy in 1983, and then you have the Panchayat Raj and decentralization, ICPD Cairo[9] and post-ICPD reproductive health, reproductive rights movement, from target-free approach to community action, participation, and community needs assessment approach. At every stage, there was a quiet gap between what was intended and what was to be done. Community participation and community needs assessment became popular, and we could approach it in 114 districts. When I was the Secretary of Family Welfare, it was of course mostly restricted to reproductive health and family planning. However, when it was to be universalised into the health system with the NRHM, I think, it was getting designed and we had all sorts of problems. There were two sets of advisors and bureaucrats, and two sets of political leaders. Much effort was needed at that time to come together and understand each other at the national level, state level, and district level and sub-district level.

This thing has been going on with some success and some failures. On the whole, I remember the group—Jalaja, Amarjeet Sinha, Tarun Seem—they were all too supportive of this new paradigm coming up with communitization [and] community action. I could see Jalaja, Amarjeet Sinha, Tarun Seem attending the meeting of the Advisory Group on Community Action in Population Foundation of India. Almost every time one of them would be there. They will be actively participating. I am finding now being part of the same advisory [group on] community action, that this spirit has gradually been decaying, in the sense that sometimes at the best it's [a] Joint Secretary concern, most of the time it is not the Director or Deputy Secretary[36], sometimes very few junior officers come and attend. That too they come for five or 10 or 15 minutes and go away after making their speech. It is unilateral, it is just one side, not listening to what the Action Group wants to say. This has been a bane over the years, although the health policy the latest health policy speaks of this in some details. Secondly, the points that have been made about [incomplete sentence]. I remember when it was

36 Across career development stages of the Indian Administrative Service, a Joint Secretary is a senior rank relative to a Director or Deputy Secretary. The designations vary somewhat based on whether the officer is serving the district, state or central administration. At the central level, a Joint Secretary may have 16-24 years of experience as compared to a Director (13- 16 years) or Deputy Secretary (9-12 years). See: https://www.careerlauncher.com/upsc/life-of-an-IAS-officer/

being designed in the National Advisory Council[37], we had to take their help. We had to take the help of Jawaharlal Nehru University, where Rama Baru was part of the centre. We had to take the help of the Women Developmental Studies Centre[38]. We had to take the help of others—HealthWatch, People’s Health Movement—and we had to enter into the Prime Minister’s office. The Prime Minister heard our problems. There were powerful bureaucrats at that time who were opposed to this idea, who were not very sensitive to this idea, and they wanted to bring only family planning in a very targeted manner into the program of the NRHM and give a very minor role for the community action, for the communitization. Luckily, we got it into that, thanks to the other bureaucrats. Jalaja, Amarjeet Sinha, Tarun Seem... they were very proactive in this, and we also got the hearing of the Prime Minister. This is one of the matters of the genesis that I remember. However, to keep it up, to get necessary funding, initially they were thinking of having the Secretariat in the government as it is normally done and hold the meetings, but they agreed it would be in civil society, and Population Foundation of India was selected for that. Then there was the collaborative program of getting funding support for the Advisory Group on Community Action who are in the centre, states, and also in districts.

So that was a great welcome step that has sustained it over the last 15 years or more. As I see, as it is discussed, this requires very close collaboration; this spirit, sort of real efforts have to be there. Unfortunately, we find [that] although monitoring, community monitoring [and] accountability could go up to some extent and make some progress, both decentralization in the villages and all that [but] community planning, which is one of the very important things of community action, is yet to take deep roots. The government didn’t trust community action groups and they gave responsibility to NHSRC[1]. Okay fine. The Health Resource Centre also collaborated with the Population Foundation, [and] Secretariat of AGCA, and it made certain progress. Unfortunately, this is something in the same Nirman Bhavan... In the Health Ministry, there are two groups. One group, which wants to continue to target and approach in the name of expected level and achievement, and they don’t want to go beyond that because they say that is the accountability system that has to remain. The other group, which really wants decentralization to take roots in planning (planning of the health system, at least the annual plan). Annual action plan has not been able to succeed so far, and designing and planning.... So, this has been a severe, serious limitation and that is something which has not been taken up. Many of the states have really reconciled to it. Initially, the states were not very keen, except three or four states. So, thanks to the pilot program of community monitoring, things would spread and go above and beyond. I would say the Secretariat in the Population Foundation have been very active—they have been moving and going but that’s not the end of it. It has to be something which has to take deep roots in the Panchayat Raj system involving the Gram Sabha which are supposed to be the Village Health, Sanitation and Nutrition Committees. All these, it has to be sustained efforts, but what we see like every year, this year November has come, there are groups, and they are asking from action groups and community that you nominate a few people, and they will go to different states with our team, and they will make an assessment and they will also think of the next year’s action plan and see how it has been done. Which means that it is again a bureaucratic top-down approach rather than something which is a bottom-up approach, which is the spirit of communitization. I would like this to be taken in that sort of a spirit I am a great optimist, I am sure with the help of all the active, proactive members of the advisory group, others who are working in this line, in the states. And also,

37 The National Advisory Council (NAC) of India, which existed from June 2004 to May 2014, was a body established by the first United Progressive Alliance (UPA) government to advise the erstwhile Prime Minister of India, Manmohan Singh. It was a committee consisting of civil society members, ex-bureaucrats, lawyers, and academics. It acted “as a bridge between the civil society and the government.” See: http://www.allgov.com/india/departments/ministry-of-youth-affairs-and-sports/national-advisory-council-nac?agencyid=7592

38 The Centre for Women’s Development Studies was founded in 1980 by a group of scholars and activists committed to expanding and transforming accepted notions of gender-related research and action across the social sciences. See: https://www.cwds.ac.in

the government. Otherwise, it wouldn't be possible. Thank you very much, Rama.

Rama Baru: Thank you. Thank you so much, Mr. Nanda. We will now move to the next theme, which is really looking at the evolution of institutionalising of the community engagement process. May I request Rajani Ved to please make her remarks?

Rajani Ved: Thank you so much Rama, and it's such a delight to be here with everybody and listening to everybody walk down memory lane. I suddenly feel as if I'm schizophrenic, because I feel like I have so many identities, but where I am going to speak from today is really being part of the National Health System Resource Centre, where we were sort of bystander to the whole Community-based Monitoring and Planning Process, and I watched it over the last few years. [I] watched the ownership of the Community-based Monitoring and Planning Process diminish somewhat within the Ministry of Health, notwithstanding all of the efforts being made by the members of the AGCA. That has been a bit of a disappointment because I recall when it all began, and I'm sorry Rama, but I will have to go back into the A category because... a little bit...it's kind of an overlap, right, the beginning of it. I recall the very early discussions with the Village Health Sanitation Nutrition Committee and the ASHAs[39] and the communitization process of NRHM, who is not just CBMP[21]. It was ASHAs, Village Health Sanitation Nutrition Committee, and the CBMP all put together to enhance this communitization process. I think the original intent is...one can see from the framework document that the Village Health Sanitation Nutrition Committees' capacity would be built to serve as the bodies including membership of the Panchayat, chaired by the Panchayat. There was so much fierce resistance in the early 2005, '06, '07 from the Panchayati Raj department saying why do you want to create a VHSNC34 when you also have the standing committee of the Gram Panchayat to serve this goal. I think it was Mr. Sinha who argued strongly that every village must have a committee, as Gram Panchayats are too often far away from the actual village and therefore the VHSNCs came by. Much later, of course, the Mahila Aarogya Samitis[40] and the role of the ASHA... I feel the role of the ASHA was not capitalised enough in making her a staunch member of this whole communitization process, because now what's happened is that the ASHA is beginning to see herself as a part of the health system, and I think that is a huge opportunity lost because, notwithstanding all those titles of Accredited Social Health Activist, she was originally seen as somebody who would combine the roles of an activist, a mobilizer, and a link worker, and unfortunately now, it is more and more veering towards a link worker, notwithstanding all of the efforts to keep her rooted in the community. And I feel that that is an opportunity lost. That communitization process or the community-based planning and monitoring process could have engaged in strengthening ASHA a little more.

Another issue I think is... when this CBPM process started, the VHSNC was fledgling and in most of the country they continue to remain fledgling. I think that's another area where we had the opportunity to strengthen the VHSNC as members. After all, they are there and they also have membership from the community, from the local community, including Self Help Groups[41]. Going to the field now, I'm beginning to see again a little bit of revitalisation

39 The Accredited Social Health Activist (ASHA) programme is a key initiative under NRHM. As part of the ASHA programme, every village of India is to be provided with a trained woman activist (i.e., ASHA), who acts as "an interface between the community and the public health system." https://nhm.gov.in/index1.php?lang=1&level=1&sublinkid=150&lid=226

40 Mahila Arogya Samitis are women's groups of 8-12 locally-resident women, formed for every 100 households in vulnerable urban settlements for neighbourhood health planning, action on social determinants of health, and the monitoring of all local services. The municipal Accredited Social Health Activists (ASHA), or health outreach worker, is a member Secretary of the MAS, and a member is elected as Chair. The MAS is expected to increase uptake of government health services through creating awareness among the 100 families on all health and allied issues, and referring community members on a timely basis to appropriate service providers. A well-functioning MAS can support neighbourhoods to take ownership of their health and allied concerns through the empowerment of women, and thereby bridge the gap between people's needs and service delivery. See: https://nhm.gov.in/images/pdf/NUHM/Training-Module/Mahila_Arogya_Samiti.pdf

41 "A Self Help Group [SHG] is a village based financial intermediary committee usually composed of 10-20 local woman. The members make small regular saving contributions for a few months until there is enough capital in the group for lending." See: https://sewainternational.org/women-empowerment-through-shgs/

among the VHSNC where SHGs—because as [an observer] mentioned, they're becoming platforms in some states like Bihar, Tamil Nadu etc, even though it's all very fledgling still… They will then, with the correct capacity building, become members of the communitization process. I also recall early on discussions on civil society participation and civil society facilitation of the process, and no question, but all of the successes that we have seen in the last 15 years has been because civil society has led this process. There were discussions also in the Ministry about moving the AGCA back into either NHSRC or another arm of an autonomous institution of the government, but some of us resisted it really fiercely saying that there was this one platform that continues to be a civil society platform, autonomous of the Ministry of Health, and that's how it should be, funded by the Ministry, autonomous and retaining that identity. I just hope it stays that way. I am not very sure given the current context what happens.

Another reason why the CBPM process I think had challenges in scaling up was that the whole involvement of NGOs in NHM or NRHM actually faltered after about 2012-13. We have been part of developing guidelines for NGO involvement for over a period, and originally the NGO involvement guidelines were very focused towards strengthening community processes. Over time, they became much more towards strengthening service delivery, especially primary healthcare, and now they have disappeared from the scene. I think the last guideline for NGO engagement was written in 2013 and that is gone, and nobody is questioning why 5% of the funds of the NHM that were meant to be for NGO support are actually being used. NGOs have merged into the private sector as well. So private for-profit, [and] private not-for-profit has all been amalgamated into one whole. I think one learning from the experience is that the failure to speak to practitioners and street bureaucrats is actually something that we, I guess elites, have to overcome. I think [there is] this tendency to speak to senior level policymakers at national and state level, even at district level, but [not] the actual people who are engaged on the ground. If they don't invite this philosophy, then it really doesn't get done, and by this, I mean a Medical Officer of a Primary Health Centre, perhaps because they are the ones who the CBPM holds accountable often, or the district-level CMO or mid-level, even the ANMs[42], for instance. Understanding their issues and getting communities to understand the challenges that service providers and frontline workers face is another area. Okay, I'm going to stop. Thanks, Rama.

There is another area where we might [still have hope], and I don't think all opportunities are lost. I think there is a place and as Abhay said, it will happen. How long it takes to happen and who the players involved are will change, but COVID has shown us that it is really critical. One final point: I think in urban areas, we hardly even got into this process. It is clear to see that Mahila Aarogya Samitis are playing a very weak role in urban areas, as are ASHAs, and I think the lack of strong Civil Society Organisations in urban areas has something to do with this. I think this is an area going forward we need to take a look at. I don't think all doors are closed. I think the fact that the AGCA continues to survive and will probably do [so]… that is an area where we should all take hope [from] and say some things are possible. For the strengthening of Panchayats, the 15th Finance Commission[43] has given so much money. Of course, there's no money for NGOs in any of this, but the grants through 15th Finance Commission to rural and urban local bodies is really an area where we should be intervening. Considering States are actually considering pooling of funds at the state level and not letting money go back to the Panchayats is something that we should all be vigilant about and see how to work towards strengthening that. So, I'm going to stop there.

42 An Auxiliary Nurse Midwife (ANM), is a village-level female health worker in India who is known as the first contact person between the community and the health services. The ANM cadre was established to provide maternal and child health care within the community and at the facility level. See: https://chwcentral.org/indias-auxiliary-nurse-midwife-anganwadi-worker-accredited-social-health-activist-multipurpose-worker-and-lady-health-visitor-programs/

43 The Finance Commission, mandated by Article 280 of the Constitution, serves to "evaluate the state of finances of the Union and State Governments, recommend the sharing of taxes between them, lay down the principles determining the distribution of these taxes among States", and more. The Fifteenth Finance Commission was amalgamated in November 2017. The backdrop of its formation consisted of the Planning Commission's dissolution, the goods and services tax (GST) introduction, and more. See: https://fincomindia.nic.in

Devaki, not very sure I answered all your questions, but I thought this was an opportunity.

Rama Baru: We can come back maybe. I now invite Dr. Rajagopal. As you all know he was the founder of Pallium India and I think he brings in one state perspective and also a perspective of initiating a community-led process for palliative care and relating it to the legacy of NRHM. Thank you. Dr. Rajagopal?

M.R. Rajagopal: Dear friends, thank you for this opportunity to interact with you. I am a palliative care doctor. Palliative care is often misunderstood even among healthcare professionals. Most people think of it only as care for the dying. That's not the whole truth… it includes care for the dying; but we have to remember that the person is living till the moment of death, and that life is important to the last moment. We need to make that life as free of pain and suffering as possible through the continuum of the disease; that is palliative care.

Hence, we should be talking about making palliative care a part of healthcare. For this, certainly, there should be a top-down approach advocating for favourable legislation and policies. But there should also be a bottoms-up approach. My colleagues and I demonstrate this approach in Trivandrum in Kerala by direct patient care and by educating stakeholders. Elsewhere in the country, we collaborate with interested individuals and institutions to facilitate development of palliative care. Till I started doing palliative care at the age of around 40, I did not really know how our people live or how they die. That was new knowledge for me when I walked down alleys in towns and villages and met them where they were. Most experts, governors and managers would not really know the problem on the ground.

There was one major difference between the Alma-Ata declaration of 1978 and the Astana declaration[44] of 2018. The latter recommended not only engaging and partnering with the community but also advised giving control over healthcare to the community. When we do that, when we work with local groups from every village, we actually see the miracles that they can perform. They can see that the healthcare system does not provide true healthcare; but instead provides only disease-focused care. It ignores suffering almost completely. However, the community would not ignore suffering. The community can also see that the government cannot easily provide true healthcare because, as the Astana declaration says, it requires collaboration between various departments—transport, food, sanitation, irrigation. Can governments easily achieve that when different departments work in silos in different buildings? But the community does not have that problem. They are able to see that somebody is starving; they can see that another person does not have safe water to drink. When we facilitate their activism, they achieve true healthcare, provided we are there with them to give attention to the disease and to physical suffering like pain, breathlessness, nasty foul-smelling ulcers, etc.

The point I was trying to make is, healthcare has to include prevention and relief of suffering. Without that, healthcare would be a heartless monster. There are three other points that I want to make. For one thing, healthcare must reach the vulnerable population. Our health care system today does not reach 96% of people in serious health related suffering. They do not have access even to basic pain relief. That being so, what about people with disabilities, those with different sexual orientation, those who are socially and politically isolated in prisons, or those having stigmatising diseases? They are almost completely rejected by the existing healthcare system. But community activists will not. They can see the need. They will give special attention to the needs. They are able to see the needs because they go to the needy 'where they are, when they need them'. They can hear the needy because they choose to listen.

My third point: let us not romanticise the community. If we put the community on a pedestal and think, 'This is so great; let us give the community power and stand back and watch',

44 In October 2018, the Global Conference on Primary Health Care provided a space for government decision makers to collaboratively adopt a new declaration in Astana, Kazakhstan. The new (Astana) declaration expands upon the 1978 Alma-Ata Declaration (see note 7), reemphasizing the crucial role of "primary health care [in] addressing current health challenges, renewing political commitment to primary health care, and achieving universal health coverage." See: https://link.springer.com/article/10.1007/s00038-020-01368-5

we may have to see the initiative disintegrating over time. We will have to anticipate the potential challenges. There is a question of balance of power. Those who are in power may tend to abuse that power. This could be true for community activists or groups also. If they become the new lords and avoid involving people with disabilities or those with different sexual orientation, the initiative will tend to rot. What is the solution? When we plan the strategy for public or community participation, we have to lay ground rules in black and white. We as facilitators, will have to monitor the program and provide guidance. The community-based organisation has to accept responsibility for maintaining basic core values, ground rules and mutual respect, even when there are disagreements amongst themselves or with the government.

My fourth and final point: The Prime Minister’s new Atmanirbhar Swasth Bharat Yojana[45] gives us hope. It actually reiterates something that the World Health Assembly Resolution-67[46] of 2014 said. Whatever we do needs to be available across the continuum of care, starting with prevention, and mitigating any illness related suffering from its beginning to the end as part of routine healthcare. And it has to be integrated into healthcare at all levels—primary, secondary and tertiary.

Rama Baru: Thank you so much. May I request Mr. Tarun Seem now to please make his remarks?

Tarun Seem: Good afternoon, everybody. It is, in a sense, a déjà-vu for me to listen to my senior colleagues after so many years, reminiscing about the thoughts that went into designing and drafting of the Mission and its implementation framework, and then the early part of its operationalisation. I was associated with NRHM up to 2010, starting from January of 2005.

So, I missed the Samudra hotel meeting, although I did participate in the eight task groups and their formation. So, that’s when I was born. When I left, NRHM was a flourishing organisation; it was doing very well. The Program Implementation Plans (PIPs) [47] were being organised in a very robust, structured manner. The discussions were very detailed and very enlightening, both for the States who are coming to present their plans and for the Government of India, which was to give the funds. I believe I could see the process of decentralization actually being born in those PIP discussions and become stronger and stronger. Things have changed substantially since then.

Be that as it may, I would stick to the two topics of evolution and its impact experiences. [With] community action [and] scaling up, has the goal been achieved? What are the challenges?

This activity is not a project. Community action is not a project. Unlike traditional government projects, community action is a process. I think community action in the health sector, from what I saw in the NRHM, is a continuous process. It would have continued for “always”. Why you see it going down now is because you think that the task is done, and one can withdraw budgetary support or handholding support. But no, community action is one of the most delicate of the interventions and needs to be handheld for long periods, exceeding perhaps a decade. Decadal progress in implementing a framework for community engagement would be a short period by policy standards. You have to nurture stakeholders who will come and go in the period. New stakeholders, therefore, require to be nurtured on a day-to-day basis for community action to get embedded in the processes of governance.

We have seen community action being included in the PIPs. Original PIPs did not have this logic but with AGCA becoming stronger and becoming more articulate, it started having a role to play

45 The Pradhan Mantri Atmanirbhar Swasth Bharat Yojana (PMASBY), announced in February 2021, is a government scheme launched to enhance access to healthcare facilities within India. It was approved by the Union Cabinet in September 2021 with a total outlay of Rs 64,180 crore for six fiscal years (up until Fiscal Year 25-26). PMASBY’s primary aim is to close existing gaps in public health infrastructure within urban and rural regions. See: https://www.pib.gov.in/PressReleasePage.aspx?PRID=1704822

46 See 67th World Health Assembly summary: https://apps.who.int/gb/ebwha/pdf_files/WHA67-REC1/A67_2014_REC1-en.pdf and https://apps.who.int/gb/ebwha/pdf_files/WHA67-REC1/A67_2014_REC1-en.pdf

47 “Programme Implementation Plan (PIP) process facilitates the planning, approval and allocation of budgets of various programmes under the National Health Mission (NHM).” PIPs support the standardized organization and implementation of NHM program. See: http://pip.nhm.gov.in/#:~:text=Annual%20Program%20Implementation%20Plan%20(PIP,states%20against%20the%20approved%20PIPs

in appraisal of the PIPs, where it could check whether community processes have been adequately budgeted in the state PIPs or not. That was a very welcome change, and as Sharad pointed out, the state's authorities, and the state ecosystem of community strengthening, was the determinant. AGCA was perhaps just really doing only monitoring. In that sense, the states' overall capacities were being reflected in their progress in community action.

What were the challenges? People tend to lose interest in something as delicate as community-based action. If we have a project at hand, you have budgetary support, you have cabinet approval, you go out and do it. You install it, you construct it, or you operationalise it. On the other hand, in community action, you have to take the community along. That means you have to do an enormous amount of stakeholder consultation, stakeholder handholding, just probably stand with the community. This really is the eventual goal. If this original goal of the whole enterprise is lost sight of, then we tend to lose interest in that exercise and assume mid-term achievements as end-term goals.

Also, when you fail, you tend to lose interest. I remember in our Bhubaneswar meeting[48], some people were very critical of the scale of monitoring activities that was done, even though it was not monitoring in any real sense. We had other methods of monitoring. We had our MIS [Management Information System], we had our CRMs,[49] we had other surveys available to us. So, CBM was only offering a triangulation platform, which was good enough for whatever it is worth. However, that worth was worth its weight in gold as far as overall health and sustainability of the program is concerned. As Rajagopal Sir pointed out, if that community connect is missing, then the rest of it being robust also doesn't really matter. So that failure, which was articulated in Bhubaneshwar conference, was often quite disheartening. Later, other activities come in and become more important. There was desire to expand the program, you want to bring in lifestyle diseases and other issues. The changing of a "system reform" agenda into a vertical program is a very short distance, really. NRHM was under serious threat of going that way. Community-based action is highly dependent on champions, so without them it doesn't work out, and it is also very prone to high level ideological changes. So, if the high-level policymaker is not interested, then it really goes away.

The second part on evaluation and impact. What are the key successes when it is demonstrated the format of community engagement? How it could be done in a structured manner using government money and embedded in PFI very competently, mentored by the PFI Secretariat and the other members of the organisation?

Has power sharing been internalised? Not yet, but that task is being increasingly shared with the civil society and community institutions, and I think that is the proof of pudding. It has been a big success in that regard.

There are three things I see as reflections of a serious disease that can only be handled when the community-based action is further strengthened. First, is the poor allocations of Urban Health

48 In 2009, a conference was held in Bhubaneswar on Quality, Equity, and Accountability under the NRHM. Many states and NHSRC delivered presentations. Dr. Tarun Seem delivered a presentation on community action under NRHM on behalf of AGCA. Many members of AGCA also delivered presentations, especially about the activities of community monitoring processes during the pilot phase. The workshop had sessions attending to issues of operationalising protocols for rational drug use, childcare and JSY, quality standards and community processes, and discussion on findings from the 2nd Common Review Mission (CRM), operationalising web-based HMIS, and financial reporting under NRHM, among others. (Source: Information provided by Dr. Tarun Seem)

49 Common Review Missions, conducted annually since 20107, are a unique monitoring and learning modality embedded in the NRHM planning cycle. Teams comprising government functionaries, public health experts, civil society members and development partners would visit districts in the union to physically observe and assess the achievements and challenges faced in the implementation of the National Rural Health Mission. They also represented a crucial platform for dialogue and experiential learning for actors in the system (both the reviewers and those reviewed). CRMs are coordinated by the National Health Systems Resource Centre. See: Singh et al.: Common Review Mission: reflections on a concurrent evaluation measure. BMC Proceedings 2012 6(Suppl 5):O20. DOI:10.1186/1753-6561-6-S5-O20

Mission[50] out of the total budgetary allocation. Second, is the manner in which ASHAs who were accredited social health activists are turning into line functionaries and making staff unions, and third is the overwhelming role of consultants in preparation of the PIPs. Reforming such problems will need time and money, and support at the highest level. That would need a budget line for consistent community activities, both at Centre and in states. The state AGCAs, whether the state is ready for them or not, need to be pushed and therefore there is a lot of hope in this enterprise, but there is a long journey to go. That is what I thought about evaluation and impact. Thank you.

Rama Baru: Thank you, Tarun. We move on to the third team. Actually, they're all linked, but we are looking at so many years down from NRHM, so many differences in terms of state involvement. So, if we were to evaluate where we are today with respect to where we began, I'd like to request Poonam Muttreja to please come in and share her reflections.

Poonam Muttreja: Thank you, Rama, and thank you Misimi and other colleagues for inviting me. I think it's an absolutely fantastic group that's been put together, and I would like to say that I echo and agree with everything that has been said, except perhaps, some of it seems disagreements like a practice. Even in Advisory Group on Community Action (AGCA), Abhay and I have very good arguments, and I would like to say today, to again start with Abhay—who is, by the way, one of the most active and amazing AGCA members and whose work in Maharashtra on community monitoring is exemplary within AGCA. However, I would like to say that we don't have to totally step out, Abhay, as you said, of the system and work outside. We need to continue doing both, and I think we have the strongest foot anyone has in the system—it is the AGCA and the community-based monitoring and planning (CBPM). Why do I say that, Rama? I say that because this is one of the few spaces that not only exist for NGOs, but I think in spite of efforts—and whoever's effort it was to move the Secretariat into government or National Health Systems Resource Centre (NHSRC)—I think it is the strength of AGCA and its work and the Secretariat that has led to the reversal of the decisions that had been done in a file to move it into a more government system. We still have [an observer] and Tarun Seem's and Mr. Nanda's in the system. Preeti Sudan (Secretary Health and Family Welfare), led by her and Mr. Manoj Jhalani (Additional Secretary and Mission Director, National Health Mission) and other colleagues, other bureaucrats who believe in community-based monitoring and accountability by the community, had done something which is, as you all know, very difficult to do in bureaucracy.

Second, I would like to say that both as a lesson, but also as an assessment, but lessons for the rest of the world is a question that Devaki and her colleagues have given me. I would like to say that having reviewed, looked at, and participated in community-based processes across the world, this is the biggest accountability initiative that has gone to scale as Abhijit, who was very active and worked with Mr. A.R. Nanda, my predecessor at Population Foundation of India on the pilot, will know. We piloted CBMP in nine states, 36 villages and 36 districts, and 1600 villages. We are now in 24 states, 372 districts, and 72 cities, which is recent work, and we are in 2,25,000 villages. Now, scale without depth is not the only measurement of either success or an example for the rest of the world, but it is. I believe that there are many states in which CBPM has got embedded as a process. The whole process leading up to Jan Samvads[51]... the process—as [an observer] said—is as important as the plan and the nature. And the fact that we were able to do Jan Samvads across the country, even during COVID on online platforms, shows that the

50 The National Urban Health Mission (NUHM), alongside the National Rural Health Mission, comprises the National Health Mission (NHM) of 2013. NUHM is intended to fulfil the health needs of urban residents, especially those experiencing poverty. Specifically, NUHM works to increase the availability, access and quality of primary health care services, while reducing out-of-pocket treatment expenses and addressing health determinants more holistically. See: https://nhm.gov.in/index1.php?lang=1&level=1&sublinkid=970&lid=137

51 Jan Samwads are public dialogues through which community members can share feedback on health services under the National Health Mission (NHM), which encompasses both NRHM and the National Urban Health Mission (NUHM). For more information, see: https://nrhmcommunityaction.org/bridging-the-digital-divide-connecting-communities-with-health-systems-through-virtual-jan-samwad/

system was more ready, and I do not want to only talk about the community-based action in these states.

I want to add two other aspects which I think the AGCA and civil society need to take more... perhaps more seriously, to be a little self-critical. We have an opportunity to be part of the CRM (Common Review Mission) as AGCA. This is to be recorded as an important opportunity which only a few of the AGCA members are able to participate in; however, the Secretariat does go, and it gives us a huge opportunity to give input in the evaluation of National Health Mission (NHM), specially of CBPM, amongst others, and integrating CBPM. Third, I would like to say a very important area on work and functions of the AGCA Secretariat. Bijit Roy will give the details of what the community monitoring process is. He is the one who leads the work, and it is the Secretariat that does the work. This is an important case study for the world because [of] the way the AGCA members [participate]. Each of them is really busy, but I can tell you they participate across the states, not just within the state. Sharad mentioned he and Narendra were part of the Rajasthan process, but AGCA members work across states. AGCA members are the ones who guide the Secretariat, and I would like to say that this is truly a Secretariat which does not belong to one organization, located in Population Foundation of India, but it is monitored, supported by all the AGCA members. I would also like to say, Mr. A.R. Nanda, my predecessor, is responsible for the Secretariat being in PFI. Had someone else been there [in] PFI, I don't know if they would have given us the Secretariat. So, we have to thank Mr. Nanda's credibility and background with the government and his vision in having the Secretariat at Population Foundation of India. There. The fourth area that I would like to point out is the opportunity we have and which the AGCA has very creatively used, to do in the fact-finding missions when there have been disasters. I would like to mention Barwani, where AGCA did an inquiry when 29 women died during childbirth in a district hospital. The second time we did an inquiry was in 2014, when 16 women lost their lives during a sterilisation camp in Chhattisgarh and the two inquiries led to major changes in Madhya Pradesh and at the national level. Our report was submitted to the Supreme Court of India bench headed by Justice Madan B. Lokur Bench, and I think for those of us who work on family planning and advocate for a change, doing away with target-free approach, doing away with camps in one stroke, what that report and the work of several other NGOs contributed to [was] the landmark judgment where the government has also been held accountable.

So, I think when we do the history or analysis of the CBPM, we need to factor that in. I would like to end by saying the glass is less than half full. In spite of the scale-up and achievements, I would like to say this is a very good time to reflect. I was speaking to [an observer] yesterday when I asked him to guide us and let us strive towards excellence and take the opportunities that have been presented to us in this rather have an adverse situation for NGOs. We have a great opportunity, and that is how I see it. Thank you, Rama.

Rama Baru: Thank you so much, Poonam. May I invite Mr. Bijit Roy to please make his remarks? Thank you.

Bijit Roy: Thank you so much, everyone. It is an absolute delight to be with you again. I would like to first take on what Poonam mentioned: sharing some facts on where we stand in terms of the budgetary allocations for the implementation of Community Action for Health (CAH)/ community-based monitoring and planning under the National Health Mission (NHM). It is not as if all is lost. If we look at the previous Financial Year (2020-21) NHM Programme Implementation Plan (PIP) and current FY (2021-22) PIP, there's been a 26% increase in allocations to the states, from [Rs.] 270 crores to [Rs.] 342 crores. These costs are for activities and facilitation costs. There is a growing interest in the states in community action, and with COVID, the Advisory Group on Community Action (AGCA) team has reached out to most of the states in the country with resources materials [and] technical support. Poonam did mention even engaging with state governments' accountability aspects related to social audits and public feedback to providers.

The second point is around the results and

evaluation of CAH processes. There have been three systematic evaluations over the last 15 years, which I can recollect. The first is the pilot phase which Dr. Rajani Ved was part of, which looked at the pilot phase of CAH (covering 18-month implementation), which was supported by the Ministry of Health and Family Welfare. The second was done in Maharashtra, of SATHI’s work and the coalition’s work. The third was an evaluation undertaken by Mathematica[52] for Population Foundation of India’s work in selected districts of Bihar. Now, there are four or five key lessons or successes that we would like to draw up, and I would like to highlight each of them.

First, the process of community action and monitoring brought about an environment of trust and interaction between service providers and health providers, between the community and health providers, to look at service delivery, to understand and increase the coverage and range of services. The second important thing we found was, when the communities started engaging with health systems and started monitoring and giving feedback to health officials, there was an overall improvement in the range, availability, and quality of services. The hospitals started functioning. The doctors came for at least a couple of hours. People in places in Bihar like in Darbhanga district, doctors from Community Health Centres (CHCs) were brought to PHCs to at least open the PHCs for two days a week and provide outpatient services. Small changes started to happen. The local planning and action started happening with the funds that were allocated to the Village Health Sanitation and Nutrition Committees (VHSNCs)34 and the Rogi Kalyan Samitis (RKSs). We have seen very positive experiences across the country, especially even in states like Maharashtra as well as in Uttar Pradesh where you see the funds for the Rogi Kalyan Samiti is being used for patient-centric work such as improving infrastructure, procurement equipment’s, medicines, and emergency transportation. The fourth thing that we would mention is that the social audits have brought the people’s issues to the decision makers/bureaucrats, and a lot of action has happened around posting of doctors, renovation of sub-health centres, PHCs, reimbursement of pending incentives to the ASHAs, and community making sure that guaranteed services are available at public health facilities. The other part I would like to mention about is regarding the Civil Society Organisation (CSO) and the government engagement as it has evolved over the last 10-15 years. So, from the pilot phase, which was primarily led by CSOs, state governments have taken initiative to scale up implementation of community action processes through state level institutions. I would like to take the examples of State Health Resource Centres in Chhattisgarh and Odisha as well as Kerala Institute of Local Administration[53] (KILA) where systematic community monitoring and action are being done at scale. [An observer] also mentioned the social audit carried out by the Department of Rural Development. The AGCA has facilitated partnerships between the State Social Audit Units and National Health Mission Meghalaya[54], Uttarakhand[55] and in Jharkhand[56] to conduct social audit of health services, which has been very promising.

Lastly, I would like to close with an important point engagement of CSOs. A lot has to be done, not only in terms of the reach and capacities of CSOs, and also to engage with systems, but how do we create an intent and conducive environment within the government to engage with CSO I think a lot has to happen around creating that trust and conducive environment for community monitoring action to flourish in the next phase of the National Health Mission and under the Pradhan Mantri Jan Arogya Yojana. Thank you.

52 Mathematica, formerly known as Mathematica Policy Research, is a United States company with headquarters in Princeton, New Jersey. Mathematica provides research organization and consultation services in the areas of data science, social science, and technology, shaping social policy initiatives. See: https://www.mathematica.org/about-mathematica

53 Kerala Institute of Local Administration (KILA) is an autonomous institution functioning for the Local Governments in Kerala. It was registered under the Travancore-Cochin Literary, Scientific and Charitable Societies Act 1955. See: https://www.kila.ac.in/about-us/

54 Meghalaya is a state in northeastern India.

55 Uttarakhand is a state in northern India crossed by the Himalayas.

56 Jharkhand is a state in eastern India.

Rama Baru: Thank you very much. And I think this has been a very engaging conversation. I think we have a little bit of time for people to come in and react to what others have said or add to something that they wish to say. Sorry about this seven-minute restraint. It wasn't a happy job to do. But I now open the floor for those who would like to come in, add or react to the other speakers. Sharad, would you like to say something?

Sharad Iyengar: It is kind of a finite point. I don't mean to by saying this claim to be reacting or responding to the very large issues that people have brought out. The COVID pandemic saw a kind of change in the manner in which the narrative on the medical profession and healthcare [and] professional health care workers has changed, in the eyes of the larger community. I know that with various forms of media. This is a kind of manipulated narrative, but there has been at various points, a very negative portrayal of the health system, especially the private sector, and this has been rapacious, backed by evidence in various ways within the healthcare profession, especially the medical profession. I have access to such discussions and assume other doctors have this feeling of persecution that people go on holding doctors accountable, whereas they are small players in the larger system, which is driven by corporates or by insensitive governments. Therefore, health workers are victimised, and during COVID, you had healthcare workers being seen as heroes. However, many years before, when there was talk of why and how it makes sense to engage with the community and give the community a close look at how health plans are being made, what the indicators are, and to invite participation therein. There was one effective argument, about the fact that often when there's breakdown of a minimally cooperative relationship between the patient community and the health provider community, you have in times of crisis, violence breaking out, and that is very damaging. This happens typically in health facilities; it typically happens around emergencies. It is often backed by political forces. A group of people go berserk but the brunt is paid by front line providers of a health system. They are not necessarily the ones who manage the resources or take the decisions to keep the health system going. Community action and processes related to community-based monitoring and planning was seen as a way of reaching out to the community and coming out with a more peaceful way of airing grievances and listening to them. In a way conveying to the community, "Look, it is not the frontline providers who are calling the shots. They may be in the front, but there are a lot [of people] behind them who are responsible, and it is not a simple and easy job to make sure that all the equipment, all the drugs and all the providers, and all the decisions are correct and perfect, especially in times of crisis." This needs to be worked through, and the community could better appreciate difficulties of delivering healthcare or the issues in delivering healthcare. So, this is a way of improving dialogue which would lead to grievances being added of course, and being addressed, but without coming to violent flash points. I think even though this may not be so very glorious in intent or concept, it is a very practical way—one practical way, not the only practical way—of furthering the cause of dialogue between providers and recipients, users of care, across various fora. I don't see much of that having happened, but still think it has been an important driving force.

Rama Baru: Thank you. Yes, Poonam.

Poonam Muttreja: So, you know, one of the points that Dr. Rajani Ved raised, which is the 15th Finance Commission, and the fact that huge devolution, finally, of fiscal responsibility will take place for the Panchayats. We know that there is funding in health, not for NGOs, but definitely for Panchayats. So, the whole CBPM process, we need to redesign, keeping that in mind partly. Second, I am wondering if there is a dilemma within AGCA around involving Panchayats actively, and I want to bring it up here as a point to think about. As the Secretariat Convener, I am happy to report that, while the resource commitment by the MoHFW for community action for health under the state Project Implementation Plans (PIPs) have increased from close to [Rs.] 70 million in 2009 to [Rs.] 682 million

in 2021, the budgets for the AGCA Secretariat have continued to decrease. If PFI didn’t have its own resources, which we subsidised 50% at this point of the expenditure, it would be very hard. So, do we take external funding, but then it dilutes government ownership accountability in a way. There is the funding question and dilemma that AGCA experiences. Finally, I want to mention that I think it is very important—just like Dr. Rajani Ved and a group of experts did an evaluation after the pilot was implemented many years ago, I think it was 2009 that the evaluation took place, and it’s 2022 now, and it is time to do a really robust and good evaluation. As we think of, the post-COVID scenario, we all know that we have to reimagine our public health system. Similarly, we need to reimagine the CBPM program. The re-envisioned process would be better if it is followed by an evaluation. Now, how will that be done? Who will do it? Where will the funding come from? Should we engage the government in this process are some of the questions I want to throw up. Thank you, Rama.

Rama Baru: Thank you, Poonam. I think this is an important issue, but I like to add just my last remark that this funding issue is also very critical for the survival of CSOs. Because I think with the changes in FCRA[57], there is quite a bit of flux within the Civil Society Organisations. There are great concerns about their future, their survival, including, you know, what sources of funding they can access and how it can be utilised. At this moment, we are in a certain kind of a flux, which I am sure all of you who have been engaged and involved with the NGOs and civil society groups are debating this fairly actively. So, if there are no further interventions from others, I want to thank all of you. I also want to hand over the proceedings to Misimi from The George Institute. Thank you very, very much. And it was a very, very engaging and thought-provoking Witness Seminar.

Misimi Kakoti: Thank you, everyone. Thank you all for taking time and participating in this session and thank you all for sharing your experience and reminiscing particularly. We will follow up with you. We are going to transcribe this recording and then we will follow up with you for the review, and as we annotate this documentation with references and finalise the report, we will be in touch. Thank you so much. Thank you all. Bye.

Proceeding ends.

57 The Foreign Contribution (regulation) Act (FCRA), 2010 is an act of the Parliament of India, by the 42nd Act of 2010. It is a consolidating act whose scope is to regulate the acceptance and utilisation of foreign contribution or foreign hospitality by certain individuals or associations in India. See https://www.thehindu.com/news/national/the-hindu-explains-what-is-foreign-contribution-regulation-act-and-how-does-it-control-donations/article32590504.ece.

Annexure

In-depth Interview with Dr. Abhay Shukla

Transcript starts.

Misimi Kakoti: Following from the Witness Seminars, you had mentioned about the 'multilevel sandwich strategy' in the context of having the CBMP process rolled out in Maharashtra, where you mentioned about combining advocacy from above state level, and then in order to open up participatory spaces and in your words, "very active community mobilisation from below." Could you zoom in and tell us, in specific, what was your experience, with respect to this point that you made, in the context of Maharashtra or broadly at the national level also?

Abhay Shukla: So, I think that was one of the somewhat positive features of the CBMP process, as it was designed across the country, and as it was, I think, implemented with some reasonable degree of effectiveness in at least a certain phase of time in Maharashtra. So, what I meant by the 'multilevel sandwich strategy' is that, for example, we started with the national-level design. What we have to understand is that for anything like community-based monitoring to unfold in an official framework, two processes have to be simultaneously facilitated. So, one is that a closed system of power, which is the health system, has to at least open a small chink in the door and allow for a certain level of sharing of power with non-official and community-based actors. So, that is an opening if you're opening up a system of power. This is the crux. Without some sort of equalisation of power, and without some sort of opening up of a closed system of power, there can be no institutionalised accountability. However small it is. I'm not overclaiming it. But what else? So, opening up those spaces which requires—in any vertically organised system—action from the top, you see. States operate from the top to bottom, and communities operate from the bottom to the top. It is completely opposite. Therefore, the facilitators have to be adept at doing both. You get it? Because the second process is to enable community-based actors to occupy those spaces, to feed their voices into those spaces and to intervene in those spaces which are opened up. So, if we have only done community mobilisation and no opening up of those official spaces, then CBM would not have happened. Something else would have happened. If you had only done opening up those spaces, but no community mobilisation, then you would have had some nominal spaces and committees, but nothing substantial would have changed.

So, the very complex role, which was played by state nodal NGOs, district nodal NGOs especially, and also—to some extent—AGCA members wherever the process was actively promoted, was simultaneously like dancing two different dances at the same time. Half the time you are doing bhangra[58] and the other half you're doing salsa. You are, on one hand, talking with the Union Health Ministry, with the State Health Ministry, with the state NHM, state-level Mission Director, Director of Health Services—these kinds of officials—and convincing them that you have to open up these spaces, you have to form these community-based monitoring committees, you have to activate the VHSNCs, you have to open the RKS, and so on. So, that is one process going on and, at the same time, we were reaching out to communities, working directly with people at the village level, with RKS members, with Panchayati Raj members, and telling them aapke health rights hain, issko le kar aap mobilise karo [you have health rights, you need to mobilise to claim them]. You have to claim your health rights. This is a space to claim your health rights. So, opening the door on one hand and inviting people to walk into that door on the other hand—and people means actual people at the ground—this is the kind of process that we did. Now, this is actually... what is called 'sandwich strategy'[59]...is not exactly the same thing, but it is something similar, although it

58 Bhaṅgṛā is a Punjabi traditional folk dance, originating in Punjab's Sialkot area and associated with the harvest season.

59 According to the Accountability Research Center, "the sandwich strategy relies on mutually-reinforcing interaction between pro-reform actors in both state and society, not just initiatives from one or the other arena." See: https://accountabilityresearch.org/sandwich-strategy-research/

has been used in a slightly different context. And why I’m calling it multilevel is because at each level [there is an] opening up of the spaces—it’s not only two levels, that is, national level or community level. In between that also there are several levels. At the very least, there are three levels, which are the state, district, or block levels. At each level, we have to do the same thing. You get it? Just because you have a state level sanction, doesn’t mean that the District Health Officer is on board. The District Health Officer also has to be convinced that… See, you have to make a district-level community-based monitoring planning committee, you have to attend district-level Jan Sunwais[24], you have to check district-level report cards also and you have to take action on that. But we use the sanction from the state level.

At the state level, we use the sanction from the national level. At the district-level, we use the sanction from the state level. At the block level, we use the sanction from the district level. You get it? So, at each level, this has to be done. I mean, these histories should be written sometime, and each level is not the same. It’s not automatic. India is a federal constitution and state governments do not automatically start doing something because the central government has said it needs to be done. It has to be translated, and it has to be also recreated at each level. The CEO at district level will be the IAS officer and he is the king of that district, and he won’t automatically start doing what you want him to do. We have to employ all kinds of strategies to get all these levels of actors on board so that those spaces are created or opened up. And then we had to do the mobilisation bottom up. It’s not sufficient that we only do mobilisation in villages, the issues that come out from the village level mobilisation need to be taken to the PHC, and from five different PHCs you need to combine the issues and take it to block level. Then [the issues] from three blocks combined needs to be taken to the district level. You need to find out the common issues and identify what’s serious. You need to find the systemic issues and their solutions and then present it at the district level. Then collect issues from five different districts and present it at the state level. This is the bottom-up process, which we also follow. This is what I very critically called the ‘multilevel sandwich strategy’. So, there is action from above to open spaces, and there’s action from below to occupy the spaces.

Misimi Kakoti: Right. Okay. What kind of advocacy design is needed for that? Devaki, do you want to come in?

Devaki Nambiar: Yes, sorry Abhaybhai. I’ve been listening though, very excitedly. I was wondering: in terms of creating that team that facilitates this and brings them on board, are people crossing across levels? Could you talk about the history of who was on board? How did they kind of join this piece, in Maharashtra specifically?

Abhay Shukla: This is something which we did not have time to talk about that day. There was a precursor to community monitoring.

Devaki Nambiar: Exactly.

Abhay Shukla: It did not arise in a vacuum. It arose in a complex social, political, and institutional kind of context which was shaped by Jan Arogya Abhiyan[26] and Jan Swasthya Abhiyan[11]. There was a coalition which was active in Maharashtra from 2000 onwards, which had organised the National Public Consultation with NHRC in 2003 in Mumbai, also organised the Western Region Public Hearing in Bhopal, and the state health department was also there. Of course, all the CSOs and Jan Sangathans [mass organizations] were also there. Then, after the 2004 dialogue in the public hearing, we started having regular CBM type of dialogue in a few districts of Maharashtra with health officials. It’s something which many people do not know. So that, you can call a proto-CBM or whatever you want to call it, like a precursor of CBM. So, we had the dialogue in Bhopal on 29th July 2004, we had the western region public hearing. All of Maharashtra, Gujarat, Madhya Pradesh and Goa’s officials were there, and Jan Swasthya Abhiyan’s activists and representatives were there, and there was a dialogue with the Maharashtra DGHS [Directorate General of Health Services] where he said, “You have brought up these issues in Bhopal whereas you should’ve spoken to the state officials,” and when we spoke to the state, they said

you should talk regularly to the district officials. So, in Thane district and Pune district, our JAA constituents collected all the issues, big and small, and they started having a dialogue with the district health officials on a periodic basis. This was a kind of a CBM because from community levels, issues were identified and presented on a district level, [and] then some action will be taken on it. Then it'll be taken back to the community level to tell the people that, "Look, this is the order that's come and based on this we'll get this service." It used to go back and forth. So, that was the background. So, there was a health movement coalition which was ready in Maharashtra and also in some other states which was ready to take up this task, and which was ready to do this two-way translation—community's issues translation into officials' language, and officials' orders and NRHM's provisions into peoples' language. That is the advocacy design. Sort of what you are asking about. And if you want some relatively agile organisations which could operate across multiple levels—so SATHI[3] was one of those organisations in my understanding, which had that capacity to operate at multiple levels from the national and state level to the district and subdistrict level.

Devaki Nambiar: In the beginning, were there some issues that were paramount?

Abhay Shukla: Issues as in, specific health system related issues, like demands?

Devaki Nambiar: Yes—oh, so it starts off as a grievance only?

Abhay Shukla: One very interesting thing: people's imagination about health services is something which is shaped by what is available. It's not something static. So, it's not that people in the village know that "Oh, I'm supposed to get these 17 services in my PHC." People usually are not aware of that. It's just a very broad idea that once you're there, there will be some treatment.

Devaki Nambiar: Yes, like, "I didn't get this," or "I didn't get that service."

Abhay Shukla: Yes, you will want that in a PHC there is 24 hours normal delivery. They will say, "That doesn't happen here at all." You will say, "Children should get vaccinated here", and they will say, "Yes that happens occasionally." Then you will say, "There should be an ambulance available to take you to the next referral", and they will say, "But the ambulance here doesn't run at all." So, people's imagination and understanding of what they can expect from the health system is also dependent on how the system actually reaches out to them and communicates with them. And that's the important part of what we did under CBM. We expanded people's imagination. The fact that healthcare is their right is a new concept for them. That's not how ordinary people look at that sarkari dawakhana ["Government dispensary" in Hindi]. It's like a doctor usually sits there and if we go there, we might get some medicines. It's not a rights-based understanding. It has to be developed. There are many such things, and we can have a huge discussion on it. Anyway, do you have any other specific questions?

Devaki Nambiar: See, we have all manner of questions, Abhaybhai, but in this kind of approach, it's more about constructing that history. I think for us there is a coalescing or, as you're saying, even the proto-CBMP that is very important to capture, because it very much shapes the turn of events and the design aspects of what ended up happening. Initially what you describe is there, but there is also a design piece that you have now described.

Abhay Shukla: Another design piece was the Jan Sunwais. I told you probably that day that, as Jan Arogya Abhiyan, we had done six Jan Sunwais in Maharashtra in 2004 and each of them was an event. We learnt a lot from those Jan Sunwais. We also had backlash from the health system in some cases. Activists were beaten up in one place. All kinds of things happen. It is not such a simple thing to do a Jan Sunwai. But based on what we understood from those six Jan Sunwai, we were able to use that in CBM's framework in a meaningful way. After that, we did over 600 Jan Sunwais—later called Jan Samvads or public dialogues—in Maharashtra from 2008 to 2015. So, these are different elements. We also have been doing health calendars in Maharashtra. I don't know if you know about the health calendar program. The health

calendar was used to see that the nurses and other ANMs and all would visit the village regularly. Sometimes randomly when they used to come, no person or child would be there to immunize, and when people used to come, they [nurses and ANMs] wouldn't be there. So, we set up a calendar which specified on which day ANM is going to come for immunisation. That would be displayed in the village in a prominent place. And the nurse would be informed that you need to be there on a certain date, like the 12th of November, and if you don't make it, then we'll cross it out and show it in the PHC. And that had a spectacular impact. This was done in Dahanu block of Thane district[60]. This is actually an innovation, which came from a Jan Sangathan: Kashtakari Sanghatana[61]. That is also a form of community monitoring because for the first time in history, people are checking what the health system is doing and what the health care providers are doing, rather than they just coming and giving a bhashan [speech] and going away or just giving some services and going off. So, there were these small precursors which we integrated and upscaled and made the CBM framework. And, of course, Amarjeet Sinha played an important role in that which was not mentioned that day, but that has to be very much acknowledged. He was receptive you see. There're reformers within the system and activists from outside the system who collaborated together to create CBM.

Devaki Nambiar: That's tremendous. And what about score cards? When did that kind of aspect come up? Then we've talked about convergence, even going beyond health, like these additional elements, innovations—however you describe them. Could you talk about how those sorts of, time frame even, of how those emerge?

Abhay Shukla: Yeah, with a bit of exaggeration, one can say a process like community-based accountability, which is working in collaboration with the health system, will either be innovative or it will be nothing. So, it was actually a survival strategy, but it was not just survival. Of course, we managed to continuously expand up to a certain level. But the point was that, when issues were raised from the community level in the first round or first iteration, we realised that some things were getting solved, many things were not getting resolved. Now, once people have been mobilised, we have an obligation to take those issues to some logical conclusion. So, then we innovate and try to see that if it's not working this way, then what can be done? The whole community-based planning framework came from there. Then we realised that, fine, we have picked up the issue locally in Jan Sunwai and they have said they will do something about it, but they haven't. Then we saw that in the RKS, that there is about Rs. 1,75,000 [paune do lakh]. In a PHC, there should be clean drinking water, a chair for waiting patients to sit on, and there are mosquitoes in the wards, so a net needs to be put on the windows. This can be done in Rs. 1,75,000. So, get into the RKS, use that money to solve all these problems which have come up through CBM. So [at] each level, each stage of CBM threw up certain challenges. It threw up certain questions. Some of them were resolved, many of them were not resolved. So then the next round of innovations was to take forward those agendas through some different channels to ensure that they might be better resolved. So, this is one kind of innovation.

The second kind of innovation was related to meeting the backlash and the resistance from the system. When Jan Sunwai initially began, in the first two rounds, there was a lot of resistance from officials, like in Nandurbar[62], the entire Medical Officers Association boycotted the Jan Sunwais. They said, we are not going to come to Jan Sunwais. In Amaravati[63], the first Jan Sunwais which

60 Dahanu is both a town and a Tehsil/Block in Maharashtra's Thane District. Dahanu Block consists of approximately 172 villages. See: https://villageinfo.in/maharashtra/thane/dahanu.html

61 The Kashtakari Sanghatana is an organization that has contributed to the community monitoring of health services in Maharashtra's Thane tribal district. Kashtakari Sanghatana been a part of the community-based monitoring process under NRHM since 2007. It builds capacity among village-level committee members to help them monitor village health services, and it monitors higher-tier institutions, such as rural hospitals. The organization hosts public hearings to provide a forum for community members to highlight shortcomings of health services. It also advocates alongside district health administration. See: https://www.copasah.net/kashtakari-sanghatana.html

62 Nandurbar is an administrative district of northwest Maharashtra, India.

63 Amaravati, located along the Guntur District's River Krishna, is the capital of Indian state Andhra Pradesh.

took place at the district level, the District Health Official initially did not come at all. So, the activists present immediately called the media, they gave the District Health Officer's phone number to the 200-300 people present there and asked them to keep calling until the officer comes for the Jan Sunwai and asked the media to do the same. So, the District Health Officer turned up after 1 hour. Now, there is no set formula for this, but it was a product of the requirement of the situation. So, there are many such things, and there are other types of innovations also [which are] more positive—in the sense of actually working together with grassroots and frontline health workers, interacting with Panchayat members in a more productive way, working with Medical Health Officers at the lower level, decentralised health planning, and trying to understand their [these officers] own problems and help them to solve those problems in a way that would also help people to get better services. A lot of such things have happened. That's a big topic. But I think I've given you some ideas about that. The third kind of innovations are about processes like voluntary CBM. So, voluntary CBM was basically: with the government's money, only so much can happen, and there will be limitations on it so we will just take the mandate from the government and appeal to everybody [in Maharashtra] that if you want to do CBM somewhere, do it. We will give you training, posters, report cards, and material. We will give you a small fund to organise your first Jan Sunwais and rest you can do it by yourself. 30 organisations came up from different parts of Maharashtra and did it. This is something quite remarkable. They have zero honorarium, practically no funds—almost no funds—, and they did it and quite well in some areas. In some of these areas, we later officially expanded CBM.

Devaki Nambiar: So, this seed funding was supported by?

Abhay Shukla: NHM.

Devaki Nambiar: Oh, by NHM only? Okay.

Abhay Shukla: Yes. For this innovation. I think one important part of the story which needs to be told, and which is the more negative part, is that from 2007-08 to roughly 2015, something like eight years, we had a situation in Maharashtra where the civil society coalition—it was a coalition, it was not just SATHI. SATHI was leading the coalition, but it was a coalition. [It] was able to roll out a range of community accountability processes with the endorsement of the state with a reasonable degree of effectiveness and positive impact. After that, national-level changes happened. You know what happened in 2014. State level changes happened in Maharashtra. You might know about that. The state government also changed, and then after 2015, the space for CBM got extremely constricted due to actions from both the national-level government and the state-level government. And not all those actions were deliberate and explicit. So, in government, it's very easy to block things without doing anything. You just don't release the funds. Over. You don't have to take out an order saying, "I'm facing difficulties with CBM" or "CBM is too assertive" or "they're picking up too many issues", nothing. You just start delaying the funds. The release of funds from NHM was delayed so much after 2015-16 onwards every year, that the effective capacity of CSOs at the ground level to organise Jan Sunwais or to organise workshops or to go to villages and do data collection and report card preparation, that got totally constricted. So, that was a start, and then gradually taking over the entire leadership also, instead of being a joint leadership or a substantial role for CSOs, the leadership also gets taken over by government and government-associated agencies. So, our scope for manoeuvre reduced so much. We joke about how under the leadership of Modi ji, [the fact that] we were able to do so much itself is a big deal.

Devaki Nambiar: Well and what do you make, Abhay bhai, of the Finance Commission[43]? So, there is civil society and political society. Now there are also two, in the sense, in the analysis of Partha Chatterjee. So, this seems to be pushing into LSG and political society, that kind of model. I'm sort of saying too much, but this is my sense of it. But what is the prognosis in light of these most recent developments in your analysis?

Abhay Shukla: That's a big topic that we can talk

about for another half hour. But actually, I have a presentation which I had made in AGCA recently in which I talked about reimagining community action for health in the light of COVID-19. I basically said that something paradoxical has happened during COVID-19 as far as community action is concerned. So, we had a situation. At least this definitely happened in Maharashtra, but in my understanding, [it] also happened in, at least some other states, perhaps many other states. So, there is a simultaneous absolute constriction of participation at the national level (hyper-centralisation), and also in most states, state governments also moved into a military mode, and the State Health Department, State Health Minister, Health Secretary, and maybe a few other close circle officials essentially took over all decision making and blocked all other kind of inputs. So, there is a hyper-centralisation of decision making and action at the national and state levels, but at the local level, there was a huge proliferation of collaborative action in many places, and this was despite the state—not because of the state. So, we saw local PRI members, local medical officers, PHC medical officers, ANMs, ASHAs reaching out to communities, asking people to help collaborate in setting up isolation centres, community COVID care centres, giving food to people and providing other kinds of support. All kinds of things happen, and that is completely spontaneous. Yeah, this interesting paradox tells us something, about both the potential of Indian society and limitations of the Indian state. You asked about civil and political society, right? In India, both function. Anything that is true for India, the opposite is also true.

So, we have a state which functions in a certain kind of bureaucratic, top-down, hierarchical, and in a certain way, quite constrictive manner. But on the other hand, our society is not dead yet and we saw that during COVID. You know what Diogenes told Alexander the Great? You know the story of Diogenes and Alexander the Great? Diogenes was kind of a sage who used to live with very bare minimum things, and he was completely away from any sort of material comfort. Alexander the Great went to meet him and he said, “Diogenes, tell me what you want. I can give you anything. I’m Alexander.” He said, “Yes, move and make way for the sunlight.”

So, just make way, and the state just needs to get out of the way and allow the due resources. Even [if] the resources of 14th Finance Commission and the 15th Finance Commission that are supposed to reach the Gram Panchayat are properly given, then a lot of things can be done. So, I think that is how we need to restructure and resurrect community action for health, making use of the collaboration, the social capital, as well as resources which are both actually and potentially available at the village level, which will require, of course, many other things to be done. But there is a potential for that and there will be a lot of diversity across states. Tamil Nadu is developing a Right to Health policy; Rajasthan is developing a Right to Healthcare Act. So, it’s not like everything is over everywhere. What’s happening at the national level is visible to you better than me, so let’s not comment on that, but that doesn’t mean that things will not happen. As I said, if things don’t happen because of the state, they will happen despite the state. That’s what now we’re trying to do in Maharashtra through our Right to Healthcare campaign.

Devaki Nambiar: And when we’re thinking about the Right, I mean, I have so many thoughts, but let me just try to be clear. You said that COVID represents the kind of moment in a big [way], [and] this paradox has emerged. Do you think in light of COVID but also, you know, epidemiological shifts and so on, and prior experience encounters with the health system, the CBMP experience—have peoples’ expectations and areas of focus within the health system changed? Have their needs and demands in what we have, those changed or not changed? Newer generations, you know. What does that look like, in your sense?

Abhay Shukla: That’s a little difficult to generalise about, it’s very contextual. In different situations, it’s different, because in Tamil Nadu or Kerala, what people expect from a PHC is very different from what people expect from a PHC in Bihar. You probably might be aware of the situation in places, I mean, in Maharashtra also, it will be on a little different kind of plane. But what happened in COVID is that people realised the importance

of public health services like never before in the pandemic. They also recognised the value of frontline healthcare workers from the public health system. I think they also energised people to come together and collaborate with grassroots level public health services in many places, despite problems. I'm not saying there were no problems. I mean, there was discrimination, there was branding, there was forced vaccination, all kinds of [things and] we know all those stories. But despite all of that, on the whole, it brought people at least temporarily, a bit more closer to the grassroots level or frontline level public health services in a manner which at least opens spaces for reimagining health services. If there is a social action, take that discourse forward. That is what we have been trying to do in Maharashtra, through the Right to Healthcare campaign and also the second thing which happened is the exposure of the private healthcare sector. Huge overcharging and all the other kinds of irrationalities and rights violations which took place have probably opened up the eyes not only of people in poor communities, but even the middle class about the nature of the private health care sector and the need for change on that front.

Devaki Nambiar: Yes. Okay.

Abhay Shukla: So, maybe we can wind up. I now have another meeting at five.

Devaki Nambiar: Okay. All right. No, I don't have a dhamakedar [Steamy] question. Misimi, do you? We have just minutes remaining with Abhay bhai.

Misimi Kakoti: No Devaki, I think we have covered, and I had a question which I had already asked in the beginning.

Devaki Nambiar: Abhay bhai, this might be like homework, but we will share this whole transcript with you, just for you to confirm it and approve it. So, you may need to just skim that and make sure there's anything we need to check on. But really, thank you so much. You're right. This really needs to be a sort of a quilt of just oral history that's done again and again. So, let's see if we can figure out how to do that. But anyway, we're very grateful for your time.

Abhay Shukla: A couple of other things, which I wanted to say just very briefly. So, when people ask us about the impact of CBMP, okay, I tell them two things. First of all, I tell them CBMP is a god of small things. So, you don't have dramatic policy changes. You don't have major visible changes in the way in which state level health policy is reshaped. But we have hundreds of stories of change at the grassroot level, which is very difficult to communicate to an outsider. If you ask me to explain it, I'll have to ask you to spend two hours with me, or travel with me to a village, or talk to four grassroots activists—then you'll understand what CBM has done.

Devaki Nambiar: So, it's like even if you have a policy change, but if it actually got implemented in ground reality, that is still a question, right? Is that perhaps the ultimate aim we always want to have? If that change at the ground level be these small things?

Abhay Shukla: Second thing is... Yes, it's a god of small things. Second thing is that it's all about opening up a closed system of power and sharing of power.

Devaki Nambiar: Right, yes.

Abhay Shukla: So, I remember asking some activists in Amravati about what did the CBM do there, so they said that before when we used to go to a PHC, the doctor used to make us stand outside, but since CBM has begun and we've had Jan Sunwai [and] we have made report cards, the doctor now makes us sit inside and offers us tea. This won't come in your PIP as an indicator, but for grassroots activists, it's a change in power. That's what he's saying in his own language. There's some equalisation of power, and that is critical and not just [for] the activists, but even people from the village. If they go and talk, then they're listened to. So, these are the changes. It's an extremely multilevel, complex system of power, as you know. In that it's at least beginning to equalise the power relationships at the grassroots level. That is something that we at least attempted, and to some extent managed to initiate, I will say, in Maharashtra. And third, is that in the light of the COVID epidemic, co-production of health is very clearly the way forward. This is something that I

have presented in AGCA also. COVID has shown us health cannot be produced by governments, and it cannot be produced by communities on their own. It has to be co-produced. Co-production is completely impossible unless there is an equitable collaboration. That is what community action for health should be about. So, whether it is vaccination, or quarantine, or isolation, or contact tracing, or testing or anything. Without co-production, which among these are possible? If there’s no co-production, nothing will be done properly. It will be some stretched out version but with co-production everything can happen at its optimal level. At least now we should wake up to the need for co-production of health, and that should kind of guide the further evolution and design of community action for us.

Devaki Nambiar: At all levels, really.

Abhay Shukla: Yes, at all levels.

Devaki Nambiar: Gosh. Thank you. That’s really superb.

Transcript ends.

Responses from S. Jalaja

S. Jalaja was unable to attend the Witness Seminars and asked the team to send the relevant questions to her. The below questions were sent to her.

1. How did the community accountability mechanisms fit in with the original design of the NRHM? In your role, how did you picture/envision the relationship of this aspect to the broader design and ambition of NRHM? Relatedly, who were the (other) main champions in government?
2. What would you say the efforts/experiments with institutionalising community action for health in NRHM achieved? What in turn were the areas where we have failed or need to keep pushing?

S. Jalaja's written responses

How did the community accountability mechanisms fit in with the original design of the NRHM?

I recall that, as the first Mission Director, NRHM, within the overall design of the NRHM, accountability of the community was to be ensured by strict monitoring and close interactions through several mechanisms, including ASHA, Panchayats, civil society (including NGOs, trusts, private Institutions, independent organizations like NHRC, and other 'rights' bodies at the national and state levels). Moreover, it envisaged monitoring by Central and State Planning Commissions, mechanisms like the State /District Health Societies, independent evaluation by teams comprising of NGOs/public health experts, field visits by officials and non-officials, regular monitoring by Ministries and Departments/ involvement of the media, etc.

Efforts/experiments with institutionalizing community action for health in NRHM- Achievements; the areas of failure or those need to be pushed?

Efforts were made, in the initial stages, to involve the Civil Society and ensure public accountability through various mechanisms mentioned above. However, as the mission (NRHM) progressed, the focus appears to have shifted to its implementation, confined mostly to government agencies, that too as a routine government program. Health and health determinants (drinking water, nutrition, sanitation, environmental matters) are handled by different ministries and agencies as vertical programs. Hence, coordination at different levels was difficult. The Panchayat Raj institutions were neither appropriately empowered, nor provided human and financial resources to assist NRHM. Involvement of the civil society waned due to various reasons. The engagement of 'rights' bodies also diminished over time. State- and district-level societies were either non-functional or carried on routinely. I visited over hundred districts in India as a Special Rapporteur of NHRC (2012-2018) and in this process I visited health facilities and reviewed implementation of the mission. Over a period of time, community participation or measures for ensuring community accountability were hardly visible. Appropriate action to be taken in this regard is too evident from the initial design of NRHM.

Notes

HeaL

Health equity action Lab

The George Institute
for Global Health

@GeorgeInstIN
www.georgeinstitute.org.in
@GeorgeInstIN
