# Supplementary material for: How communitization begets and endures sarkarikaran: a witnessed history of community action for health in India’s national rural health mission
Source: BMC Health Serv Res. 2025 Jul 8;25:940. doi: 10.1186/s12913-025-13058-0 (PMC12235939; doi:10.1186/s12913-025-13058-0)
Supplement: Supplementary file 2 — Supplementary Material 2. [file 12913_2025_13058_MOESM2_ESM.pdf]

Witness Seminar on Community Action for Health in India

'Communitization' and community-based accountability mechanisms under the National Rural Health Mission (NRHM)

Second of Two Witness Seminars

Held online via zoom on 10th December 2021

Health equity action Lab

The George Institute for Global Health

5

Contents

Instructions for Citation 3

Acronyms 3

Background and Purpose 4

Community participation in health in India – key antecedents 4

The community-based accountability mechanisms under the National Rural Health Mission (NRHM) 4

References 5

Witness Biographies 6

Proceedings of the Witness Seminar 8

Annexure 29

In-depth Interview with Dr. Mirai Chatterjee 29

In-depth Interview with Dr. Thelma Narayan 40

Edited by

Misimi Kakoti, Siddharth Srivastava, Shraddha Mishra, Gloria Benny, Hari Sankar, Devaki Nambiar

Publishing

First published in 2022 by
The George Institute for Global Health | INDIA
308, Third Floor, Elegance Tower
Plot No. 8, Jasola District Centre
New Delhi 110025 | India
T +91 11 4158 8091-93 | F +91 11 4158 8090
www.georgeinstitute.org.in

In support of an ongoing research collaboration with the Civil Society Engagement Mechanism (CSEM) for UHC2030 globally, the George Institute for Global Health India conducted Witness Seminars to document community action and social participation for health in India using internal funds.

All rights reserved. This material is made available for use for personal research and study. We give permission for entire files to be downloaded to your computer for such personal use only. For reproduction or further distribution of all or part of the file (except as constitutes fair dealing), permission must be sought from the editors.

Download

This document is available to download as a free PDF and in other formats at:
www.georgeinstitute.org/witness-seminar-reports

Acknowledgements

We would like to acknowledge Shweta Marathe, Indira Chakravarthi, Rakhal Gaitonde and Rajani Ved for their guidance at the early stages of this project. Ms. Rajani Tatineni provided support for the organisation of the Witness Seminar as well as preparatory meetings. Mr Jason Dass and Mr Alexander Baldock helped with graphics and design. We are also grateful to the team at Auxohub, led by Yashasvini and Vandana, for their careful work on the transcriptions.

Instructions for Citation

If you are using this document in your own writing, our preferred citation is:

Please cite the publication as a whole as: Misimi Kakoti, Siddharth Srivastava, Shraddha Mishra, Gloria Benny, Hari Sankar, Devaki Nambiar (eds), 'Communitization' and community-based accountability mechanisms under the National Rural Health Mission (NRHM). Transcript of a Witness Seminar. (New Delhi: The George Institute for Global Health India, 2022).

References to direct quotations from this Witness Seminar should follow the format below:

[Witness name], in the 'Communitization' and community-based accountability mechanisms under the National Rural Health Mission (NRHM). Transcript of a Witness Seminar, held 10 December 2021, (New Delhi: The George Institute for Global Health India, 2022) www.georgeinstitute.org/witness-seminar-reports, [page number of reference].

Acronyms

**AGCA**	Advisory Group on Community Action
**ANM**	Auxiliary Nurse Midwife
**ASHA**	Accredited Social Health Activist
**BDO**	Block Development Officer
**CAH**	Community Action for Health
**CBM**	Community Based Monitoring
**CBMP**	Community-based Monitoring and Planning
**CBO**	Community Based Organisation
**CEHAT**	Centre for Enquiry into Health and Allied Themes
**CHAD**	Community Health and Development
**CHAI**	Catholic Health Association of India
**CHC**	Community Health Centre
**CHV**	Community Health Volunteer
**CMO**	Chief Medical Officer
**COVID**	Coronavirus Disease
**CSDH**	[World Health Organization's] Commission on the Social Determinants of Health
**CSO**	Civil Society Organisation
**DG**	Director General
**FRCH**	Foundation for Research in Community Health
**GOI**	Government of India
**HR**	Human Resources
**IAS**	Indian Administrative Services
**ICMR**	Indian Council of Medical Research
**ICPD**	International Conference on Population and Development
**ICSSR**	Indian Council of Social Sciences Research
**INA**	Indian National Army
**IT**	Information Technology
**JSA**	Jan Swasthya Abhiyan
**LGBTQI**	Lesbian Gay Bisexual Transgender Queer Intersex
**MFC**	Medico Friend Circle
**MGNREGA**	Mahatma Gandhi National Rural Employment Guarantee Act
**MLA**	Member of Legislative Assembly
**MO**	Medical Officer
**MoHFW**	Ministry of Health and Family Welfare
**MoU**	Memorandum of Understanding
**NCD**	Non Communicable Disease
**NGO**	Non Governmental Organisation
**NHM**	National Health Mission
**NHRC**	National Human Rights Commission
**NRHM**	National Rural Health Mission
**NTI**	National Tuberculosis Institute
**NUHM**	National Urban Health Mission
**OPD**	Out Patient Department
**OT**	Operation Theatre
**PHC**	Primary Health Centre
**PHM**	People's Health Movement
**PMJAY**	Pradhan Mantri Jan Arogya Yojana
**PMO**	Prime Minister's Office
**RCH**	Reproductive and Child Health
**RKS**	Rogi Kalyan Samiti
**RSBY**	Rashtriya Swasthya Bima Yojana
**SEARO**	(WHO) South-East Asia Regional Office
**SEWA**	Self Employed Women's Association
**SHRC**	State Health Resource Centre
**SOCHARA**	Society for Community Health Awareness Research and Action
**TB**	Tuberculosis
**UNFPA**	United Nations Population Fund
**UNICEF**	United Nations Children's Fund
**UPA**	United Progressive Alliance
**VHM**	Village Health Nurse
**VHSNC**	Village Health Sanitation and Nutrition Committee
**WHO**	World Health Organisation

Background and Purpose

Community participation in health in India – key antecedents[a]

Various global developments, including the Alma Ata declaration, the establishment of the People's Health Movement in 2000, and the International Conference on Population and Development (ICPD), have shaped the discourse around social participation in health. More broadly, the geopolitical context of Non-Aligned Movement, the New International Economic Order, and attempts to create an alternative paradigm for global development have centre-staged social participation, redistribution of power, and a rights-based approach for health.

Such has also been the case in India, where community participation in health and health reform precedes Independence. A range of individuals, institutions, and collectives set the stage for community action for health.[1] Building on these was the National Rural Health Mission (NRHM), launched in 2005 and widely lauded as a major health policy achievement, particularly for its emphasis on the role of community participation, and for resulting in major gains in India's advancement with the Millennium Development Goals.[2] NRHM created several institutional arrangements for community ownership and leadership in health. These included one of the world's largest community health worker programs, village- and facility-level committees with delegated financial powers, community monitoring, an action group tasked with supporting community action nationwide, and more.[3, 4]

NRHM itself was designed to promote bureaucratic or programmatic decentralization in the health sector: decentralization of funds, functions, and functionaries to subnational government levels were part of the operational framework.[5] NRHM also recognized the importance of decentralization and district management of health programs, conceiving the district[b] as the core unit of planning, budgeting, and implementation.[6] In each state or union territory of India, however, existing contexts, path-dependent processes, and stakeholders were imbricated in the 'communitization' process in unique ways. We sought to understand these processes and history at the national and state levels using the Witness Seminar methodology.

Our methodological appendix is provided on our project landing page.

The community-based accountability mechanisms under the National Rural Health Mission (NRHM)[c]

Globally, since the 1990s, community participation has been increasingly linked to health systems accountability and governance. This is also reflected in NRHM's tenets, wherein it is recognised that the achievement of Health for All is possible "only when the community is sufficiently empowered to take leadership in health matters".[5] This policy thought is translated into interventions in the form of the Community Based Monitoring and Planning (CBMP) processes under NRHM.

a. This section is reproduced in each of five Witness Seminars that were carried out in 2021 with a focus on community participation in NRHM.

b. In the Indian administrative scenario, the nation is subdivided into states, and each state is further divided into districts. The districts are then made into smaller subdivisions of village and blocks in rural areas, and urban local bodies exist in urban areas.

c. This section is reproduced in each of two Witness Seminars that were carried out in 2021 with a national-level focus on community participation in NRHM.

The CBMP pilot was launched in 2007-08 by the Government of India and includes initiatives such as the Village Health Sanitation and Nutrition Committees (VHSNCs)[d], Village Report Cards[e], and Jan Samwads[f], among others. These were developed and implemented with involvement of NGOs, resource institutions and local communities; and the ASHA Mentoring Group, the Advisory Group on Community Action, and the Regional Resource Centres offering inputs to facilitate the process.[5] The evaluation of the CBMP pilot in 2008 reported improvements in health services from community-based monitoring in the states. Some major highlights from the evaluation include: VHSNCs' enhancement of knowledge on rights and entitlements in the community, the Jam Samwads' leading communities to demand better services, and an active engagement between the community and health departments.[6] CBMP–later renamed Community Action for Health (CAH)–was scaled up to cover more states from 2009 onwards.

.Universal Health Coverage (UHC) has started occupying greater prominence in India's policy aspirations since 2010 and with the launch of the Ayushman Bharat programme. Community-based accountability and participatory governance of health systems are recognised as key elements for UHC.[7] The civil society has a crucial role in facilitating such accountability mechanisms in collaboration with the Government.[7] Since the National Health Policy of 2017 grants weightage to the role of the private sector in achieving UHC, the role of community-led accountability has become important to protect patients' interests and rights. As policy resolutions of the Government centred around UHC and community action in health have been continuous in civil society partnerships since 2019, there is scope for understanding if and how existing community accountability mechanisms–modelled under NRHM–can be better leveraged to advance UHC commitments. Thus, we sought to deeply understand how CBMP structures have waxed and waned since their emergence in 2005. We organised a series of two Witness Seminars to document the provenance, features, achievements, challenges and lessons learnt from NRHM's CBMP/CAH processes.

References

1. Prachitha J, Dhume A, Subramanian S. India in Pursuit of Millennium Development Goals: Were the Targets Really Feasible? J Dev Soc. 2019 Mar 1;35(1):105–33.
2 Gaitonde R, San Sebastian M, Muraleedharan VR, Hurtig A-K. Community Action for Health in India's National Rural Health Mission: One policy, many paths. Soc Sci Med. 2017 Sep 1;188:82–90.
3 Seshadri SR, Parab S, Kotte S, Latha N, Subbiah K. Decentralization and decision space in the health sector: a case study from Karnataka, India. Health Policy Plan. 2016 Mar;31(2):171–81.
4 Raut M, Sekher TV. Decentralization of Health Care Systems: Findings from Odisha and Gujarat, India. J Health Manag. 2013 Jul 24;15:235–51.
5 Ministry of Health and Family Welfare. Framework of Implementation National Health Mission 2012-17 [Internet]. New Delhi: MoHFW; 2012 [cited 2020 Oct 22]. 59 p. Available from: https://nhm.gov.in/New_Updates_2018/NHM/NHM_Framework_for_Implementation__08-01-2014_.pdf
6 Singh S, Das A, Sharma S. Reviving Hopes, Realising rights- A report on the first phase of community monitiring of NRHM [Internet]. New Delhi: CHSJ; 2010. Available from: https://nrhm-communityaction.org/wp-content/uploads/2017/06/A_report_on_the_First_phase_of_Community_Monitoring.pdf
7 Lahariya C, Roy B, Shukla A, Chatterjee M, Graeve HD, Jhalani M, et al. Community action for health in India: evolution, lessons learnt and ways forward to achieve universal health coverage. WHO South-East Asia J Public Health. 2020 Apr;9(1):82–91.

d. Village Health Sanitation and Nutrition Committee (VHSNC) is a key institution introduced under NRHM to facilitate community participation in supporting, implementing, and monitoring health projects. It is formed at the level of the revenue village, and if the population of the revenue village is more than 4000, it can be formed at the level of a Ward Panchayat as it is in Kerala. From Government of India. (n.d.). Handbook for members of Village Health Nutrition and Sanitation Committee. Ministry of Health and Family Welfare.

e. Some community action for health projects under NRHM use report cards to collect information from community members, such as for the monitoring of service quality at local health facilities. See: https://nrhmcommunityaction.org/wp-content/uploads/2016/11/Community_-Action_for_Health.pdf

f. Jan Samwads are public dialogues through which community members can share feedback on health services under the National Health Mission (NHM), which encompasses both NRHM and the National Urban Health Mission (NUHM). For more information, see: https://nrhmcommunityaction.org/bridging-the-digital-divide-connecting-communities-with-health-systems-through-virtual-jan-samwad/

Witness Biographies

Note: Biography information reflects the position of witnesses at the time of the seminar. Some designations and/or roles may have changed.

CHAIR:

Professor Rama V. Baru

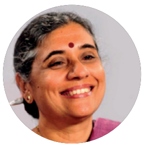

Professor Rama Baru is Professor at the Centre of Social Medicine and Community Health, Jawaharlal Nehru University, and an Honorary Fellow, Institute of Chinese Studies, Delhi, India. She is also an Honorary Professor at the India Studies Centre, Central China Normal University, Wuhan, China. Her major areas of research work include infectious diseases, comparative health systems, commercialisation of health services, and health inequalities. She is a member of the Ethics Committee at the All India Institute of Medical Sciences, the Technical Appraisal Committee for Health Technology Assessment, Department of Health Research at the Ministry of Health and Family Welfare (MoHFW), and the Scientific Advisory Group, Indian Council of Medical Research, New Delhi.

WITNESSES:

Dr. Prabir Chatterjee

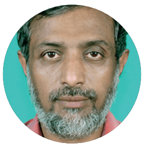

Dr. Prabir Chatterjee is a medical doctor and a community health specialist who is also former Executive Director of the State Health Resource Centre (SHRC) in Chhattisgarh. His extensive work in the field of community health includes work with tuberculosis (TB) patients in Hiranpur. with the World Health Organisation in Godda, Jharkhand, and with the UNICEF in Raiganj, West Bengal. He was involved in providing technical guidance in the implementation of Chhattisgarh's Mitanin Programme. He was also a member of the National ASHA Mentoring Group of the National Health Mission (NHM).

Dr. Nerges Mistry

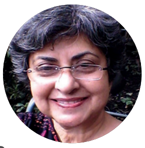

Dr. Nerges Mistry is Director and Trustee of the Foundation for Medical Research, Mumbai and the Foundation for Research in Community Health, Pune. She is a member of the Mumbai Alliance Against Tuberculosis and has participated in several advocacy measures in the recent past. In the field of community medicine, she has had experience in the development of a community-based health care system, and intervention research in the management of drinking water in rural communities. She is also a member of the National ASHA Mentoring Group of the NHM since its inception, and has been on the Advisory Group for Community Processes and Traditional Medicine for the 11th and 12th Five-Year Plans, Government of India. She serves as an Expert on the Global Coalition Against Tuberculosis (GCAT), is an Advisor to the TB PPM Learning Network and was also a member of the National Technical Expert Group on Diagnosis of TB under NTEP.

Dr. Thelma Narayan

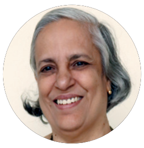

Dr Thelma Narayan is an epidemiologist, health policy analyst and an activist who has been the Director, SOCHARA School of Public Health Equity and Action (SOPHEA). She is a member of the Advisory Group on Community Action of the NHM. She is also currently a member if the Lancet Citizen's Commission on Reimagining India's Health System to reach Universal Health Care. She was also a member of the Task Force in Health and Family Welfare, Karnataka, and has been involved in evolving public health and primary healthcare-oriented state health policies in Karnataka and Odisha. She has also been a member of NHM's National ASHA Mentoring Group.

Dr. Rakhal Gaitonde

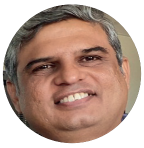

Dr Rakhal Gaitonde is a Professor at the Sree Chitra Tirunal Institute for Medical Sciences and Technology, Trivandrum, Kerala. His work largely focuses on health policy, the social determinants of health, and health systems. He was involved in setting up a people-controlled health system in Maharashtra with Foundation for Research in Community Health. He has been involved with the Society for Community Health Awareness Research and Action (SOCHARA), where he was involved in the implementation of the NRHM in Tamil Nadu. He is also a member of the National ASHA Mentoring Group of the NHM.

Dr T. Sundararaman

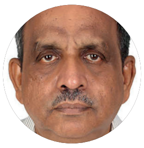

Dr. T. Sundararaman is former Global Coordinator of the People’s Health Movement (PHM), visiting faculty at Jawaharlal Nehru University and former Executive Director of the National Health Systems Resource Centre, New Delhi. He was also the Director of the State Health Resource Centre in Chhattisgarh, providing technical guidance to the launch of a major community health worker programme (the Mitanin programme). He has formerly served as the Dean of the School of Health Systems Studies at the Tata Institute of Social Sciences. Dr. Sundararaman has been actively involved with health and education movements in India and a number of key policy streams on health systems strengthening at the national and sub-national level. He played a pioneering role in formation of the Jan Swasthya Abhiyan (JSA) and is a founding member of the All India People’s Science Network (AIPSN).

Dr. Mirai Chatterjee (interview only)

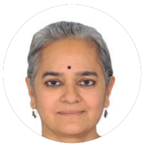

Mirai Chatterjee is the Director of the Social Security Unit at the Self-Employeed Women’s Association (SEWA). She is also serving as the Managing Trustee of the Lok Swasthya SEWA Trust and is currently the Chairperson of the SEWA Cooperative Federation. Mirai is a founder of the Lok Swasthya Health Cooperative and the National Insurance VimoSEWA Cooperative Lts, She serves on the Boards of several organisations including PRADAN and Save the Children and PRADAN. She is also Chairperson of Women in Informal Employment Globalising and Organising (WIEGO).She has been working at the forefront of improving health and financial security of women working in India’s informal economy. She is a member of the Advisory Group on Community Action of the National Rural Health Mission and was a Commissioner in the World Health Organization’s Commission on the Social Determinants of Health. She was also a member of the High-Level Expert Group on Universal Health Coverage set up by the Planning Commission of India in 2010 and the National Advisory Council (NAC) set up by the Prime Minister of India in 2010.

Proceedings of the Witness Seminar

Proceedings start.

Devaki Nambiar: Thank you all for making the time to be here. This is the second series of national Witness Seminars, focused on community participation and health in the country. We wanted to place emphasis on community accountability mechanisms under NRHM, and the NRHM process which you all were part of. We have had conversations with all of you to understand the trajectory, even understand how to tell the story of the trajectory of community participation and health as sought to be institutionalised by the NRHM. Thank you for making time. It has been rich having pre-conversations with each of you. We are going to tell the story with each of your experiences. This meeting is being recorded; thank you for giving your consent.

Quickly, we will be going through a few questions. Our chair Professor Rama Baru from Centre for Social Medicine and Community Health at Jawaharlal Nehru University will be chairing the session and steering us through this conversation. We will be transcribing the session and sharing it with you all. You will have an opportunity to look at the transcript, add to it, indicate to us where we need more annotations and references. Then, we will be going through a process of annotating. The intention of this exercise is to create an archive for public record of what happened in this process, and allow this to be used for further research, academic and writing purposes and so on. We are trying to identify archives where this could actually go. We haven't had much luck with national archives, but we will think of places where this can be disseminated and used as part of living memories and documentation of important initiatives in India around community participation. We have one regret: Jhalani Sir had something come up and he will not be able to attend. I shall hand over to Professor Rama Baru to take us through the proceedings and perhaps deliver some opening remarks to get us started. Over to you, Professor Rama.

Rama Baru: Thank you, Devaki and team, for persuading me to chair this event. I agreed because it is an important way of archiving memories, both individual and institutional. People present here represent both. There are also important people who were architects and actors in this process who are now no longer with us. The first name that comes to my mind is Dr. Antia's[1], and Mr. Gopalakrishnan's[2], who was in the PMO [Prime Minister's Office]. I think it is very important for us to go through this exercise. Each of you have been involved in People's Health Movement. You have been important players in the pre-NRHM [era], changing the course of NRHM, especially bringing in the idea of communitization and also a lot of NGO experiences over the years, distilled through this process.

Without taking much time, I think Devaki has shared a number of questions and domains which each of you wish to cover. There will be overlaps. If anybody wants to come in and supplement points made by anyone else, raise your hand and we will give you the opportunity. Each of you will be given 10 minutes to make your presentation. We will then have adequate time for some interjections and further discussions.

May I first invite Dr. Prabir? Please come in, thank you.

Prabir Chatterjee: I thought I would cover 10 points, so that is nice for the 10 minutes. I will start a little before the start of the process. Not something I have witnessed myself; I was outside 'til the middle of the process. The first thing I am describing is the Dalli-Rajhara women's section—Dalli Rajhara women's section of the Chhattisgarh Mukti

1 Dr. Noshir H, Antia was one of the key people advising the consolidation of the National Rural Health Mission (NRHM). He played a key role in carving out space for the civil society in the process of shaping the programme. See: www.fmrindia.org/founder

2 R. Gopalakrishnan was an India Administrative Service (IAS) officer who had served as a Joint Secretary and an Additional Secretary at the Prime Minister's Office (PMO). He was involved with the launch of the NRHM.

Morcha[3], where the women there decided to take up the question of alcohol[4]. When they took up the question of alcohol, they took it up in a campaign mode. That was one of the early examples of what later got pulled into the Mitanin[5] program. The second example is from Bilaspur. I did not know this at that time, but later when I was reading Aruna Roy's book[6] on the Right to Information Act[7] and how it came about, there were at least two or three major meetings that took place at Bilaspur with the support of Harsh Mander[8]. These are two things completely outside the NGO field; one is a set of IAS [Indian Administrative Service] officers who felt that something should be done to give information to people, and the other one came from a union perspective where they thought that social problems could be addressed in Sangha [assembly] type of mode. These are two things that fed into the later examples of communitization in Chhattisgarh in the Mitanin Program. One thing that is very clear is that the selection of Mitanin trainers is written into the NRHM[9] and selection of the ASHA[10] workers. However, it is not practised very much outside of Chhattisgarh. During the selection of the Mitanin trainers, it was the NGO workers who came in, and nowadays it has been institutionalised.

Firstly, a Mitanin trainer from outside comes to a new area and they talk to the villagers in the mohalla [locality] for some time and they explain what the health work is about and what sort of person they are looking for. Secondly, they come back after two weeks and ask the community, "Do you think there are any women who could fit in this kind of process, who would be able to participate, who would be able to look after other people's health and bring up issues?" Finally, in the third meeting, they come back and ask, "Have you finalised which person?" and also form a group of women who later become the Mahila Aarogya Samiti[11] or the Village Health and Nutrition Committee[12]. This group of women select one among them to lead the day-to-day work in the area. This process did not take place in other states. I remember in West Bengal when they brought it, they took up two blocks in each of the three districts in 2006 or 2007 or maybe later. They said,

3 The Chhattisgarh Mukti Morcha emerged out of a trade-union movement in Dalli-Rajhara region of Chhattisgarh. It took up a resistance around issues of wages of mine workers and other economic rights-related demand. It also mobilised people around anti-liquor campaigns (see note 4). See: sanhati.com/shankar-guha-niyogi-archives/

4 The Mahila Mukti Morcha mobilised the anti-liquor campaign, wherein the women members organised "rallies and [fought] for the closure of liquor shops close to plants, factories and villages", as they felt domestic violence inflicted on them by men stemmed from their consumption of alcohol. See: Dash DK. Diverse and Contra-Sectional Subjectivities in Social Movement Unionism: A Study of Chhattisgarh Mukti Morcha (Mazdoor Karyakarta Samiti). Sociological Bulletin. 2021 Jan 1;70(1):76–93.

5 The Mitanin Programme, established by the Chhattisgarh government in 2002, consists of community health workers (CHW) who "go beyond health-programme specific interventions to embrace community mobilization and action on local priorities." See: Nandi S, Schneider H. Addressing the social determinants of health: a case study from the Mitanin (community health worker) programme in India. Health Policy and Planning. 2014 Sep 1;29(suppl_2):ii71–81. www.academic.oup.com/heapol/article/29/suppl_2/ii71/587209?login=true; Nambiar D, Sheikh K. (2016). How a Technical Agency Helped Scale Up a Community Health Worker Program: An Exploratory Study in Chhattisgarh State, India. Health Systems and Reform. 2(2):123–134. DOI: 10.1080/23288604.2016.1148802

6 Dr. Prabir referred to: Roy A, Collective M, Gandhi G. The RTI Story: Power to the People. First edition. New Delhi: Roli Books; 2018. 424 p.

7 The Right to Information Act was enacted in 2005 as a step to ensure accountability and transparency in the functioning of the government by mandating timely response to the public's requests for information from any government body or institution. See: www.rti.gov.in/aboutrti.asp

8 Dr. Harsh Mander is an Indian author, columnist, researcher, teacher, and social activist. He was Director of the Centre for Equity Studies, a rights-based research organisation based in New Delhi and also served as Special Commissioner to the Supreme Court of India in the Right to Food Campaign. Mr. Mander was a member of the National Advisory Council of the Government of India, set up under the government around the same time NRHM was launched. See: www.argumentativeindians.com/harshmander

9 The National Rural Health Mission (NRHM) was a centrally sponsored scheme of the Government of India launched in 2005 to provide affordable, equitable and quality health care to the rural population. The thrust of the scheme has been on setting up a community-owned and decentralized healthcare delivery system with inter-sectoral convergence to address determinants of health such as water, sanitation, education, nutrition, and gender equality. It is now integrated under the overarching National Health Mission (NHM) since 2013 alongside the National Urban Health Mission (NUHM). See Government of India (n.d.). National Rural Health Mission: Framework for Implementation (2005-12). Ministry of Health & Family Welfare. Retrieved from www.nhm.gov.in/WriteReadData/l892s/nrhm-framework-latest.pdf.

10 One of the key interventions under NRHM is to provide every village in the country with a trained female 'health activist' i.e., the Accredited Social Health Activist (ASHA). ASHAs are trained to work as an interface between the community and the public health system. See: www.nhm.gov.in/index1.php?lang=1&level=1&sublinkid=150&lid=226

11 Mahila Arogya Samiti (MAS) is a key intervention under the National Health Mission (NHM). It is a "local women's collective with elected Chairperson and Secretary" to "[address] local issues related to Health, Nutrition, Water, Sanitation, and [other] social determinants of health". See: www.nhm.gov.in/images/pdf/NUHM/Training-Module/Mahila_Arogya_Samiti.pdf

12 Village Health Sanitation and Nutrition Committee (VHSNC) is a key intervention introduced under NRHM to facilitate community participation in supporting, implementing, and monitoring of health projects. Read more here.

"We are going to try this ASHA thing out in a non-BIMARU[13] state." So, they took two blocks in our district, which was always at the bottom of the statistics in West Bengal, and they selected women.

After a month (or more)[14] of training given by an NGO, I was called to attend the graduation meeting as a UNICEF staff member in that area. I asked them a question: "Suppose there is a river. On one side is the village and on the other side is the medical system/CMO/medical centre, and you are the ferry boat person connecting the two sides—the village and the medical system. Then where do you belong?" All 12 or 13 people's answer was very clear. They said, "We belong on the hospital side." I was so upset that I made a comment in Bengali shraddhobari hoiye geche[15]. This was not an "Annoprashon" or first rice of the program; this was the death knell of the program. If the person says by the end of the training that he had become a part of the health system, then the whole program has failed. They are not a part of the community.

However, I was wrong. Even though large amounts of selection in other states were based on marks one got or what the BDO [Block Development Officer][16] said, finally we found that the large number of ASHA workers have sided with the people. Even though they did not say in the beginning, theoretically they thought that they had been selected because they had paid a bribe or whatever. In different states, they had different reasons. Finally, the ASHA karmis [workers], like the Mitanins, have taken the side of the community and have brought up community issues.

Going back to Chhattisgarh, I was looking at some of the old documents of SHRC[17]. I noticed [that], in Chhattisgarh, the Swasthya Panchayat Yojana[18] was functioning in 2006. At that time, most of the country did not have ASHA karmis. The idea that a Panchayat[19] has to be healthy, and the interface between health and Panchayat, was already there in Chhattisgarh. Large number of those were in campaign mode those days, and they developed a small questionnaire which I can see being used even now at Mahila Arogya Samiti, in Chhattisgarh, even at all-India level. It had 27 questions, and a large number of them were to do with nutrition. There are about nine questions to do with health, but there are questions about violence against women, questions about nutrition cover, about Anganwadi[20] and functioning of Anganwadi. There are also questions about water supply, handpump in the village. So, 27 points are monitored but not all 27 questions are answered in every monthly meeting of the Village Health and Nutrition (and Sanitation) Committee. However, they look at the checklist and see if any points have come up. Has there been violence against women by men in the village? Has there been Anganwadi without toilets? Such questions come up every month and they try to solve these. In the Swasth Panchayat meeting, Village Health and Nutrition Committee meeting,

13 "BIMARU" is an acronym which stands in for the state names of Bihar, Madhya Pradesh, Rajasthan, and Uttar Pradesh. Ashish Bose, a demographer, coined this acronym in 1980. After examining various demographic indicators, such as Total Fertility Rate and Per Capita Income, among others, Bose concluded that these states significantly lagged behind the India's Southern states. See: www.thehindu.com/data/bimaru-states-the-shoe-still-fits/article7527355.ece

14 The text in the parentheses are clarifications made by Dr. Prabir.

15 The phrase translates to "It's a funeral" in English

16 The Block Development Officer (BDO) oversees all planning and developmental program implementation within their block. See: www.lkouniv.ac.in/site/writereaddata/siteContent/202004221610298999Avinash_Kumar_pub_admin_BDO.pdf

17 The State Health Resource Centres (SHRC; Chhattisgarh in this context) primarily "[provides] technical assistance and capacity building for strengthening of district health systems and act as support to state health systems in strategy development, programme planning, support for innovation and change management." See: www.nhsrcindia.org/practice-areas/kmd/shsrc; and www.shsrc.org/

18 Swasthya Panchayat Yojana is a programme established by the Chhattisgarh Department of Health & Family Welfare, with support from the State Health Resource Centre (SHRC). The programme is intended to strengthen the participation of Panchayats (village-level local and elected self-government bodies) in addressing health-related issues.See: www.shsrc.org/wp-content/uploads/2021/08/Swasth-Panchayat-Introduction-and-Methodology.pdf

19 Panchayat is an institution of local self-government in India which was given constitutional status through the 73rd Constitutional Amendment of 1992 to devolve decentralized power at the local level. See: www.india.gov.in/my-government/constitution-india/amendments/constitution-india-seventy-third-amendment-act-1992

20 The 'Anganwadi' Centres, initiated by the Government of India in 1975, are part of the Integrated Child Development Services (ICDS) Programme, addressing child hunger and malnutrition. Anganwadis are the primary sites of health, nutrition, and early learning program interventions part of ICDS. See: www.womenchild.maharashtra.gov.in/content/innerpage/anganwadi-functions.php. Read about the ICDS program : www.icds-wcd.nic.in/icds.aspx

Mahila Arogya Samiti meeting in the urban area, the same process is followed. They take up issues and say, "Mitanin might solve this, or we will approach the Panchayat leader or member who has attended the monthly meeting, or we will approach the ANM"[21] —the ANM is supposed to be present in the meeting—"or we will ask the Mitanin trainer, depending on whoever is responsible."

I know that sometimes they come back the next month if the toilet has not been open and the Anganwadi children have to use the fields for sanitation purposes, which is not hygienic or safe. Then, there is this next level of meeting called the Sankul [federation], 20-30 different areas where such village-level meetings have taken place, they will have a federation meeting and common problems and issues which are not resolved for over a month are discussed. For instance, the alcohol problem. Say there is an alcohol bhatti [shop] somewhere between two villages, when each village can't solve it individually, both villages get together and say, "Together as women we are going to break the shop, or we are going to threaten the shopkeeper or tell the husbands that they cannot take alcohol." This often gets solved in the federation, if the [incomplete sentence].

Rama Baru: Sorry, you have a minute.

Prabir Chatterjee: Sure, I will not talk more about the monthly meeting or federation meeting or Annual Swasth Sammelan[22], except to say one thing. In Swasth Sammelan we have had very good attendance which are done at the block levels. They happen once a year and BDO is called, and also the MLA and the Political Representative (of the MLA or of the opposition). We have seen more MLAs in Chhattisgarh attend the Swasth Sammelan than any other session on Health in the Assembly itself. So, issues that occur in many villages can be taken up through a street naatak [play] placed in front of MLAs—in front of the elected representative—at the Swasth Sammelan. Urban areas have not had such chances outside of Chhattisgarh. To an extent there is no supervisory structure (in urban areas outside Chhattisgarh), which is important to defend the ASHA and the communitization process. I will leave it there. Thanks.

Rama Baru: We can come back to it later, Prabir. May I invite Dr. Nerges Mistry, please? Thank you.

Nerges Mistry: Good afternoon and thank you for having me on this Witness Seminar on this highly relevant and undebatably [sic] important theme. These Witness Seminars form an exciting approach, but they do approach history through a 2020 vision offered by telescopic hindsight. My testimony, based on a national average viewpoint, acknowledges this belief: that as a spectator who was on the fringes of the communitization process at the time when it started after the NRHM, without deeper participation in the communitization process. I did see communitization before 2005 in action, in FRCH[23] projects in Mandua, Ralegan, and Parinche projects, where we witnessed an evolving sense of community empowerment, both community as a whole and women who took part as the community health workers in these projects. Largely through a form of training, it gave a functional approach which not only included human health but veterinary science, water and sanitation, and microcredit. The lesson from that was [that] these projects took time. It did not happen in a short period of time. I would like to base my talk in this Witness Seminar on the timeliness of these empowerment programs.

So, NRHM was born in 2005, and along with it came the formulation of several structures and processes slightly antithesis to this informal [unclear] of the community-based healthcare system that had been developed by several in the NGO sector.

21 The Auxiliary Nurse Midwife (ANM) cadre of community health workers was constituted in India in the 1950s to focus on basic maternal and child health during childbirth and the pre- and post-partum periods. See: Pyone T, Karvande S, Gopalakrishnan S, Purohit V, Nelson S, Balakrishnan SS, et al. Factors governing the performance of Auxiliary Nurse Midwives in India: A study in Pune district. PLoS One. 2019 Dec 27;14(12):e0226831.

22 The Chhattisgarh government has been organising Swasth Panchayat Sammelan annually since 2005 through the Mitanin programme. These are meetings organised to provide the community with a platform to put forth health service-related grievances, to strengthen community monitoring of government health services, and to make government functionaries of the area aware of the issues that the community is facing, among others. More information available in Hindi here: www.shsrc.org/wp-content/uploads/2021/08/Swasth-Panchayat-Sammelan-Jansamvad-2019-20-2.pdf

23 Foundation for Research in Community Health (FRCH), founded in 1975, is a not-for-profit organization conducting conceptual and field studies to better understand factors that impact health and health services, primarily in rural India. See: www.frchindia.org/about.html; list of FRCH projects: www.frchindia.org/project.html

This was the basis of the NRHM. There was a task force[24], ASHA mentoring group[25] and Advisory Group of Community Action[26]; the formation of committees—Village Health and Sanitation Committee, the RKS[27]s and so on. What followed was the inevitability of scaling up to national measurements almost in an industrial manner, which Dr. Prabir alluded to in his talk. The ASHA and the Village Health and Sanitation Committee and the upward Block- and district-level structures were seen as a phase of communitization. Indeed, they involved communities in decision making and participation. ASHA, during the beginning of NRHM, that is the period I am referring to, was armed with eulogies and accolades, and was projected to lead the community processes from the grassroots. I am neither going to dwell upon individual victories of the communities, which undoubtedly were there, nor look at the procedural glitches and challenges the communitization process faced, like irrelevant expenditure, timely receipt of funds and so on. The singular point I wish to make is regarding the timing of the communitization process. I will start with ASHA as she developed in the early phases of the NRHM.

Under the NRHM, it has taken an extended period of time for the ASHA to evolve as a somewhat empowered worker; perhaps a little more than the community health workers who were raised through pioneer NGOs in a slightly different spirit. For example, Rakhal would remember [from the meeting at MoHFW]: the modules on the ASHA training on primary illness care were all printed and ready even before they were reviewed by the mentoring group. The softer ones, like communication and empowerment, came much later, thanks to the efforts of the ASHA mentoring group members. Particularly, I remember the efforts of CHETNA[28]. It was at this initial phase the confidence was low in the ASHA. With shaky support from the community, ASHA was thrust into the communitization process with little respect from the village committees. I am only talking about the nascent phases of NRHM where the ASHA faced—[was] characterised by doubt and scepticism at best by her neighbourhood. Therefore, in the initial phases, ASHA failed to meet her functional level in tandem with the communitization process and aspiration. This for her remained unsubstantially [sic] realised. The very process of communitization in communities through committee formation relied extensively on an idealistic idea of voluntarism and idealism and dismissal of contextual factors like social division in societies and patriarchy. Not to mention the lack of information or perception of the niche that was expected to be filled.

So, I put forward my view that a communitization process of VHSNCs[12] and upwards should have been staggered until the ASHA had a shot at undertaking broader local community functions other than basic human illness care, like school health [and] domestic violence. In the process side, there was more orientation, information, and handholding in playing their roles at the ASHA level and at the level of these committees. The committees, like ASHA, should have gravitated to a broader—gradual broadening of their functional ambit, moving beyond [the] requirements of illness

24 Under NRHM, task forces were set up constituting experts, institutional representatives, and NGOs. There were eight task groups which were assigned tasks with respect to 1) goals of the NRHM, 2) strengthening public health infrastructure, 3) role of Panchayati Raj Institutions (LSGs), 4) the ASHA programme (see note 10), 5) technical support to NRHM, 6) health financing, 7) district planning, and 8) public private partnerships. See: www.nhm.gov.in/WriteReadData/l892s/nrhm-framework-latest.pdf; www.nhm.gov.in/images/pdf/communitization/task-group-reports/tasks-for-task-groups.pdf

25 The National ASHA Mentoring Group, constituted in 2005 by the Ministry of Health and Family Welfare (MoHFW), consists of health professionals and experts who provide technical and advisory support to states and the Centre for the implementation, monitoring, and mentoring of the ASHA programme. See: www.nhm.gov.in/index1.php?lang=1&level=2&sublinkid=178&lid=251

26 The Advisory Group on Community Action, consisting of prominent public health professionals, was set up by the MoHFW in 2005 to advise and guide the community monitoring and planning initiatives under the NRHM (see note 9). See: www.nrhmcommunityaction.org/agca/

27 Rogi Kalyan Samitis (RKSs) or Hospital Management Committees, introduced as part of the NRHM, serve as platforms "to improve the functioning and service provision in public health facilities, increase [community] participation and enhance accountability." From Government of India. (n.d.). Guidelines for Rogi Kalyan Samitis in Public Health Facilities. Ministry of Health and Family Welfare. Retrieved from www.nhm.gov.in/New_Updates_2018/communization/RKS/Guidelines_for_Rogi_Kalyan_Samities_in_Public_Health_Facilities.pdf

28 CHETNA is an NGO that addresses the health, nutrition, and other developmental needs of children, adolescents, and women. See: www.chetnaindia.org/what-we-do/#thrust-areas

care, particularly in committees close to the village level, to issues like social production schemes and specifically those which impinge on nutrition and health, like soil and water configuration issues, etc. A specific example I would like to cite is the National Family Benefit Schemes[29] introduced in 1995 under the Social Assistance Program[30]. For instance, programs which deal with the death of a breadwinner and more serious contingencies that poor households have to face. Such schemes are languishing and need to be revised and reinforced with urgency and emphasis that can be best done at the community level itself. The takeaway message for communitization, or community participation included as better Health for All in the Alma-Ata Declaration[31], is that it can be embedded but just can't be straight jacketed or institutionalised in a single homogenous effort. It needs to grow incrementally and contextually on the [border] line of guiding principles, and with robust experimentation.

I recall [that] Dr. Rajnikant Arole[32] voiced his opposition to the way that the NRHM had rolled out in its initial phase. On a winter morning at the meeting on community health at Tata Management Centre[33] in Pune, he said, "Why do you want this? Let hundred flowers bloom." That was his precise statement—I remember that very well. Coming to the contemporary situation, I will end with that, while talking about one of the questions that was specifically asked about: "What are the lessons for the Swasth Bharat Yojanas[34] [PM Atmanirbhar Swasth Bharat Scheme] that are being rolled out?" Largely, you will have to realise that these new Yojanas that are coming out fill gaps existing in public health infrastructure especially in primary and critical care facilities. They also look at IT-based surveillance systems, diagnostic labs, lab-based surveillance systems, and empowering primary [care] workforce to deal with emergencies that may arise. There is no debating about the importance of this. However, communitization has sort of been replaced in these schemes by a new buzzword: "public-private engagements". "Public-private engagements" is the new buzzword with the word 'communitization' jargon seeming to be slightly in the background, if not completely out. So, [regarding] communitization, no debating [that it] is essential for any Yojanas and schemes, even if one is infrastructural and technical leading one. However, what communitization could impinge on in this changed scenario is the provision of public information of these schemes and regulation norms for redressal of grievances. This is a part of communitization that should be brought into these types of Yojanas, also to generate accountability in health system components that form these Yojanas. The preparation of a community and primary [care] workforce to deal with this is to not use a narrow vertical approach but what environmentalists call [a] 'one health approach'. Practically, this translates to a broader sensitisation of the community of the workforce at the grassroots and taking into account not only the traditional persistent health threats, but newer emerging ones which cannot be limited today to only in the local context. We can refer to climate change, antimicrobial resistance or the pandemic we are living through. So, the challenge is in retaining compassionate, equitable, person-centred care in complex health challenges, and

29 The National Family Benefit Scheme "aims to provide a lump sum family benefit of Rs. 10000/- to the bereaved household in case of the death of the primary breadwinner irrespective of the cause of death." For more information, see: www.transformingindia.mygov.in/scheme/national-family-benefit-scheme/#intro

30 The National Social Assistance Programme (NSAP), administered by the Indian Ministry of Rural Development, is a group of schemes intended to raise the standard of living, improve population health, provide free education to children, and provide people with an adequate means of livelihood, among others. The schemes include the National Old Age Pension Scheme, National Family Benefit Scheme, and National Maternity Benefit Scheme. For more: www.nsap.nic.in/circular.do?method=aboutus

31 The International Conference on Primary Health Care, held in Alma-Ata in 1978, was the emergence site of the Alma-Ata Declaration. The Declaration was a milestone in global advocacy, highlighting primary health as essential to achieving health for all. See: www.who.int/teams/social-determinants-of-health/declaration-of-alma-ata

32 Rajanikant Arole was the co-founder of the Comprehensive Rural Health Project at Jamkhed, India. See: Pincock S. Rajanikant Arole. The Lancet. 2011 Jul 2;378(9785):24.

33 Read more about the Tata Management Centre: www.tmtctata.com/about-tmtc/

34 The PM Atmanirbhar Swasth Bharat Yojana programme, announced in 2021, aims to strengthen health systems across the continuum of care (i.e., at primary, secondary and tertiary levels), and prepare it for effective response to pandemics and other health emergencies. See: www.pib.gov.in/PressReleasePage.aspx?PRID=1704822

maintaining the trust of the community in health delivery institutions that can only ignore this aspect of trust. I will stop here. Thank you.

Rama Baru: Thank you so much, Nerges. Now, may I request Thelma to please come in?

Thelma Narayan: Thank you Rama, and thanks to the organisers for organising this Witness Seminar. I agree with Nerges that Witness Seminars offer huge potential, but it has its limitations. To go through deep history in 10 minutes is a tough call. I will dive straight in. My experience comes from actually starting with community-based work based in St. John's Medical College[35], which was an academic institution. It was a medical college which had a specific mandate to promote community health, and we were from the Department of Community Health[36]. We had a huge library and a dynamic team; we worked in seven different rural areas. It was the intellectual discussions that took place there that influenced me greatly. The historical origins of community participation actually start with the Bandoeng conference[37] in 1937, which then led to the formation of the Sokhey Committee[38] in 1938, which started its work in 1939. This Sokhey committee actually spoke of having one community health worker per 1000 population in India, assuming that we were going to get Independence shortly, and that this goal should have been reached in five years. They talk of basic training of 9 months; we are talking about 1940 when the interim report was submitted. This influenced the Bhore Committee[39], which also took forward the concept of community participation along with a lot of other things in the development of the health system, structure of the health system, etc.

If you read through the early documents right from the Five-Year Plans and Committee Reports, such as [of] the Mudaliar Committee[40], Shrivastava Committee[41] and several others, one will see that, that [they] were reflective about the situation and level of progress, and recommendations from each of them were a step forward. Well, the community development program[42] started in 1952, it was the year when Primary Health Centres[43] were launched in India. In 1929, Karnataka had the first

35 St. John's Medical College Hospital was established in Bengaluru, India in 1963. At the time of its establishment, the core aim was to "[train] healthcare personnel committed to serving the poor in the margins". See: www.stjohns.in/about_us.php

36 "We worked in seven rural centres, in Bangalore urban, as well as in tea plantations with the UPASI (United Planters Association of South India). We set up village health committees in these sites and identified and trained community health workers. The institution had initiated this approach in the late 1960s early 1970s. In the tea plantations, community health workers were called Link Workers. This is documented. The Mallur Health Coorperative in Karnataka has been written about in two ICMR Monographs. We also upgraded Health Sub Centres by posting two medical interns who lived there and were supported by a community health worker and a local health committee. Our team engaged with sociologists, development workers, social analysts and activists. So, when we engaged with the JSA and the NRHM we brought this 25-to-30-year experience with us. Each of us engaged with the NRHM from our own respective inspirations, experiences, world view and positionality." (Annotation provided by Dr. Thelma)

37 The Bandeong (Bandung) Conference on 'rural hygiene' was held in 1937 in Bandung, Indonesia. "The Conference approached the problems of rural hygiene from an intersectoral and interagency perspective and focused not only on the need to improve access to modern medicine and public health but also on the fundamental challenges of educational uplift, economic development, and social advancement." See: Brown TM, Fee E. The Bandoeng Conference of 1937: A Milestone in Health and Development. Am J Public Health. 2008 Jan;98(1):42–3.

38 The National Health (Sokhey) Sub-Committee of 1938 recommended for a Community Health Worker (CHW) for every 1000 village population, the CHW being trained in 'community and personal hygiene' and first aid. The committee also emphasized the social aspects and determinants of medical and public health. See: Banerji D. Politics of Rural Health in India. International Journal of Health Services. 2005;35(4):783–96.; Read the report: www.ruralindiaonline.org/en/library/resource/national-planning-committee-series-report-of-the-sub-committee-national-health/

39 The Bhore Committee, also known as the Health Survey & Development Committee, was established in 1943 and chaired by Joseph Bhore. It made recommendations for the restructuring of health services in India, foregrounding a curative and preventive approach to medicine. See: www.nhp.gov.in/bhore-committee-1946_pg

40 The "Health Survey and Planning Committee", headed by Dr. A.L. Mudaliar, was established in 1962 to evaluate the health sector's performance since the launch of the Bhore committee report of 1946. View report: www.nihfw.org/Doc/Reports/Mudalier%20%20Vol.pdf

41 The Srivastava committee was set up in 1974 as "group on Medical Education and Support manpower" to recommend actions on reorienting medical education as per 'national priorities', and develop a curriculum for health assistants to function as link between medical officers and Multipurpose Workers (MPWs) See report: www.nhp.gov.in/sites/default/files/pdf/Srivastava_Committee_Report.pdf

42 The community development program was initiated in 1952 to develop rural areas and "initiate a process of transformation of the social and economic life of the villages." See: www.niti.gov.in/planningcommission.gov.in/docs/plans/planrel/fiveyr/1st/1planch15.html.

43 A Primary Health Centre (PHC) is the 'first port of call' for people in rural areas to consult a government designated doctor for ailments

Primary Health Unit in Ramanagaram. The person who worked in Ramanagaram was our Professor of Community Health at St. Johns. So, we were part of this in a deep manner. I would also like to believe that this thrust given to community participation and the urgency for all to access health care came from the freedom struggle. If you see the Sokhey Committee report, it was a part of the freedom struggle. It was an Asian approach towards achieving access to the entire community and to build on local health traditions. The wording of the Sokhey Committee is actually very good. It is extremely detailed. And so is the Bhore Committee.

I give this background to understand this more and would be happy to have one-on-one conversation as you [TGI] suggested. Because in the Raj Narain[44] time, we had the first Community Health Volunteers Scheme[45], and the initial thought of changing the dynamics using local health traditions, AYUSH[46] and all that—these came in 1977 (there are printed CHV Manuals by GOI which we used in St. Johns too[47]), which predated Alma-Ata[31]. Alma-Ata was greatly influenced by Indian contribution and presence. It has a global influence, as was mentioned by Dr. Halfdan Mahler, DG WHO, when the Alma Ata Conference was held. He had worked in Bangalore National TB Institute (NTI), whom we knew personally very well, [Mahler] says that the concept of primary healthcare arose from his work in India, in the National TB institute. The seminal work of Stig Anderson and Dr. Banerjee[48,49] says people need not be educated regarding what to do when they develop TB, but the health system needs to have the capacity to diagnose them [people] early and initiate treatment. People driven by their symptoms seek care and support, but they are often not adequately diagnosed and treated. The NRHM, when it came—and there's the history—, the Jan Swasthya Abhiyan[50] took this up in a big way... the People's Health Movement[51]. This started around 1998-1999, with the first People's Health Assembly in India, the Jan Swasthya Sabha[52] and the Global People Health Assembly[53] in Bangladesh in December 2000. They were challenging WHO. WHO refused to participate in the Assembly, though they were invited. Four people who came had to

---

and if they are referred to the PHC from a sub health centre (first point of contact between the primary health care system and the community. See www.nhm.gov.in/images/pdf/guidelines/iphs/iphs-revised-guidlines-2012/sub-centers.pdf . A PHC in India covers a population of 20,000 in difficult/hilly regions and areas with tribal population, and a population of 30,000 in plain areas. The government's guidelines for PHCs in India can be accessed here.

44 Raj Narain was the Union Minister of Health & Family Welfare during the first Janata Party government at the centre (1977-78). (Annotation provided by Dr. Thelma)

45 The Village Health Guides (VHG) is a national community health worker program launched in 1977 by the Government of India "to provide preventive, promotive, and basic curative care to rural populations." It was renamed to "the Community Health Volunteer (CHV) scheme" in 1979, and in 1981 "Village Health Guide Scheme." See: Strodel RJ, Perry HB. The National Village Health Guide Scheme in India: lessons four decades later for community health worker programs today and tomorrow. Human Resources for Health. 2019 Oct 28;17(1):76.

46 "AYUSH" is the acronym which stands for the names of medical systems practiced in India, namely "Ayurveda, Yoga & Naturopathy, Unani, Siddha and Homeopathy." See https://www.nhp.gov.in/ayush_ms

47 Dr. R.M. Verma a neurosurgeon, who was the founder Director of NIMHANS, Bangalore, who we knew was DDG Health Services, Government of India, in 1977 and supported the rural health work including the CHV scheme (annotation provided by Dr. Thelma)

48 Dr. Thelma has referred to the work by Dr. Stig Anderson and Dr. Banerjee in her PhD thesis: Andersen S, Banerji D. A sociological inquiry into an urban tuberculosis control programme in India. Bull World Health Organ. 1963;29(5):685–700.

49 "My PhD thesis on health policy process and implementation at the London School of Hygiene and Tropical Medicine in the mid-1990s, which is available online, had one hypothesis of the lack of a countervailing power from the voluntary or alternative health sector in India." (Annotation provided by Dr, Thelma) Link to Dr. Thelma's PhD thesis: https://researchonline.lshtm.ac.uk/id/eprint/682263/; Dr. Thelma has shared another article summarising some of the sociological research on TB in India which helped shape the National TB Program (NTP) https://ntiindia.kar.nic.in/cddistrictlevel/ielearn/category/Scientific%20Articles/STSOJA99.pdf

50 Jan Swasthya Abhiyan (JSA), amalgamated in 2001, is the India chapter of the People's Health Movement. See: http://phmindia.org/about-us/

51 The People's Health Movement (PHM) serves as an overarching campaign for activism on priorities related to the social determinants of health with its central focus being the Health for All agenda. See https://phmovement.org/health-for-all-campaign/

52 The first National Health Assembly (Jan Swasthya Sabha) was held in 2000 in Kolkata, West Bengal. It welcomed 18 national networks, represented by over 2000 delegates from 19 states. At the Assembly, the 'People's Health Charter' was adopted; it asserted 20 key demands towards achieving 'Health for All'. See RAJAGOPAL M. A People's Charter for Health and beyond. The National medical journal of India. 2001;14(2):67. http://archive.nmji.in/archives/Volume-14/issue-2/editorials-2.pdf.

53 Several international organisations, civil society, women's groups, and NGOs collectively mobilised themselves towards reviving, reclaiming, and achieving the Health for All agenda. This led to the first People's Health Assembly held in 2000 in Bangladesh. A People's Health Charter was drawn by the participants during the event. the See https://phmovement.org/the-peoples-charter-for-health/

take leave and three of them had to leave their organisation, as it was considered bad politics to join civil society groups. However, I think the Health Assembly made a big change in [inaudible]—Dr. Sundar and all of us were deeply involved in the organisation as many of us are here—and that gave [it a] sort of a momentum. The Medico Friend Circle[54] [MFC] had already created the think tank and had supported the process intellectually, based on the experience of all of them who were members of MFC. Though there was a lot of experience in the country through NGOs, there was no countervailing power to change the larger health system trajectory. The formation of JSA[50] helped in the formation of a countervailing power to a large extent.

It [JSA] brought together all these 21 networks, women's movements—six groups, environmental movements, and others. It played an important role in engaging with the political process and policy process. In JSA—Sundar knows—, some of us were wanting to engage with the government because we felt [that] unless there was an active process of constructive engagement, larger health system and health policy change would not occur. There are two or three means: one could engage, one could confront, or one could probably do both. So, the INA strategy—Indian National Army[55], my father was a member there—is to work both explicitly and implicitly with the policy process as well as with community formations, social movements, etc. It was that combination that helped the first draft National Health Policy[56] being critiqued by JSA and then the dialogues with political parties prior to the 2004 General Elections. When the thing about having one health worker again came, we actually replanted the 'one health worker' concept from the 1930s again into the policy dialogue, but it was not being acted upon. So, this brought together a vibrant group of people; we were enthusiastic, optimistic, and hopeful. Now, the subsequent experience has been that there have been different voices and experiences. In a large country like India, where the sociocultural situations are different and histories of the states are varied, you can't have one technical implant that will equally work for the whole country, unless it is locally grounded in the socio-political process of that particular state—some states have grown, some haven't—, but I think the advantage of this process is that it definitely created some degree of acknowledgement that work can be done.

So, in communitization, besides JSA, there were other key organisations including the FRCH, SATHI-CEHAT[57]. All components and constituents of JSA played a role. I think the MFC bulletin carried many useful articles in this regard, meetings were held and there were healthy debates—Myth of the Mitanins[58] [is] an article written by Binayak Sen in the MFC Bulletin that had generated a discussion which was actually necessary. I think such democratic debate is something that is always needed for the communitization process. Communitization, as we all know, has six to seven components: VHSNCs[12], ASHAs[10], the Charter[59], Patient Welfare Society[60],

54 Medico Friend Circle (MCF), constituted in 1974, is a national organization of "secular, pluralist, and pro-people, pro-poor health practitioners, scientists and social activists interested in the health problems of the people of India." They work to contribute to various debates on health-related issues, such as primary health care, occupational health, women's health, universal health care, etc. See: https://samawomenshealth.in/medico-friend-circle/

55 The Indian National Army (INA) was an Indian military group which sided with the Japanese and fought against the British from 1942 to 1945. "The INA was the result of an informal alliance between the radical expatriate political leaders of the Indian Independence League (IIL) and the Imperial Japanese Army (IJA)". See: the 'abstract' of the book chapter Havers R. Jai Hind!: The Indian National Army, 1942–45. In: Bennett M, Latawski P, editors. Exile Armies [Internet]. London: Palgrave Macmillan UK; 2005 [cited 2022 Jun 13]. p. 55–67. Available from: https://doi.org/10.1057/9780230522459_6

56 The first National Health Policy was launched in 1983, underscoring the "need for providing primary health care with special emphasis on the preventive, promotive and rehabilitative aspects." See: https://www.nhp.gov.in/sites/default/files/pdf/nhp_1983.pdf

57 SATHI (Support for Advocacy and Training to Health Initiatives), launched in October 1998, was originally a part of CEHAT. On 1 April 2005, SATHI was transformed into an 'action research centre' of the Anusandhan Trust. Its headquarter is located in Pune, Maharashtra, India. It works on addressing health rights-related issues through civil society organization (CSO) partnerships. SATHI also facilitates local-, district-, state-, and national-level advocacy. See: https://sathicehat.org

58 The reference of the article is: Sen B. Myth of the Mitanin: political constraints on structural reforms in healthcare in Chhattisgarh. Medico Friend Circle Bulletin. 2005; 311:12-7. (Link to the article online is unavailable)

59 The National Rural Health Mission (NRHM) mandates a 'Citizen's Charter' at the Community Health Center/Primary Health Center level. The Charter provides people with information on what services are available to them, the quality of services to which they are entitled, and the grievance redressal mechanism. See: https://www.jknhm.com/citizencharter.php

60 Same as Rogi Kalyan Samiti (see note 26).

untied funds for VHSNCs and local health facilities, etc. Now, together, there has been an effort to have a groundswell, and despite us not reaching all the aspirations, I would not dismiss this. I think this is a hugely courageous and an important experience globally. I don’t think this kind of experience is found in a country as large as ours with a huge diversity. There is learning in this. The WHO’s World Health Report[61] of 2008 mentioned this, then the Rio Political Declaration[62] also had a workshop on institutionalisation of community processes for health. So, it has had ripple effects elsewhere. In terms of success and failures, I think accountability was a very important component, because it was new in the dialogue in that period of time. Social audits[63] were happening with MGNREGA[64] etc, but the CBMP (community-based monitoring and planning, later called Community Action for Health [CAH]) established accountability systems in the public health systems [audio lost].

Rama Baru: We can’t hear you, Thelma. You may switch off your video, we can’t hear you. I think she is frozen.

Rama Baru: Hi Thelma, we lost you briefly.

Thelma Narayan: [Continuing] Accountability is a significant point in communitization. Coming to section C [impact and evaluation], I think there have been some successes in the sense that there is a huge creation of human resources who are thinking differently. The concept of community engagement, community action, [and] accountability has been internalised in the system, but it is met with a lot of opposition and therefore there has been a delay in funding of proposals. In CAH, we have covered only about 50% of India’s population, though it has been so many years—2007, 2008, 2009 and so on. I think if every citizen has the right to participate, we cannot accept that [shortfall in coverage] as being okay. This process has not been measured or documented adequately, therefore I like this Witness Seminar and research. Another failure I would say is that the gender issues have not been adequately dealt with either. Women have been sort of treated in a patriarchal manner, whether it is ASHA, ANMs, or any other. I would like to flag that gender has been a missing area. Though it has been brought up—some had meetings and workshops where it has been discussed—, but it still hasn’t changed the ground reality. NUHM[65], again, has been limited in its reach. It is necessary. It is extremely important, and I think the power sharing has been stymied.

While there are Health and Wellness Centres[66] and usage of words like Community Health Officers[67] and Primary Health Care offer a lot of opportunity, I think civil society needs to be involved. I don’t think it is as closely involved as it was in the past. I think we need to measure this more carefully. I will close now Rama.

Rama Baru: Thank you so much Thelma. Now, I invite Rakhal Gaitonde.

Rakhal Gaitonde: Thanks. This is very interesting. It

61 View the WHO World Health Report 2008 here: https://www.who.int/whr/2008/whr08_en.pdf

62 The Rio Political Declaration on Social Determinants of Health was adopted during the World Conference on Social Determinants of Health in 2011, and it stands for “global political commitment for the implementation of a social determinants of health approach to reduce health inequities.” View the report here: https://www.who.int/sdhconference/declaration/Rio_political_declaration.pdf

63 The social audit involves assessing official records to verify whether the expenditures reported by the state are equivalent to the true amount spent. See https://nrega.nic.in/netnrega/SocialAuditFindings/sa_home.aspx.

64 The Mahatma Gandhi National Rural Employment Guarantee Act (MGNREGA), launched in 2005, is a program of the Government of India which “[provides] at least 100 days of guaranteed wage employment in a financial year to every rural household whose adult members volunteer to do unskilled manual work”. See https://nrega.nic.in/Circular_Archive/archive/nrega_doc_FAQs.pdf

65 The National Urban Health Mission (NUHM) is a ‘sub-mission’ of the National Health Mission (NHM) aimed at addressing the health care needs of the urban population, especially the urban poor. To achieve this, the NHM works to improve the availability and quality of essential primary health care services, and to reduce the need for out-of-pocket treatment expenditure. The NHM was launched by the Government of India in 2013 subsuming the NRHM and the NUHM. See http://nhm.gov.in/index1.php?lang=1&level=1&sublinkid=970&lid=137.

66 Ayushman Bharat, which aims to make health care services more comprehensive, has two components: Health & Wellness Centres (HWCs) and Pradhan Mantri Jan Arogya Yojana (PM-JAY). HWCs “deliver comprehensive primary health care, that is universal and free to users.” See http://ab-hwc.nhp.gov.in/

67 Community Health Officers (CHO) are mid-level health providers in India designated with the primary health team at the level of a sub-health centre/Health and Wellness Centre as part of the Ayushman Bharat (AB) programme. See about CHOs in the ‘operational guidelines’ of the AB programme. See: https://www.nhm.gov.in/New_Updates_2018/NHM_Components/Health_System_Stregthening/Comprehensive_primary_health_care/letter/Operational_Guidelines_For_CPHC.pdf

is like going down in the memory lane. What I want to do is present a few memories as a witness, not necessarily linking them in the form of narrative.

So, one of the first memories of the NRHM process is about a meeting we have with Gopalakrishnan in Delhi. Dr. Antia led the group as it were, and a large group of people had come together, as Thelma had described, essentially to emphasise to the government that the proposed community health worker, the ASHA at that time, was inadequately designed, and it needed to be much more a worker with role and responsibility coming from experience of various civil society groups, NGOs, [and] CBOs in India historically. That meeting, to me at least, created the energy, a sort of confidence. We took it forward as a movement—as civil society—when we began to design communitization, so on and so forth. This actually led me to explore subsequently about where this comes from. Interestingly, as for ASHA, as Thelma sort of traced a longer history, the ASHA was already there and proposed by the Sushma Swaraj[68] government. So, there was already a template on file which got pushed when the UPA government[69] came; and it was a person with a bunch of contraceptives, that was the original plan. I remember seeing a presentation on that, and everyone going ballistic about that. However, that continuity is what I want to highlight. Digging up a bit, you find that, when communitization came, I could point out at least five strands. One was the continuity between NHRC[70]'s public hearings and the whole process of NHRC's hearings, and then moving to NRHM phase. Therefore, accountability got a big boost. Interestingly, a number of officers—Jalaja Madam, who was in NHRC, became NRHM head.

So, for civil society members, it was a relief to see a familiar face in Nirman Bhavan[71]. Similarly, for example, Amarjeet Sinha and Tarun Seem had experience in working with civil society organisations prior to their role. It is interesting how all three of them came together, creating space for JSA's entry. Of course, there was a larger political space because of the elections and what it meant and how it was interpreted, but I am just pointing out some very specific points. Actually, RCH-II[72], as Mr. Nanda always used to talk about the fact how RCH-II was trying to gear up into what community needs assessment actually meant. In a way, probably communitization's time was right, there were [the] right people, right energy, post-election scenario, ideas, and even the government wanting to do something. So, that is a point that I wanted to make at the national level. A few points, and I will spend more time on Tamil Nadu.

The first conversation I remember was with Dr. Padmanabhan, who was the Director of Public Health back then, sitting in a makeshift office. First time I went to his office, I introduced myself and the whole process. The first thing he said was, "Oh, in Tamil Nadu, we don't need community monitoring, because we have a very active media. Anything goes wrong, the media highlights it. That is one of the biggest accountability mechanisms we have." Therefore, he literally dismissed the community monitoring process and that was the end of the meeting.

I did manage to get a chance to get into his office again because of the NHRC [National Human Rights Commission] public hearings 70. I am repeating this because it is an important sort of door opener, as it were. Because NHRC, after public hearings, had put in a once-in-two-years review process for their action plan. Every state was supposed to

68 Sushma Swaraj was the Union Cabinet Minister for Health and Family Welfare during 2003-04 when the coalition regime of the National Democratic Alliance was running at the Centre and led by the Bharatiya Janata Party (BJP). See here: http://loksabhaph.nic.in/Members/MemberBioprofile.aspx?mpsno=3812&lastls=16

69 The United Progressive Alliance (UPA) is a coalition of political parties in India formed after the 2004 general election. The largest party in the UPA alliance is the Indian National Congress. See: https://journals.sagepub.com/doi/10.1177/0974928416654367?icid=int.sj-abstract.similar-articles.1

70 The National Human Rights Commission (NHRC) is a public and autonomous body with the mandate of "promotion and protection of human rights" in the country. See https://nhrc.nic.in/about-us/about-the-Organisation

71 Nirman Bhavan situates the Ministry of Health and Family Welfare, Government of India.

72 The Reproductive and Child Health (RCH) Programme was launched throughout India on 15 October 1997. The first phase of RCH worked toward "achieving a status in which women will be able to regulate their fertility, women will be able to go through their pregnancy and child birth safely, the outcome of pregnancies will be successful and will lead to survival and well being of the mother and the child." RCH-II aimed to reduce India's total fertility rate, infant mortality rate, and maternal mortality rate. See: https://www.nhp.gov.in/reproductive-maternal-newborn-child-and-adolescent-health_pg and https://nhm.gov.in/index1.php?lang=1&level=1&sublinkid=794&lid=168

report to NHRC on what steps were taken for the action plan[73] on Right to Health. The process was that the state would make a presentation, and the JSA representative from the state or [a] civil society person would make a response. Padmanabhan Sir got his NHRC notice exactly a few days after the meeting was called, so he saw myself, Ameer [Khan], and other members from the JSA. So, he called us back for a meeting and that's when we ironed out the whole process of him accepting and starting a pilot project. Just to show the NHRC public hearing, and you know how it was tied up with the implementation of the subsequent process of CAH.

The third point in Tamil Nadu is another interesting statement given by the Director of Public Health who said community monitoring is a bad idea. In his words he gave a metaphor: "Think about when you invite a guest to your house and your house is in disarray. Will you call your guest to your house which is in disarray? You won't, right? You will first put it in order and then call a guest." Basically, what he was trying to say is that we know our house is not in order, so why would I call people to come and find fault, when I myself know that my house is not in order? So in a way, there was this sort of end of the initial 10 years of liberalisation phase and a bit after it which saw a freeze in spending, i.e., 1991-2004.

Post-1991, there was this huge stress in the system because of [a] lack of finance [and] many positions were vacant. Even in Tamil Nadu, we weren't able to expand the way we wanted, especially in primary healthcare. There was this sense of struggling between not being able to do what you want and having people literally breathing down your neck. I will just leave it there.

Then, I moved to the community where I had a 'light bulb' moment where this young girl from Vellore[74] district, in one of the meetings we were attending—this village planning meeting—, in the end of the meeting, she said "Sir, enakku oru doubt" ("I have a doubt"). We said, "Yes, what is the doubt?" She said, "Listen, you are doing all this training for PHCs and making the PHCs functional, but my real problem is with the ration shop. Can we do something about the ration shop?" To me, that was a moment where we felt [that], okay, this process is taking root.

I remember another conversation where this big argument on whether the Village Health and Sanitation Committee could actually spend their Rs. 10,000 untied funds on streetlights. Of course, it was immediately cut down by people saying it is a streetlight, you can't do it, it is not health, 'til one of the members argued that the streetlight would be outside the public toilet, and because of the streetlight, [the] public toilet would be usable by women in the dark. He argued that it is a public health problem, and he won the argument, and the streetlight was put on. These two examples for me, in a way, epitomised, among many wonderful stories, the way in which giving people community spaces to discuss and open up would really create its own energy.

Lastly, I want to make one remembrance or witness [narrative] or whatever. I remember this attempt, which was years long, trying to get the ASHA Mentoring Group[25] to get a common meeting with the AGCA[26]. In fact, it never happened. It probably happened once; I don't remember. I think that is very important. At one level, you start off the ASHA, and as Nerges and others said, she was put out literally there with no support. Then you hold on to the support systems in terms of the village committees—I am here referring to the mismatch between the implementation of the ASHA program and the implementation of the Village Health and Sanitation Committees which was supposed to support the ASHAs. However, the two large systems just don't talk. To me, that is a key point we [can] learn—or at least I [can] learn—and take away from this whole process. There are a few other points, but I will just stop here.

Rama Baru: Thank you, Rakhal. May I now invite Mr. Sundararaman? I think it's very interesting to see the juxtaposition of different points of view on this entire process.

73 The recommendations of the National Action Plan to Operationalise the Right to Health Care emerged from the public hearings organised by the NHRC and JSA in 2004: https://nhrc.nic.in/press-release/recommendations-national-action-plan-operationalize-right-health-care

74 Vellore is a district in the north of Tamil Nadu, a state in India.

T. Sundararaman: I don't know where to start, but this story of how ASHA began or how the Mitanin began and what are the factors that came into play—pretty much like a Rashomon[75] type description. There are so many truths. If one was there at that time, and I'm saying even in 2006, Mitanin, there would be five different stories of its origin. In ASHA, even later, there are even more stories, and I don't think that any of them are not true, but I don't think that we will be able to ever sort that out. So, I think that sometimes I look at it as a constellation of things that came together. As one senior officer put it, "The stars are in the right places", so to speak. A number of different things came together at a given point of time. One of these events, is, I think, the National Health Assembly[52], which happened in 2000, organized by a large number of civil society organizations which later came to be called the Jan Swasthya Abhiyan[50]. This National Health Assembly adopted a follow-up work plan with a number of elements—the last one was a health worker in every village, along with an emphasis on community processes, and decentralization was very important.

Then, in 2002, spinning off from that and related to that were the Right to Food[76], the Right to Education[77] and the Right to Information[78] campaigns by a number of rights-based movements, and then the collapse of "India Shining"[79] in the 2004 General Elections. The latter was read within the Congress as a return to a greater role of government in provisioning of health care and social welfare, and this was important for the party's pro-people image. So, the first attempt was by the bureaucracy in the Health Ministry to sort of read the new politics into the older existing design of RCH-II[72]. This was sort of resisted by a group of seven or eight health activists led by N.H. Antia and AR Nanda, who fixed up an appointment to meet the Prime Minister, Dr. Manmohan Singh[80]. I was one in the group. We didn't get to meet Manmohan Singh, although Antia visited him later separately. We had a discussion with the Joint Secretary in the PMO, Mr. Gopalakrishnan—quite a fascinating person—, and he himself was a votary of many of these views. He had already done a number of Missions in Madhya Pradesh. He had good confidence in one officer, Mr. Amarjeet Sinha, and therefore their individual personalities and their views played an important role. Plus, a very strong commitment in these officers, and in the political mood and in what our delegation was pushing for, which was that we do not go in the health sector reform route viz. a market-based reform was out. I think that was the important part, that against the background of the existing policies, this was a sort of conscious political choice. It was met with internal criticism that, if you spend on the public health system, it will only be a waste, to which the reply that was offered was: "Yes, of course, it cannot be an ineffective public health system, but we will not do 'health sector reform', we will do 'architectural correction'[81] of it", and this term was invented before the content. Then,

75 The "Rashomon effect", also known as "Kurosawa effect", is a phenomenon involving the same event being interpreted by different people in different ways. See: https://www.thehindu.com/opinion/op-ed/rashomon-effect/article25194224.ece

76 In 2001, the CSO People's Union for Civil Liberties (PUCL) submitted a writ petition, demanding: "(1) a national Employment Guarantee Act, (2) universal mid-day meals in primary schools, (3) universalization of the Integrated Child Development Services (ICDS) for children under the age of six, (4) effective implementation of all nutrition-related schemes, (5) revival and universalization of the public distribution system", and more. This petition catalyzed the inception of the Right to Food campaign in 2001. See more http://www.righttofoodcampaign.in/about

77 The enactment of the Right to Education Act of 2009 in India, guaranteeing "free and compulsory" education for children in aged six to 14, was a result of a countrywide people's mobilisation and public deliberations demanding universal opportunities for children for education. See more: https://dsel.education.gov.in/rte

78 The Right to Information (RTI) Act, implemented in 2005, requires that all government institutions meet public demands for information. The National Campaign for People's Right to Information (NCPRI) launched in 1996 was key in propelling the legislative change. The network consisted of people's movements and local groups that were campaigning regarding the development issues in urban and rural regions. See Baviskar A. Winning the right to information campaign in India. IDS Research Summary, 08. 2008.

79 "India Shining" was a campaign launched by the NDA government in 2004. The campaign sought to celebrate the neoliberal reforms in India as success and broadcast India as an emerging market in the global economy. See here: Kaur R. "I Am India Shining": The Investor-Citizen and the Indelible Icon of Good Times. The Journal of Asian Studies. 2016 Aug;75(3):621–48.

80 Manmohan Singh was the Prime Minister of India from 2004 to 2014 during the UPA government.

81 The mission document of the National Rural Health Mission cites that NRHM was launched with the aim to carry out "architectural correction in the [India's] basic health care delivery system." https://nhm.gov.in/images/pdf/guidelines/nrhm-guidelines/mission_

there was a struggle to define what exactly such "architectural corrections" meant, and I must say that, quite down the line, many months later, an understanding of what was architectural correction meant was projected in a diagram of five circles denoting: communitization, improved workforce policies, decentralization, flexible financing, [and] professionalising management.

The important thing for that day and for today is that it was not based on the concept of making markets work for healthcare and on trying to explore alternatives to market-based reform. One of the elements where there was an agreement across all the stakeholders was on the need for ASHAs and for community engagement/communitization. The NHSRC[82] was to play a major role in interpreting and implementing this approach, but that was later, more in the implementation stage. In this seminar we are discussing one of these five areas of architectural correction: viz community processes.

Now, what are the roots of communitization? The meaning and design of community processes, like that of architectural corrections, opened up considerable dialogue, often very contested, amongst a number of civil society actors, political configurations, and the interpretation of politics by leading bureaucrats.

What are the origins of communitization? Actually, [incomplete] and I was very unhappy with this word. Bad grammar was not the issue. It came from the Nagaland health system. Nagaland had a very strong community-based system [in] that nothing could move within the community without the Panchayat and its decision. Even the doctor couldn't get a residence in the village. I later found that it was not necessarily so progressive. It was really a stage of evolution of social norms in that state, where the larger Naga identity for the state as a whole itself had to be forged, and each village had its own identity and autonomy. The communities were distinct and could distinguish each other by the scarf or beads or necklaces or headgear they wear. Each village was under a chief or headman and this autonomy had to be respected by the state. But it was not very democratic within, in the sense of individual freedoms and minority rights. Having said that, there was a very strong, positive element at that point.

The second, more important, source of understanding community processes, as Prabir captured very well, was from the Chhattisgarh experience. So, Ms. Jalaja, the first Mission Director, came over and travelled across the state. I was accompanying her along with the State Secretary in the car. [A] number of other officers were also sent to study the Chhattisgarh example, and they too travelled across the state. Their main questions were: "[The] government wanted to be strengthening public services, but what can we do?" They were actually confused about what should be done to make the public system work, because in the crude understanding of those days, everything that needed to be done was done. Mind you, that was a time when no recruitments [or] HR, no doctors and not even a single nurse had been recruited for almost ten to twelve years. It was almost complete stagnation, but Chhattisgarh had introduced a number of innovations, and the ability to even forge these new initiatives and what else can be done new was very important. So, there was a whole lot of discussion about Mitanins and about village committees and about village planning.

One major area of contestation in these discussions was the tension between defining the role of village committees. Was it making the government health system accountable or reaching entitlements to marginalised people? Or were village committees to undertake collective action in solidarity with health staff (e.g., in vector control)[83]?. Or were village committee and ASHAs to be low-paid extension workers, as Rakhal was discussing [regarding] contraceptives? While NHSRC tried to shape ASHA and community processes as a mix of these roles, another polar position was to shape

document.pdf

82 The National Health Systems Resource Centre (NHSRC) supports the development of policy and strategy via "the provision and mobilization of technical assistance to the states and in capacity building for the Ministry of Health [and Family Welfare." See: https://nhsrcindia.org

83 The text in parentheses is a clarification made by Dr. Sundararaman.

the ASHA as a system of commission-paid agents to generate demand to both public and private provider alike. They would start with incentives for promoting contraceptives and institutional delivery, but the package of such services and incentives would increase. In some sense, it was a system of communitization within a market-based understanding of reform. All these four approaches were in one crucible where they were encountering and contesting each other on that, and it's a dynamic understanding that emerges from this.

It's interesting that Jan Swasthya Abhiyan was very strongly in favour of community-based monitoring (CBM). In a particular period, in a particular major meeting, it seemed to be the only legitimate form of involvement with the NRHM that was acceptable to this network. However, in that particular form [of CBM], there was a consensus on JSA participation. So that was really the nature of things.

We must think of these processes not as designs that were set out in the beginning and cast in stone, but as programs whose design, scale and content kept evolving. When talking of scaling up, even the government was very, very hesitant. In the first program design, when the ASHA program was launched, it was not for a country-wide scale. It was only limited to high-focus states and to be limited to tribal areas in all other states. That was the original design. However, as it evolved, it varied. You can see that, in Tamil Nadu, as against 80,000 ASHAs that should be there, even as of today we have 2800.

I would like to say this. It wasn't quite the public health community, but repeatedly on every point, it was the politician who seemed to get many of the things right with regard to strengthening public health services. Because in those days [before 2004], the public health community, at the professional level—the level of the technical advisor and consultant—was very sold on market-based reform of healthcare reform. Some of them told me personally in these words: "You have got personal motives. Why are you doing that? You know very well that public systems don't work." So that was it. There was also the use of so-called "evidence". For example, there was the famous Ajay Mahal paper[84] which was cited to show that the public system is captured by the elite. We had a whole lot of intellectual expertise—national and international—that was cited to evidence propositions that food supplementation does not work for addressing malnutrition, and that vitamin A and iron fortification is more cost-effective than supplemental food. There was a whole lot of such "knowledge" that one had to contend with. Today, it's all right; the mainstream public health opinion has swung around and is more aligned with the NHM[9] on all its major measures of "architectural correction". But at that time, 2005 to 2009, it was really the politicians who were clear on that it has to be built around public services. So many actions did take place and were supported by political will. So, I think that there was a major role of that. There was a lot of scaling up of many successful pilots and innovations, which also would not have happened without political support.

Now, I really don't want to speculate on the future of CBM. Perhaps I've seen it in a very granular level to draw the large picture. In my view, community-based monitoring had inherent success and an inherent limitation. The more successful it was, the more cautious the state and government would become about expanding. The more superficial it was, the more it touched upon what local providers do and didn't deal with the larger questions, the more they had the space. Given a length of time, obviously people will start asking the right questions and putting the right pressure. And over time, as the CBM program grew in every state to a certain extent, then in one way or another, while paying lip service to it, its expansion was sharply limited.

Maharashtra, however, was an exception, and it held out and I think it holds out to this day. A lot of it is also due to the social capital that was brought in for that. I think that's an important lesson that we have. Because the leading organisation was able to network and have its own presence in program governance maintained under difficult circumstances, it could keep the game going. There are many efforts to shut it down, even in

84 Refers to this paper: Mahal A, Singh J, Afridi F, Lamba V, Gumber A, Selvaraju V. Who benefits from public health spending in India. New Delhi: National Council for Applied Economic Research. 2000 Oct.

Maharashtra state, but they were able somehow to persuade and squeeze out that space within the state government to continue on that. They were able to therefore show some data on improvements in service delivery that community-based monitoring had led to. But even they could not transfer that energy to the Village Health Sanitation and Nutrition Committee, so that continuation of CBM required in perpetuity the presence of the leading NGO.

The CBM was largely guided by the Advisory Group on Community Action[26]. But all other aspects of communization—ASHA, VHSNCs, RKSs, etc.—were largely guided from the NHSRC and its various avatars, in different degrees of collaboration with state governments. That's a long story as to how each of these other forms of community engagement evolve. We look at these as static, but if you were to track, i.e., the Common Review Missions (CRM)[85], over the years, what is being described and commented about VHSNCs would change. There was an evolution within each of these programs. There was an evolution of how you thought about it. Even our governance and management structures mature. Once the ASHA was in place, how you saw her relationship to the VHSNC was different from how one saw it before, and after that it changed again.

I've not been in touch with what has happened in the last three to four years. So, when you ask your last question of what it means for the current Prime Minister's program[86], I really don't have a clue. I need to get back there. The last two years, I have been distracted from this. I think I'm coming back to this. So, I will be looking back at some of these issues, but I do observe (somewhat superficially) that VHSNCs don't have that energy. Money has stopped flowing to VHSNCs due to the COVID crisis. Government may be using the COVID pandemic as a reason for getting out of VHSNC funding. A whole lot of funds which were allocated in the past to community processes have been the first to be cut back under the COVID pandemic. So, one can see that funding for community processes was conceded last and withdrawn first. We have a whole lot of issues that are related to that. So, I would stop here for now. And as Thelma pointed out, there's a long, 18-year and continuing journey, so it doesn't actually end at any one point. They didn't even begin at one point. So, it's very difficult to discuss it in 10 minutes.

Rama Baru: Thank you, Sundar. I just thought I would flag that we have time. What time do we end this?

Nerges Mistry: 4:30.

Rama Baru: I just thought I'd throw up some of the interesting issues that came up from all your presentations, and I think one that many of you alluded to is the continuity—the historical continuity—and the role of many community health initiatives that kind of was picked up at this point in history when the constellations, as you put it, came together. I also think that what Sundar said about Rashomon [style narratives] is very important. I think the recollection depends a lot on the positionality of each of these actors and the way they saw it. I think that is a very important aspect to it. There is also, I think, a very important issue that has come up in this Witness Seminar, which is of the state versus the Centre. So, while we see the NRHM was certainly a centrally driven process where there was representation from the states, the actual rollout of it and the differential sets of experiences in this process, I think, has not been adequately documented. It will be quite interesting for The George Institute to have even state-specific kinds of Witness Seminars or dialogues or whatever you want to call it. There was a very interesting point that Thelma made in passing, and I'm just going to pick that up. It was that even when you talk about PHM, NHM, the Human Rights Commission hearings, or the role of the JSA, that this particular formation or the coalition with other movements at that point in time were ideologically fractured. I think we need to see how that played out. It is not as if it was one ideologically cohesive formation,

85 The Annual Common Review Mission (CRM) is a monitoring mechanism under NRHM for the MoHFW to gauge progress on the scheme's interventions and identify those intervention that require mid-course modifications. See: The United Progressive Alliance (UPA) is a coalition of political parties in India formed after the 2004 general election. The largest party in the UPA alliance is the Indian National Congress. See: https://journals.sagepub.com/doi/10.1177/0974928416654367?icid=int.sj-abstract.similar-articles.1

86 Referred to the Prime Minister Atmanirbhar Swasthya Bharat Yojana (see note 33).

and there were tensions in it. I think there was also a sense of solidarity driven by this need to counter, as Sundar said, the HSR kind of process that was this the moment where one could contest that. So, I'm just sort of hoping that all of you would just sort of maybe come in with some thoughts on this, anyone who wants to respond, and maybe later on, Devaki, you can take it off one-on-one also.

Thelma Narayan: If I may just very briefly say that, since I'm still a member of the AGCA, actually, the budget hasn't decreased. It's actually marginally increased over the past three, four years, and the number of states that I've covered are also now more than what has been mentioned. It's not 22. It's maybe 24 or something. We have had a series of meetings, and we have been asked to revision the community action process, and we were having a series of very interesting discussions among the group in terms of what to do next. I think one of the issues that has come up is the need to include youth in the process much more proactively and systematically, because that was not necessarily done consciously in the earlier phase. We were just dealing with the community as a homogeneous group, and also to use the availability of technology, which now compared to 2007-2008, the penetration is much more. So, one can use IT much more effectively. Even our Zoom call here has been IT-enabled. Just one little additional point is that there have been at the same time other movements like the movements of Persons with Disabilities, which was never very active presence or voice in the health sector despite efforts taken up by the health movement. But the disability movement on its own has grown. There is the evolution of Disabled Person's Organisations[87] (DPO) that is very widespread, with the Rights of Persons with Disability Act[88], the CRPD[89] and all of that has gained reasonably strong ground. The community mental health movement also is growing, the gender movement, and of course, the LGBTQI movement[90] has also developed strongly. So, there have been several social initiatives and movements. I think the momentum that was generated and the structures that help people to set up differently enabled the 100 flowers—the 1000 flowers— bloom. They are blooming. So, I don't think in India you can actually snuff them all out. So, they are blooming in different ways. Now, if there was greater coming together, we would definitely push forward. However, when you look at all the health indicators, let's accept it, we are really on a very bad wicket. We have been successively going down. After the first ten years of NRHM, there were better indicators. I mean, there was definitely data that showed improvement. And now you look at the past five-six years, it's really on a downward trend. So, I think this is a time for people to come [together] across their fractured ideology and discuss it together. We are in a crisis situation now, health-wise. I mean, health and access to healthcare, both are in a bad situation. So, I think I'll just end with that.

Nerges Mistry: Rama, can I make a point? Thanks Thelma, that was very nicely said. What I would like to talk about is [something I] particularly remember—Mrs. Jalaja held this ASHA confederation meeting at the Constitution Club in about 2005 or 2006 when the NRHM had just started. I think that was a very good idea. I think she understood the importance of having the ASHAs from different parts of the state, different states to come together and share their experiences. I remember the ASHAs of Haryana and the ASHAs of Andhra Pradesh talking to each other. We formed

87 A Disabled People's Organisation (DPO) is an organisation led by persons with disability i.e., the board and membership constitute 51% of persons with disability. See: https://pwd.org.au/resources/disability-info/student-section/disabled-people-s-organisations-dpos/

88 The Rights of Persons with Disability Act was a legislation passed by the Government of India in 2016 to protect and secure the rights of persons with disability. This legislation is aligned with the Convention on the Rights of Persons with Disabilities to which India is a signatory. See: https://pib.gov.in/newsite/printrelease.aspx?relid=155592; Read the act here: https://legislative.gov.in/sites/default/files/A2016-49_1.pdf

89 The Convention on the Rights of Persons with Disabilities (CRPD), adopted in 2006 by the United Nations General Assembly, "clarifies and qualifies how all categories of rights apply to persons with disabilities and identifies areas where adaptations have to be made for persons with disabilities to effectively exercise their rights and areas where their rights have been violated, and where protection of rights must be reinforced." See: https://www.un.org/development/desa/disabilities/convention-on-the-rights-of-persons-with-disabilities.html

90 The LGBTQI movement in India had been fighting for the repeal of the draconian section 377 of the Indian Penal Code (IPC) that criminalised intimate relations between persons of the same sex. In 2018 the Supreme Court of India ruled section 377 as unconstitutional signifying a major victory for the movement. See: https://www.thehindu.com/society/its-been-a-long-long-time-for-the-lgbtq-rights-movement-in-india/article24408262.ece and https://qz.com/india/1379620/section-377-a-timeline-of-indias-battle-for-gay-rights/

groups and the ASHAs from Andhra Pradesh were quite different, on a different level altogether, and they said, “We do this, and we do that. We have managed to change community perceptions.” And the Haryana lady said, “If we do something of this sort, they will drop us in the well.” That was the type of bipolar [incomplete] type of ASHAs that you had coming together at these conferences. So, I think this sort of needs to be revived. The best way that people can get used to is like what COVID does: keep us apart and prevent interaction. If a movement or an activity can be started where these people at different levels of their functionalities can come together even virtually. If the times don’t permit, exchange and take strength from each other and their experiences, something positive would come out of it. I think this consolidation—as Rakhal talked about the federation—the power of two villages versus one, is a telling story. That is one thing that I would like to say.

Rama Baru: Thank you, Nerges. Anybody else?

T. Sundararaman: I think the point you made about states is worth reiterating. Now, we tend to make a general statement about the entire programme, but actually there is difference between states in how the programs were understood, implemented, and the way they went, even after the second phase expansion. Himachal, for example, came into the community processes and the ASHA program very late. Goa came into it even later. Uttar Pradesh had one particular take on it and took a long time to decide on whether to expand. In fact, they insisted that the state must be allowed to do local modification—state-level modification—of the material. They took the material and removed every little bit on gender that was there. There wasn’t much that was there on gender. At some point you had all sorts of things going on across states and sometimes it was progressive. There are very interesting things that you can get as feedback and learnings from one state to the other.

So, I thought that the cross-learning across states, as the earlier person also said [is important], because we really didn’t have and still don’t have adequate theoretical basis on which we explain how, without markets, you actually do reform. So, most of it is always learned from positive deviances and feeding that into, “Oh these are best practices working here, so it will work elsewhere.” That sort of logic, to whatever extent, is still the logic that NRHM follows. NRHM has a best practices seminar every two years, or every year, and its institutionalisation was a good achievement. But best practices are not necessarily replicable and sometimes we generalise too easily. There is a lot of variety in how it took place in the states and therefore how ASHA, the VHSNC, CBM and AGCA—how all of it—plays out. Again, I’m sorry [about] being unable to comment on developments of the last three years, I have just not been to the field at all.

Rakhal Gaitonde: Maybe can I just jump in, please? So again, I think I’ll just make a national, state, and field observation. So, just to recall these movements with different ideologies coming together. So, I remember this debate—and Thelma alluded to the fact that even within JSA, for example, there was a big discussion—, and in fact there was a meeting. I think Dr. Nerges would remember in Pune that Dr. Antia hosted [the meeting]. Sundar was also there who attended that meeting to actually thrash out this whole debate within JSA as it were, or at least to come up with something. I think I saw it in those days, as a very important debate and discussion that happened within the movement. But I just wanted to highlight one sentence that was there that came up. One of the persons there actually said that this is a very difficult decision time, because if we get involved, and get involved with the ASHA program—and at that point in time, the reading was that the ASHA program is doomed to fail because of all the various forces against it—, then we will never be able to talk about the community health worker program for another couple of generations. If we don’t get involved, then history will always look at us in hindsight and say, “You had an opportunity, but you never got involved.” I think this statement, to me, at least, remains as a sort of abiding dilemma that I think many movements and many groups faced in NRHM, and of course, personally, I’m very glad we jumped in and did as much as we could in the given space and so on. Moving to the states, I think you are absolutely right that there is a very sharp difference between the way the Centre saw it

and the way the states saw it.

I remember this discussion again with one of the Medical Officers who were writing the MoU [Memorandum of Understanding] at that time, and we were talking about what would be the outcomes. The Director of Public Health had actually suggested that the outcome of a community monitoring exercise or this whole community action, as we insisted on calling it in Tamil Nadu, was that he wanted to see OPD [Outpatient Department] numbers increasing. I said, "What is the logic between what we are trying to do and OPD numbers?" I mean, if you don't have a doctor, you're not going to have OPDs. So, unless you commit to saying… if I identify a vacancy, you will fill the vacancy—just to point out that the expectations of this were so widely different from what we saw in the Centre and the states. At the district level, I remember this meeting with one of our most dynamic Deputy Directors who is like the head of the Directorate of Public Health in a district. The Deputy Director was like, "Sir, you don't worry at all about anything. Just give my number to all your community workers and tell them to call me from the PHC that's not functioning, and I will immediately suspend the doctor or suspend the VHN." That was her solution to accountability and monitoring. We were like, no, that's exactly what we don't want. We want the process. We want to have a meeting. We want it to be sorted out in a democratic fashion. That takes me back to the first meeting we had in Vellore district. I remember this was hosted by CHAD[91] in fact—the Community Health Department of CMC Vellore—, and after explaining this whole idea about deepening democracy and all of those words that we use, we asked the community members and the NGOs who were there, "So, what do you think?" Of course, everybody said, "Fantastic", except one person, an elderly lady. I remember she said "Sir, this is very wrong." We asked why. She responded saying, "You know what will happen? All the blame will fall on the front-line workers when actually the issue is much higher up in the system and the only person who is actually coming to our village will be the person made to take all the blame." This was actually a statement by a person from a village. I think these are all just defining statements.

And I think apart from the Centre and the state, Rama and others, I think it's also important to look at who were the NGOs that actually did this. We had JSA stalwarts and JSA movements at the Centre but once you moved into the states and to the districts and to the block… I remember this discussion in Tamil Nadu, where we were having a state-level meeting where all the districts came. One district coordinator said, "In my district, there is no caste." Then, about six months later, at the next or two district-state level meetings later, the same district person said, "Sir, I want to say there is caste everywhere." This process of growth of NGOs, I think, is also something we need to look at.

Thelma Narayan: I just wanted to add a small point. Talking about the state, even during the COVID period, I have been involved, in fact. In Karnataka, maybe some of you know, they set up village COVID task forces[92], which included VHSNC members. It included ASHAs, but it was broader. It was a broader sort of coalition at the village level. We've had several meetings, and this was all on Zoom incidentally. It's amazing to see how these people, including your district program coordinators, all sorts of coordinators at district level, NRHM has created a huge human resource, which wasn't there pre-NRHM, and that human resource actually is playing a role. I've also been on Zoom meetings over this past year and a half with ASHAs in Rajasthan, as part of the AGCA. So, we were doing this dipstick method of trying to understand vaccine hesitancy. The AGCA Secretariat has actually done quite a stellar job of mobilising a

91 The Community Health and Development (CHAD) centre at Christian Medical College, Vellore conducts health activities supporting "approximately 200,000 in the rural, urban and tribal community areas of Vellore Districts." For more: https://www.cmcvellorechittoorcampus.ac.in/community-health-and-development-chad/

92 Karnataka established village-level task forces consisting of Anganwadi workers, panchayat members, and ASHAs to supervise the government's COVID-19 pandemic response efforts. The task force was responsible for reaching out to the public in rural areas conveying protocols from the district administration and the health department, and preventing the spread of misinformation, among other responsibilities. See: https://www.firstpost.com/health/coronavirus-outbreak-karnataka-govt-creates-village-level-task-forces-ropes-in-panchayat-asha-and-anganwadi-workers-8237691.html

lot of people. It's because of the relationships built over the period of years. The Secretariat staff, Bijit Roy and others, are really very experienced now. Over ten years, they've been working on this in a grounded manner. I was there at meetings with ASHAs, and I'm very fluent in Hindi, so it was very energising to see the way they were participating. They didn't need any sort of facilitation to get there. They were all saying whatever they had to say. They went on for a pretty long time on Zoom. They were all familiar with using their mobile, sitting in different villages and talking away. Now, Haryana ASHAs, I've heard some of them in the past few months and I would almost say, almost militant, very strong. I feel that maybe this is not a representative sample, but I would say that definitely the NRHM and this whole communitization process has set forth a social process which is gone much beyond. I was at those meetings in the early days. I was actually also a member of the ASHA mentoring group for the first seven years. I know what Sundar is saying. There was so much hesitation. Can we actually do this at scale in different states? I think the role some of us played is to give them confidence. Yes, it is possible. It's not some impossible dream, and it may not be perfect. But let's go ahead. We have some ASHAs in our SOCHARA[93] team right now, sitting here, and their level of ability in working with communities is extremely high. Without those women who have been trained as ASHAs, I don't think the community work which we are doing right now, on a daily basis, for which we have gotten hundreds of pictures of what's happening on ground [would be possible]. Their capacity is remarkable. Thanks.

Rama Baru: Prabir, are you there? I think you can have the last intervention before we wind up.

Prabir Chatterjee: I found it to be a very rich discussion. I could have gone on and talked a little more about structure. How so many of our Mitanin have become Mitanin trainers and so many of our Mitanin trainers have become block coordinators and so on and so forth. But I think the entire discussion has been very interesting and I hope somebody will follow up all these stories and look at everything that has happened. If today is really a place and the time rather like 2006 or the year before that, then we need new interventions in health. COVID has really shown us the weaknesses in the public health system. I think this is the time for those who are progressive to come together and start analysing the situation as they did 30 years ago and think about what we can do in the future. I leave myself at that. Thanks.

Rama Baru: Thank you very much. Over to you, Devaki and Misimi.

Misimi Kakoti: Thank you, Rama Ma'am. Thank you to all the participants on behalf of our team. Thank you for taking out time to attend this session amidst your busy schedules. We feel very privileged to have been able to have you all together here and listen to you all. It was very insightful and very enriching, and especially for younger researchers like us, it's very exciting and motivating. Like many of you have reiterated, this is perhaps the most critical part of health policy and almost all the participants that we have been having for these seminars have said that this is very extensive. For example, Dr. Thelma here has also mentioned that there's so many things, but you have very limited time. We can only cover, like, certain aspects very briefly. We're trying here to bring those pieces together from all of you and put it into a whole through the seminars. But if you feel you would like to have one-on-one sessions with us and tell us more about and add to your sections, then we'd be very happy to schedule sessions with you. Lastly, as you would be aware that the Witness Seminar process has a follow up also. So, first we are going to transcribe the conversation that you all just had, and then post that the annotation process will begin, where we are going to reference and annotate the programs or certain terms that you might have mentioned. Next, we are going to send the transcript for review to you all, and then you can edit or add more to your sections, and there's a final report, of course. And, meanwhile, as we go ahead with the timeline, if there is anything that you have, any literature or any materials that would help us strengthen the documentation that we are doing, we'll be very grateful [if you could share it], and thanks to you all. If there is anything else that

93 Society for Community Health Awareness Research and Action (SOCHARA) is an autonomous NGO resource group of community health professionals collaborating to achieve the goal of Health for All. For more: https://www.sochara.org/

anyone would like to say, otherwise, I think we can close the session. Thank you so much.

Devaki Nambiar: So sorry, I lost power. That's why I fell out. But really, I think if I may, I'll just add something very quickly. We have a few minutes. Is that okay, Misimi? I just wanted to say that we weren't really sure what we were doing when we started, and it's just been a complete revelation, even for someone like me who's sort of been around for the past decade and a half, to just really hear these stories. It's been very moving, actually, which I was not expecting. There are just orders of magnitude of follow up, which I'm a little intimidated by, but I think it's very important to document these histories and stories. So, I thank you really, very deeply for giving this your time, and I hope we can be a bit more greedy with it as we go forward. There is an effort to not only do this documentation, but also for people who are more comfortable to do one-on-one documentation, as Misimi said, including you but even people you think we should try to chase up, which we're happy to do. Beyond this, I wanted you all to know that we, at The George Institute, are really seriously now thinking about some of that state-level follow up because of that Centre-state difference. This has all been done just on sort of internal resources, just because we really wanted to do it. We're going to see if we can mobilise funds to deepen the work here, and there's some interest in doing something similar internationally as well. There is some momentum around really pushing forward the cause or reinvigorating discussion around social participation and health as that handbook[94], as you know, that's come out. But I think at that level, things become very generic. And I think even at WHO there's this very keen awareness that granularity is what community action is about and that we have to find ways to learn about those and to share those stories. So, there will be some emphasis in 2022 in deepening but also widening this work. So, we'll be pestering you in various combinations about this, I'm sure. But anyway, thanks for giving up your time for this. And as Misimi said, we'll be in touch. We're deeply grateful. Thank you.

Rama Baru: It was great seeing everyone. Let's hope we meet in person soon.

Proceedings end.

94 "Voice, agency, empowerment - handbook on social participation for universal health coverage" was published by the World Health Organization in 2021 with the aim of sharing pragmatic guidance on carrying out social participation for health efforts. The handbook can be accessed here: https://www.who.int/publications/i/item/9789240027794

Annexure

In-depth Interview with Dr. Mirai Chatterjee

Devaki Nambiar: I think you can probably imagine with the names that I am giving you, where people sort of slotted themselves. And then they...it's sort of...the way the people are talking about it, there's, you know, "I was at this meeting, I was at that meeting...This and that happened." That is sort of the part of the story they want to tell...because it is a very long period, actually. That's the other thing. And I think for you, the emergence period is, you know, you were there in some of those, earliest of conversations, and I think there were some of these linkages you were drawing and the context you were bringing on one hand, that were very local and specific, through the work that SEWA[95]—the organising that SEWA has been doing. And then also some of the global kinds of conversations, perhaps that you were involved with as part of CSDH—I don't know, you could bring that in. And then... I think for us that's really sort of the starting point, and then we kind of leave it to you, where you want a... you know, then the models evolve... the government's change, you know. Some of the design changes, the state variation happens. So, if you'd like to weigh in on that, that would be great.

But I think that the first initial moment and then now, the sort of stock-taking and what you feel is the legacy of that organisation and that coming together, that advocacy. And what the takeaways would really be globally. Because I think, you know, for an institution like SEWA, and an organiser like you, that global view is always something that you have had, you know? So, a little bit of that takeaway. If you have some thoughts on that, that would be super. And I have talked way too much. So those were my initial thoughts. Misimi, do you—okay—have anything to add? No? Okay.

Mirai Chatterjee: Should I sort of start, and then you and Misimi stop me at any point?

Devaki Nambiar: Yes, we will take notes and we will stop you. Okay.

Mirai Chatterjee: Yes, if something is not clear, or if I am going off in another direction that is not really fitting in, let me know...because I thought what I would do is to start with SEWA.

Devaki Nambiar: Yes!

Mirai Chatterjee: I can get started on why we took certain paths, which may be different from some of the experiences of colleagues with whom we all work closely. Alright. So, let me begin at the beginning. I think community action or community engagement on health started from the point of being a national union. Although when SEWA started in 1972, it was not a national union as it is today. It was a small union of informal workers. And when I joined in 1984, there was still just a couple thousand members. I believe there were eleven or twelve thousand members—hard to imagine now because SEWA's collective strength this year has crossed 21 lakhs. 2.1 million members in 18 states.

But in those days, it was only Gujarat and a handful of members. I think the reason I was hired was because, again and again, from the workers' perspective—informal women workers' perspective—what was coming out was that, as the women put it eloquently: "Our bodies are our only assets." They said: "Please help us remain healthy because whatever we earn is going in doctors' bills and out of pocket expenditure." And yet, they had very low levels of health literacy and information about their bodies. So that's really how we started our journey on community action for health. It was actually less about community and more about starting where the members were—the women workers themselves.

As a union, our paid-up members are most important to us. And then of course, the members' actions have a ripple effect in the community, as members are embedded in urban and rural communities, obviously. But we started with our members. And the reason we started with community-based primary health care program

95 Self Employed Women's Association (SEWA), formed in 1972, is a trade union of women workers within India. SEWA supports poor working women in forming member-based organizations to help secure their economic, social, and legal rights. For more, read https://sewabharat.org/sewa-movement/

at all was, as you probably recall Devaki, in the 70s-mid-70s, before I joined, around 1977, SEWA Bank[96]—which was up and running as the world's first women's urban cooperative bank giving financial service to informal women workers—SEWA Bank did a study in, I think, around '77 to see what is happening to their loanees who were not returning their loan payments on time. And it was a rude shock because they found the major reason was sickness: of self, of family member. And a further shock came when we found that, out of 500 women who were not paying regularly, mainly because of sickness of self or family member, 20 had died. And 15 of those 20 had died in childbirth.

So that really forced SEWA's hand to begin working, not just among the members, because you know, you have to take a holistic, integrated approach—a community-based approach—to this issue. And that's really how we started our journey. You could say in a sense, by the very nature of the organisation we were, which is a membership-based organisation, a union, community action was part of the DNA. [It] was not something that we had to think, "Oh, that this is a nice thing to do". It was the thing to do. So, I think I wanted to put that out there.

And also, from the lens of a worker. So, I mentioned the situation of maternal mortality. So, obviously we began working on maternal health, safe motherhood, ensuring that no women died in childbirth among our members and their neighbours and friends. But, also, through the occupational health lens, because we were interfacing with women not simply as citizens or as mothers, but as workers. And I think that also influenced the direction, both of our programs in primary healthcare, and also the way we went about things. Why? Because we went not only to women's homes, but we went into their workplaces. We went to the tobacco fields where they worked. We went to their homes, where they were rolling beedis [thin cigarettes] or agarbattis [incense sticks]. We went to construction sites where they were falling and injuring themselves, and [we] understood from their perspective how we could support them. So, that's kind of the first set of issues that I wanted to put on the table. And if you and Misimi would like to probe in anymore, or if this doesn't seem clear, please give me a shout.

Devaki Nambiar: Related question I had to that Mirai behn, maybe is: were there sort of—perhaps a slightly different design—, were there other experiments that you were sort of watching or that were a frame of reference somehow? Conversations happening across the union with other groups, anything like that that you think is important.

Mirai Chatterjee: Absolutely. I mean, there have been many organisations, you know, when I was still a student in the 70s and early 80s… already several models of community action had been tried and tested in India. And one that influenced me in the early days was definitely the Jamkhed experience[97]. I went to Jamkhed myself, and witnessed how community engagement, community action happens. Then with Dr. Antia and his team, all the Mandwa[98] experiments… [I] wouldn't call them experiments, they were really very solid action. There were some like this already on the ground, up and running, which I had the privilege and honour to witness, read about, visit and have discussions with those who were leading.

So, definitely, those kinds of ideas influenced us. And, so, we…our focus was less on, "Let's build a hospital for SEWA members", and more on, "How can we engage with the members and their families

96 Shri Mahila Sewa Sahakari Bank Ltd. (SEWA Bank) seeks to provide access of appropriate and adequate financial services for "socio-economic empowerment and self development" to poor women workers women involved in the unorganised sector. Read more at: https://www.sewabank.com/introduction.html.

97 The Jamkhed Comprehensive Rural Health Project (Jamkhed CRHP) of 1970 was started by Rajanikant Arole and Mabelle Arole. It is a project that initiated the bringing of health services closer to the community and service integration, among other activities. Its approach and experience of community participation has influenced 1978's Alma Ata declaration on Health for All. Source (read more here): Perry HB, Rohde J. The Jamkhed Comprehensive Rural Health Project and the Alma-Ata Vision of Primary Health Care. Am J Public Health. 2019 May;109(5):699–704.

98 The Mandwa experiment was initiated when a team from the Foundation for Research in Community Health (FCRH) shared with women residents of Mandwa strategies for treating common medical issues. These residents subsequently were worked to achieve key health targets. These successful efforts influenced health planning and policy for the country. Read more here http://www.frchindia.org/The_Story.html

and their mohalla [locality] people and village people—even if they are not members—to discuss issues of health that were close to them?" Be it their occupational health, be it their maternal health, children's health—whatever came up organically. Because like many of our other colleagues, for us an article of faith is you start where people are. People are in the centre. In our case, women. I mean, if the women said, "This is something we need to work", then we began to organise them into groups or into health literacy sessions, health education, and so on. So, yes, certainly, those early examples of community action from different parts of India were an influence. Because I was a young public health worker, I had read about them, as I had said, I had visited. And then I think what was also instrumental was having a cohort of people who were working on similar approach to primary healthcare with people in the centre, with community action in the centre, and I'd like to specifically mention Medico Friend Circle[54]. Because in the early days, we were active in Medico Friend Circle...used to go to many of the meetings, conferences, discussions, thrashing out of issues.... So rich.

And similarly, the Voluntary Health Association of India[99] had at that time very active chapters. And I'm sure some [of them] are still active. But, at that time, the Gujarat chapter, Gujarat Voluntary Health Association, was very active, and we were very active in it. I think at a certain moment even I was the President of it, if I remember correctly... for some years. And that gave us a forum to listen to others, learn from others, contribute...how are other people doing things? And the other people included SEWA Rural Jhagadia ... Dr. Daxa Patel[100] and people, and her team. Unfortunately, she is no more, from ARCH[101]. So, I think you know, those early days, the richness of those interactions and exchanges of the 'how to' part and the different experimentation was certainly very helpful and gave us lot of pointers. I think another watershed moment was the whole organising and work up to the Cairo conference, the International Conference on Population and Development[102]. Because as you both well know, until then, pretty much India's public health program was in fact a family planning program...because I'm talking about the early '80s still. And I think there was a lot of organising led by Ena Singh of UNFPA, Devaki Jain, and Devaki Jain[103] was a long-time friend of SEWA and Elaben...[104] So, I got invited along with other colleagues to many workshops and we thrashed out a lot of issues: what is it we wanted to see at ICPD? What did we want that Cairo conference[102] to do in terms of making a difference and listening to women's voices and girls' voices and so on? I think the workup, which was two years, if I remember correctly, before the Cairo conference was really important. And then the conference itself was very instructive because for the first time I was meeting women from all over the world. I hadn't been to such an international conference earlier. And these were women who shared the same concerns and some of those were unique to their contexts, but they also talked about organising women, what worked in communities, what kind of action, so all of that added to our own understanding and approach. And one of the things that happened after the Cairo conference was—some colleagues might have already told you—that a formation called Health Watch was formed. Our government was actually really instrumental at Cairo. I would say that. On the floor of the house. I mean, it was... we

99 The Voluntary Health Association of India (VHAI) is a non-profit health and development network. VHAI supports innovative grassroots initiatives on health and development in facilitating active community participation. Read more at https://vhai.org/about-vhai/

100 Dr. Daxa Patel was the founder of ARCH and Friends of ARCH. See: https://www.friendsofarch.org/

101 ARCH is an organisation that aims to work with "tribal and downtrodden villages of India" with portfolios in education, forest rights and health. See: https://www.friendsofarch.org/

102 The International Conference on Population and Development (ICPD) was convened in 1994 in Cairo, Egypt by the United Nations Population Fund (UNFPA) and the Population Division of UN Department of Economic and Social Affairs. The conference introduced the 'Program of Action' which "emphasized the fundamental role of women's interests in population matters and introduced the concepts of sexual and reproductive health and reproductive rights." Read more here https://www.unfpa.org/events/international-conference-population-and-development-icpd

103 Devaki Jain is a feminist economist who has had a strong influence on the women's rights movement. See: https://www.tribuneindia.com/news/reviews/story/memoirs-of-feminist-economist-devaki-jain-154087

104 Ela Bhatt is a lawyer and social activist who helped establish the Self Employed Women's Association. She is presently a member of the global group called the Elders.

felt proud to see the way the Indian government fought tooth and nail for the women and girls at the global forum. We were almost surprised how much. And fought hard against the US and other countries, who were against abortion or other aspects of women's reproductive rights. There we put in a stellar performance, and civil society and government joined hands and worked together as one. It was actually—as you can see, from the way my face lights up—, it was actually one of the most exciting experiences I would say in my public health career. I would say that. And it led to Health Watch[105] where we said that okay, we had all had this great partnership going with the government, at the global level. How about back home? Hold on to the commitments.

So that was what Health Watch was about. And there was a lot of interaction with both policymakers but also interfacing a lot with the grassroots, bringing a lot of things from Cairo to the grassroots...grassroot issues. And possible avenues for action to Health Watch and to the government and so on. And I believe that those kinds of interactions were actually very critical to get the National Rural Health Mission[9] off its feet. Because I don't think we would have had community action if all these interactions had not happened. I mean, they just kept building on each other, layer on layer. And I think also because our government saw that, you know, there was something worthy here—some worthy experience of organisations like ours. All of us together. And there was 70 of us at Cairo (civil society organisations).

I do believe that it wasn't just one or the other, it was the interaction of community action—both, if you will, bottom up and top down. What a friend later described as the nutcracker approach, [wherein] both pressure from both [sides]. In between, there have been several other important meetings and conferences of the World Health Organisation, and I think slowly—slowly, because you know, all of these levels are interactive, and we know that it is hard to bring change on community action locally, at state level and nationally unless there is at least a gentle nudge from above (WHO and so on). It always works like that. All these things working together in tandem. So, in a lot of meetings in WHO, one pushed for primary healthcare, community action, bottom-up approach, people in centre, etc. And then I think another watershed moment was the Commission for Social Determinants of Health[106], as you rightly pointed out. And I think it was a watershed moment because I think in its essence this commission noted, understood in a very kind of basic way—almost a gut-level way—that without community action and without social determinants of health, we weren't going to improve health of communities all over the globe and we weren't going to close the equity gap. Or inequity gap. We weren't going to be able to address inequity unless we had community action, citizen engagement, women and communities organising, and so on. And I think if you read that report, you know, this comes out—jumps at you from the pages. Because all the case studies and all are on this. It's what communities have invented bottom up to improve their own health, largely. Whether it is to do with microfinance, gender equity, or livelihood, or whatsoever. So, I think that was another watershed moment. Again, top down, it came from Geneva but then SEARO also got really active and engaged in this. There's a case study, a whole booklet was done on how this community action—the SEWA approach—happens. So, I think all of those really helped to further the agenda and the understanding. But I still think it's been a journey of ups and downs and coming to the AGCA (the Action Group on Community Action)[26], and you've already interviewed many of the people there. I think all of us together pushing forward for community action was really powerful and I think it

105 The 'National Rural Health Mission' (NRHM), launched in 2005 by the Union government, has proposed increased public health financing as well as strengthening of rural public health facilities. Given this context, JSA's health rights advocacy evolved, working to influence NRHM in a pro-people manner and simultaneously evaluate the extent to which the proposed enhancements were truly being brought to life. To achieve this, the collaborative NRHM and People's Rural Health Watch was launched in seven states between 2006 and 2008. Health Watch played a key role in rights-based advocacy in the design of RCH-II and NRHM. See: https://archive.phmovement.org/en/node/3051.html

106 The Commission on Social Determinants of Health, created by the WHO in 2005, helps countries and global health partners address the social determinants of ill health and related inequities. For more, read: https://www.who.int/teams/social-determinants-of-health/equity-and-health/commission-on-social-determinants-of-health

got us further than it would have, were we working on our own.

I think looking back, one of the things that I think was perhaps a sort of mistake on our parts in AGCA was that again it was top down. The monitoring systems we prepared were prepared by NGO leaders. They were not prepared by the local people themselves. So, I was—honestly sometimes I really felt a bit alone in the discussions because all these red, orange, and green signals…you know, it's quite complex for people to understand, particularly women and others who don't. All these things seem very nice and easy to ask, but they tend to be abstract. That's exactly the word. So, I think looking back what I would have liked was—and I should have also raised this more strongly—is that we go back to communities and ask them how they would like to monitor, the kind of services—and it's not just about monitoring— what kinds of community action. You know, community-based monitoring is only one piece in the whole continuum. Community action means that communities act together to improve their own health, on the social determinants scale—cleaning up the place, water, nutrition. All of that.

Monitoring being one part of it, planning with the local public health authority. So, there is a whole spectrum of action., and I think we focus too much on monitoring. And I think that was a strategic error in retrospect, because it really got the government's back up. They thought we were just here to criticise, which was never the intention. It was to empower communities. But I think you've to empower communities to act along the entire spectrum. And I think because SEWA is influenced by Gandhiji's[107] thinking, we believe firmly [that] we start with ourselves. Rather than asking the government to do xyz, we start with ourselves. We find out about our own bodies, we sit together, what kind of action kind can be taken to make sure that no child is malnourished in our village or our mohalla? What can we do? Can we all give a fistful of grain to that widow who is not able to feed her child properly? What, you know, what about the Panchayat's role? What can they do to clean up the place and so on and so forth? So, I think, you know, in retrospect, it would have been good if we had started like that, and certainly we start in that way and then build up from there. And see what works for local people. And also, immediately people are not in a position to do monitoring and planning—you start monitoring the ASHAs and all nurses, they might stop coming to your village. So, it has to be done in a very strategic manner.

My experience shows that community action, if it is done in a manner that is seen to be collaborative, in partnership, then slowly all these barriers and negative perceptions—are you coming to check up on us—all those slowly diminish. I remember when we started doing capacity-building of the Village Health and Sanitisation Committees and Mahila Arogya Samitis—the local health committees—there was so much resistance, from the ASHAs, but mainly from the ANMs and the MOs…medical officers who were benefiting from this, taking money and buying new curtains or whitewashing the walls or doing whatever, which is not supposed to be what this money is for. And they thought these people are coming to check on us, they'll complain about us, so we had to do a whole lot of constructive action with them, like for example helping track malnourished women and children, working with people to make sure they came for the wellness day (Mamta Divas[108]), and so on. When it was win-win for both, then slowly we could undertake more community action. So, that's one thing I think we learnt, somewhere along the way. Not that we shouldn't critique, not that we shouldn't raise our voices against injustices, but I guess I am arguing for a balance. Because it is finally not useful to local people. They want those services. And if I may give one little story which really is always on my mind is that we found that one doctor—Medical Officer—in a Primary Health Centre, and I'll tell you

107 Mahatma Gandhi, also called Gandhiji is a very prominent Indian leader who played a very critical role in India's freedom struggle and nonviolence movement, leading to India's eventual Independence from Britain in 1947. He inspired generations of pacifists and nonviolent activists including Martin Luther King Junior and Nelson Mandela. See: https://www.mkgandhi.org/africaneedsgandhi/biography.php

108 Mamta Divas (Health and Nutrition Day) is organised every month as part of the preventive and promotive outreach services for antenatal care and post-natal care initiated by Gujarat's Department of Health and Family Welfare. See: https://nhm.gujarat.gov.in/mamta-abhiyan.htm.

the one as well...called Gangad in Dholka taluka[109] of Ahmedabad district. He was not attending the PHC; instead, he was running his private clinic and he would tell people to come there and then charge and he was being paid by the government. We found this out and we sat, discussed. The women said yes, we need to take action on this so on and so forth. We spoke to the District Health Officer, and we took it forward, all the way up to the Health Commissioner, Dr. Amarjeet Singh, at the time. And he was about to be suspended and then the village leaders came running to us saying, “Please do not do that. Do not do anything without our permission, like this, even though some of the women said it was okay to do. Because at least we have a doctor in our village. He lives here. He cares for us. We don’t want him suspended.” So, I had the awkward situation of calling up the Health Commissioner in Gandhinagar and telling him, “No, no, no, sorry, don’t do anything.” He said, “Why? I’m just about to sign.” These are the kinds of experiences that we have, that if we don’t listen to communities, we end up shooting ourselves in the foot and more especially you know, affecting them.

Dr. Mirai leaves to attend another engagement.

Devaki Nambiar: One thing I was wondering—and I will ask when she [Miraiben] comes back—was sort of the idea of working in collectives or in Self Help Groups[110], and the connection between the Mahila Arogya Samiti[11] kind of idea how... in some ways it is meant to recreate the modality of a Self Help Group, but as she was saying Self Help Groups have a different kind of genesis and goal...oh, you are back.

Dr.Mirai returns

Mirai Chatterjee: I am back. So, yes, I guess ours is more the self-help approach. But, I mean, not letting the government off the hook, but just to give you examples, sometimes you have to be very careful. We are not embedded in these communities. So, I may think whatever I want to think—this is a suitable action to take—, but the question is, you know, they need to take the action, own it, and it has to fit in with their lived experience. So, there have been many such. But this one I always remember. The other thing that I wanted to say is that I think one thing that we’ve learnt all these years is that a strong grassroots base, organised base, and of course having a mass base of 20 lakh women, helps. And having the experience of working with public health authorities at the grassroot level makes them more amenable, not only to listen to us for ideas on community action, but also taking some of our ideas and incorporating them into public health programs. Of course, the biggest example is of ASHAs. I am sure Jamkhed and Mandwa and everybody else informed that decision. But the chain of low-cost pharmacies[111] that we have been running, as you know Devaki, for many years, that partly at least informed the whole Jan Aushadhi program[112]. And similarly, the Rashtriya Swasthya Bima Yojana[113]—I remember the policy makers came to us, spent two-three days studying

109 Each district in India is divided into sub-districts, referred to as Taluks or blocks.

110 “A self help group [SHG] is a village based financial intermediary committee usually composed of 10-20 local woman. The members make small regular saving contributions for a few months until there is enough capital in the group for lending.” See: https://sewainternational.org/women-empowerment-through-shgs/

111 The Shri Gujarat Mahila Lok Swasthya Sewa Sahakari Mandali is a cooperative set up in Gujarat and promoted by SEWA, providing accessible preventive health information/education and low-cost curative health services to women. For example, it runs low-cost pharmacies and manufactures Ayurvedic medicines at affordable prices, also undertaking other health action. See: https://lokswasthya.org/access-to-low-cost-medicines/

112 Pradhan Mantri Bhartiya Janaushadhi Pariyojana (PMBJP) was launched by the Government of India in 2008. Under this scheme, pharmacies known as ‘Janaushadhi Kendra’ were started and supported to make generic medicines available and affordable for the general public. See: http://janaushadhi.gov.in/pmjy.aspx

113 The Rashtriya Swasthya Bima Yojana (RSBY) or the National Health Insurance Programme was a programme of the Ministry of Labour and Employment, Government of India launched in 2008 to provide health insurance coverage to families which are recognized by the government as being ‘Below Poverty Line (BPL)’. It has now been subsumed under the Ayushman Bharat scheme operationalised by the Ministry of Health and Family Welfare. See: https://www.india.gov.in/spotlight/rashtriya-swasthya-bima-yojana. and 66

the entire model, and adapted it for the national level. And that itself got morphed into PMJAY[114] as you know.

I think the one difficulty that we have seen is that government and policy makers come, they get excited by what they see as an example of community action, and they try to scale it up. But they don't understand the nuance and the spirit of these things. That they must be community-based, there must be community ownership and accountability. It is not [a] one-size-fits-all that you just roll out across the country. Doesn't work like that, and so there were huge issues with RSBY and, to be honest, we had warned them in advance. We said that if you don't have a community action component, rather like we have AGCA, informing you, being your eyes and ears and intelligence from the ground, helping you tweak things, then your implementation will be difficult. "Oh, we will see to that later, later", but the later never came. And the RSBY ran into a couple of stormy seas and then morphed into PMJAY, and in the PMJAY, the same mistakes! You know they've had the few consultations, they even ask us to write papers—how do you do this, how do you do that, how do you bring in the community—, and at the end of the day, basically they do what they want to do. Which is of course their prerogative. But if you consider our experience along with our other colleagues who you've interviewed, what we learn is that if you don't engage with communities from the beginning—right from the drawing board stage, from conceptualisation, design, what should be the implementation mechanism—, then afterwards you falter, and then afterwards tweaking those things and putting them right becomes a herculean task, because the systems are in place already. So, you know really from the conceptual stage these things have to be hammered out, and that is where I believe that community-based organisations, CSOs, Self Help Federations, women's voices are critical.

Another one or two things I wanted to say which I missed saying earlier was that, when I spoke about how community action is embedded, one is the union, but the other is cooperative. And as you know, about 32 years ago, we registered a health cooperative, Lok Swasthya[111], where the local Dais[115], traditional birth attendants and local women who were trained by us to be health workers, were the shareholders. So, they were the users, owners and managers of their own cooperative. So, in a sense, community ownership, community action, [is] embedded in the forms of organisations you choose. If you have membership-based organisations like unions and collectives, like cooperatives, you compulsorily have to do things in a collaborative, inclusive, democratic, transparent manner, and in a manner that puts their ideas at the centre. Because the boards—the elected boards—are of the people themselves, of the women… In our case, themselves. So, they are the ones who say Nahi, aisa nahi karo [don't do it like this], do like this, don't do like this, we need this, we don't need that, do this action, don't do this action. So, I think I guess I am saying… I am not saying other ways are not conducive, but I am saying our experiences is that, when the structure of your service is membership-based—is democratic and transparent—, then community action is part of the cause… it's part of the DNA, as I keep saying. I just wanted to make that comment.

I guess the last thing I wanted to say was about the National Advisory Council[116], which as you know, I had served on from 2010 to 2014. There also we tried very hard to bring in the whole community-based approach, community action approach, consultative approach with citizens and so on. Unfortunately, perhaps with the exception

114 Pradhan Mantri Jan Arogya Yojana (PMJAY), popularly known as 'Ayushman Bharat', was launched in 2018 to deliver comprehensive services across preventive, promotive, curative, rehabilitative, and palliative care. PMJAY has two components: the Health & Wellness Centres (HWC) (see note 65) and the National Health Protection Scheme which provides a health insurance cover of Rs. 5 Lakhs per year to over 10 crore "poor and vulnerable" families seeking secondary and tertiary care. Source (and read more at): https://www.india.gov.in/spotlight/ayushman-bharat-national-health-protection-mission

115 Dai refers to a traditional birth attendant.

116 The National Advisory Council (NAC) of India was a body set up by the first United Progressive Alliance (UPA) government to advise the Prime Minister of India Manmohan Singh on policy legislations. It is a committee consisting of civil society members, ex-bureaucrats, lawyers, and academics, acting as a bridge between civil society and the Indian government. See: http://www.allgov.com/india/departments/ministry-of-youth-affairs-and-sports/national-advisory-council-nac?agencyid=7592

of the Food Security Bill[117], some of these ideas which were embedded in our recommendations on universal healthcare didn't really go forward at the time. But they are there. They are there in the Planning Commission's[118] ideas and they are there in the National Health Policy 2017[119]. So, I guess good ideas don't go away, they are embedded. But the question is: how do you actually implement this on the ground? That is the real test. We can all agree to everything. Then how do we do it and what is the impact on individual women, their families, communities? Those are kind of questions that are all works in progress, I think. I will stop here because I have been talking a lot. So, if you guys have any more questions for me, please fire away.

Devaki Nambiar: Misimi, did you want to? I don't want to hog the questions. I do have some, but…

Misimi Kakoti: No, Devaki, you go ahead, you had a question…

Devaki Nambiar: Yes, I mean, I think this implementation question I was just thinking Mirai ben about where you started. At the beginning, where you talked about these types of interactions and the coming together and sort of the '70s and the '80s of implementers, and there was this very earnest kind of effort across different types of civil society organisations and membership-based organisations to do learning together. Do you see…I mean, are there sort of efforts in that direction? It seems to me that that is less the case now, am I wrong about this? Is that the kind of thing… because I think in what we have seen over the past decade or so that I have been trying to work on is that there is great ideation, and there is great conceptualisation, and the principles are solid and…but somehow those nitty gritty pieces—the variation, the context—those types of conversations are perhaps…do you think that is happening less now or is it happening in a different way? Or we're somehow [incomplete]

Mirai Chatterjee: I am trying to think. I think there are several forums where this is happening. One being the AGCA to some extent. But I think COVID put an end to a lot of this. I think the last two years and not meeting face to face is a major issue. I mean, how many webinars can you do? Still, we had many meetings online, and there have been several webinars where these kinds of issues have been thrashed out. So, I think there is engagement, definitely. But I think we have become a bit scattered since COVID. You know, first involved in emergency relief and firefighting and still not totally out of the woods on that one, as we know. So, I am hopeful that again we will interact, interface, exchange, develop strategies, if that was your question. One thing we did do around 2018-19 a lot was have several small workshops—you may be aware—on the Universal Health Care. One national one, then we had one in Rajasthan for western India. We had an excellent one in the northeast, in Meghalaya. We had one in Madhya Pradesh for central India where it was very consultative, it was with citizens. With women mainly, asking them—and men, but a lot more women—, asking them what kind of community action [incomplete]. Documenting the richness of the community action that they were already undertaking, that nobody even knew about. Especially in the northeast. So, I think some of that has gone on and I am hopeful especially with the current Lancet Commission[120] that we will have an opportunity to jump start that again, once things open up a bit. Because that kind of conversation you can't have like this on Zoom. Certainly not with people at the grassroots with

117 The National Food Security Bill was introduced in 2011 and launched as an Act of the Parliament in 2013. It shifted the approach to food security from a welfare-oriented measure to a rights-based approach by providing legal entitlement to people for subsidised food grains. See: https://nfsa.gov.in/portal/nfsa-act

118 The Planning Commission of India was set up in 1950 to formulate the Five-Year Plans, among other planning responsibilities, and was dissolved in 2014 (and the Five-Year Plan system was terminated). NITI Aayog was constituted in its place to continue providing 'strategic policy vision' for the government along with supporting monitoring and evaluation of government programmes and research and innovation activities. Read more at https://www.niti.gov.in/ and https://niti.gov.in/planningcommission.gov.in/docs/aboutus/history/index.php?about=aboutbdy.htm

119 The National Health Policy 2017 focuses on universal health coverage emphasising "universal access to good quality healthcare services without anyone having to face financial hardship as a consequence." Read the report here: https://www.nhp.gov.in/nhpfiles/national_health_policy_2017.pdf

120 The Lancet Citizen's Commission on Reimagining India's Health System was constituted in 2021 to formulate a roadmap towards achieving UHC in India through participatory engagement with health care actors and India's citizenry. See: https://www.thelancet.com/pdfs/journals/lancet/PIIS0140-6736(20)32174-7.pdf

spotty internet.

Devaki Nambiar: So, I think then, I mean as the world sorts of hobbles back, hopefully, we have some reprieve for some time. I think to some extent the legacy of COVID is also the one that reminds us of the importance of citizen action and community action today. We address even emergency situations. So perhaps some documentation that has to happen on that. But from the Indian case, what would you say are some of the lessons for organising and community action globally? You talked on one hand about just having that range right. The membership-based types of initiatives, the Watch, the monitoring—those kinds of things. Could you just talk us through...

Mirai Chatterjee: You mean during COVID or generally?

Devaki Nambiar: No, in general. I mean, now that we have the context of COVID as well, but in general.

Mirai Chatterjee: So, I think the pandemic taught us that the communities are incredibly resourceful. We always knew they were insightful and resourceful and that there were courageous local people. But, I think that has come out really clearly. So, I hope that policy makers and others of us will understand now finally that really the place to start is with people in the centre. Particularly the poorest and the most marginalised in our country because they provided yeoman service at a personal cost and bravely. Their families told them not to step out. They stepped out, saved people, helped people. I mean, it really was a remarkable effort across the country and across the globe actually, everywhere. Because what we saw was that governments could not respond or were not able to respond fully or themselves were overwhelmed. That is where everyone stepped in.

Devaki Nambiar: Misimi, one last question—I am noticing time, it has just flown as usual—, which is about the kind of watershed that ICPD was at kind of at the highest level. CSOs and government working shoulder to shoulder. Is that needed, or?

Mirai Chatterjee: I think people have understood better, I hope, I think so, that you know, with support, communities can undertake a whole lot of community action for health. Starting from planning, then to actual implementation, linking with government public health authorities and even private, referral, providing mental health support, because no one else will trust outsiders. They will trust only their own ASHAs and health workers. And then all the way up to monitoring and evaluation. I think there is an understanding, I hope so, that these things now are able to be done by local people if there is support in capacity-building and ongoing interaction, and there are certain things that communities can do better than anybody else and there are certain things they can’t do. So, you know, identifying and seeing where we can come together and build on our mutual talents and insights and resources. Government on one side and people on the other. I think that understanding is there. I am hopeful. And I am hopeful because I think the Lancet Commission is also an opportunity. Whether our government wants to listen or not and other governments want to listen or not is another question. But at least it will bring out the evidence in a scientifically rigorous manner. It will have voices of communities, because the main focus is the citizen engagement, as you know very well. So, it will be both qualitative and quantitative, voices, concrete examples, case studies. So, I think those would enrich not only the report, but enrich our collective knowledge on how the communities can be engaged.

Devaki Nambiar: Yes, I think we are all very hopeful about that. I am wondering Mirai Ben, I know that there is the... I mean...one is to think about ICPD and how there was... I was just telling Misimi...there was this sort of standing shoulder to shoulder you know. Sort of unity, solidarity, moment of solidarity that sort of happened and I think those of us who are from these generations are kind of wondering what it will take to have that kind of coming together. To some extent, that was achieved with HIV citizen action response. You can see it happening, not organised because of the way COVID was but are there... is there a global forum, is there a... at the higher levels...sorry I am so muddled. But I was just wondering: can there be another ICPD kind of platform where government is comfortable being

shoulder to shoulder with civil society? There is this symbiotic relationship that is undeniable, I mean that, I don't think that it can completely unravel. Like it is a compact, you know. Is your sense that there are these larger ideas of UHC or of health reform or of Alma Ata even that can help galvanise government and civil societies at sort of the higher policy levels. What is your prognosis around that?

Mirai Chatterjee: Well, I think we have to look for these kinds of opportunities that bring us all together. I can't think of any right now.

Devaki Nambiar: Do we need them?

Mirai Chatterjee: I don't think necessarily we need them. They can be helpful.

Devaki Nambiar: Yes.

Mirai Chatterjee: But given the current atmosphere in our country and even in other countries, I am not really sure whether nationally something like that will happen. Maybe internationally someone will give a push, I am not sure. But, I think, one of the things we were thinking in the Lancet Commission reimagining is that—in fact, I only had suggested to Vikram[121]—, was, instead of having one big bang at national level why not have a whole lot of smaller meetings across the country in places that normally don't get to interact with people like us? Get their views. First of all, they have informed our thinking; they will have informed the report. So, it is our duty to go back and tell them what was in there, what came, test out—is this correct or not, and so on and so forth. Maybe that could be an opportunity to at least bring some of us together. But that will be further down the road.

Devaki Nambiar: But I think given India's diversity and structure, perhaps there is a need to be a big thing at some abstract level. Maybe the whole point is to have that, contextual specificity, that local connection you are saying. I think for some of us, that is what we are sort of counting on.

Mirai Chatterjee: Hoping.

Devaki Nambiar: Yes. Alright. This was the last of my questions... and I wanted to very strictly keep the time, so I don't want to put in any larger questions. But, again Misimi, let's... do you want to talk us through next steps? If you don't have a question.

Misimi Kakoti: I think Devaki one of, you know, the recurring sort of points that the other participants were making was that gender was missing from the community action for health program. What I noted was that Mirai Ben had brought in that sort of component into it and somehow like you know... yes it feels...I mean there was this perspective that was lacking but you [Miraiben] tried to bring that in, and somehow it feels a whole now. What Devaki mentioned about the next steps is that we are going to transcribe this interview and then will be sending you the transcript for your review. And if you would like to edit or add anything else then you can do that, and then we will sort of finalise it and go ahead with publishing it, as part of the other reports we are doing.

Mirai Chatterjee: Sure. Thanks, Misimi. One comment and one question. The comment is that, you know, as Devaki knows, entire SEWA is only women-focused, so in that we have an edge perhaps that whatever we do women are in the lead—by design from day one. Women say, "We don't want men leading our organisation." Of course, we work with the Panchayat and their husbands, and they are all cooperative. But it is very clear that they [women] are in the lead. That's just one comment. And the second comment is: I hope this was useful and not a ramble!

Devaki Nambiar: Not at all, it was not at all a ramble. It was like the history, and the story and the themes from inside of your mind. It was perfect. And thanks, Misimi, for raising that—that you [Miraiben] have laid down the fact that when you talk about community action for health, women are part of the DNA of that. I mean, for me, really, that's sort of where it's at. Even when we think about Jamkhed, Mandwa, Gadhchiroli, all these models... people sit down with the women and ask them... really, there is so much labour and time, and contribution of women, I think it's important to acknowledge that. Thank you for foregrounding that for us.

121 The witness refers to Vikram Patel, a co-chair of the Lancet Citizen's Commission on Reimagining India's Health System.

Mirai Chatterjee: But I think it’s not just about consulting women, allowing women to lead the action. Consulting women is another thing and then men take over the show...

Devaki Nambiar: ...and they design programs like the ASHA program.

Mirai Chatterjee: Exactly...

Devaki Nambiar: Thank you so much for the time.

Mirai Chatterjee: If there’s any gaps, give me a shout—or any after-thought.

Proceeding ends.

In-depth Interview with Dr. Thelma Narayan

Devaki Nambiar: In fact, Misimi, maybe I should handover to you? To just sort of update Dr. Thelma on what's been happening so far. And then you've also, sort of, we had already noted a couple of the points that we specifically wanted you to expand on, which you had mentioned. So, we could start with those or...

Thelma Narayan: Yes.

Devaki Nambiar: Or if you prefer to sort of... I don't know. It's sort of up to you I guess but, Misimi, do you want to give us some steer?

Thelma Narayan: Start with that.

Misimi Kakoti: Okay, thanks Devaki. Ma'am, so—

Thelma Narayan: Just call me Thelma, by the way.

Devaki Nambiar: Okay, thank you.

Misimi Kakoti: There were a couple of, you know, points during the session and also prior to the session, you mentioned that these things require far more expansion than what could be managed during the session. And Devaki and I pulled out those few points while we were transcribing the recording, and one of the first points was that, you know, when you were telling us about the historical origins of community participation in India and monitoring, you had mentioned about the Sokhey Committee, the Bhore Committee, and how these experiences actually had a global influence. And that's where you said, you know, this is very expansive, and we could take it forward in perhaps in a one-on-one session. That's sort of—and the idea of this session is really to give the floor to the witnesses, as in like, what they couldn't perhaps share with us during the sessions, they could continue from there. So, this is one of the points. And then, later on, moving on from there—I think. So maybe, Ma'am, we could start from there. And then later on we could prompt you more.

Thelma Narayan: Sure. From what I was trying to say, with that Sokhey committee report—S-O-K-H-E-Y, Sokhey committee—which was started like pre... in 1939, actually. And then—but was reported later, in fact a little after the Bhore committee. But the Sokhey committee report talked about the need for community-based health workers. One per 1000 [population]. The reason, I think—I mean, this is my reading into it—, it's actually, the report is available online if you Google it. Maybe you've already done it. But it's worth looking at it. You know, it was at the time of the freedom struggle that was going on. You know, I'm just thinking, like you were mentioning the JSA meetings and the MFC and all of that. So, there was an atmosphere among the—I think, this is my imagination—, but among the people who were participating in all of this. That was very passionate, and very much towards, you know... seeing a better India—a better world, you may say.

So, there was... and the reason why I'm a little [passionate]...I don't know if I told you, Devaki, but my father was actually in the INA [Indian National Army] with Subhash Chandra Bose[122].

Misimi Kakoti: Wow!

Thelma Narayan: Yes, very closely linked. And he was commanding one of the 5th Guerrilla Regiment. The sort of energy that came into their, you know, what they were doing...I think... or what they were aspiring for...it was a little bit of imagination, but it was also based on their reality, you know. When the methods that we have today really wasn't available. So there, for them, person-to-person communication was absolutely crucial. And to maintain the hundreds of volunteers that they had—actually, thousands—was only by word of mouth, you know. And by using radio and things like that. So, my sense is that the... I was trying to capture that context... that community participation came in the context of a freedom struggle. The Bandung conference was also in the same context. That was earlier. And I may have mentioned that Nehru[123] participated in the Bandoeng conference, representing India. But it was—and that was the first one [incomplete]—, and interestingly—I may need to check this out, but I think the Bandung conference

122 Subhash Chandra Bose was a key leader during India's struggle for independence from British rule. He helped revive the Indian National Army (INA), which sought "to overthrow British Empire from India". See: https://wbchse.nic.in/html/netaji.html

123 The witness refers to Jawaharlal Nehru, the first Prime Minister of India in office from 1950 to 1964.

was actually supported by the Rockefeller foundation.

Devaki Nambiar: It was.

Thelma Narayan: I mean, there’s another side to that whole thing, but I think there are these dynamics that happen, so all I was trying to say is this is part of a very dynamic context. Community participation should not be seen only in a reductionist point of view. It sometimes, it can be that, in order to achieve our goals—there’s some program goals, or national goals, or global SDGs goals. And therefore, this is a strategic sort of angle. But I don’t think it should be that way. This is much more intrinsic—as a part of life, as a part of living, you know. And so therefore the ethos, or even the ethic of the whole thing, has to be driven differently. So, when we talk of actually participatory decision making, it’s not a mere word, if one actually did it, which the health system right now is not doing. Because it’s always driven by some target. It’s either family planning, even with whatever motive or it’s some you know, TB program. Or now it’s COVID. So, it’s a sort of a quasi-authoritarian—you know, has coercive sort of element to it. That we want to do community engagement or community participation. It’s not necessarily coming from communities in that sense. The ownership may not be, so in that sense there’s still little bit of—I’m just saying—, could be a patronising sort of approach. It’s like, okay, you may have immunisation as one strategy. And on the other hand, you’ll have community participation as another one. But they’ll be the same. The instrumentality, that’s not so… the ethos now [incomplete] I’m just thinking that, after all, who is a frontline person who is catalysing the community processes, you know. What is their positionality in the whole [process]? So, in that sense, it’s often [that] they’re doing what they’re told to do. Now, I know Mitanins in Chhattisgarh, for instance. When we did that Mitanin evaluation[124] back in 2005, it wasn’t as rosy as…

Devaki Nambiar: Yes.

Thelma Narayan: Not at all. So that was really terribly resisted when we gave in the report as you probably heard. There was a lot of angst about it. Therefore, I think now, while everybody is into community participation and engagement—globally, I mean—, …it is a… you know. But I think the spirit of community engagement is what I was trying to get at.

Devaki Nambiar: Yes, yes.

Thelma Narayan: And that’s where I think it fits in more with a community health approach rather than a public health approach. Because public health—and I’m public health—you know… it has that element and if people who are in public health are not aware of that aspect, of a top down—a sort of… whether you want to call it patriarchal, patronising, or whatever. It trickles down the line. So even the relationships between, say, ANMs and the communities, which I’ve see even in my PhD study years ago—I mean 25 years ago—is not that egalitarian at all.

Devaki Nambiar: Right.

Thelma Narayan: So, that was just what I was trying to say. It’s not romanticising the past but learning from it.

Devaki Nambiar: Would you say, Thelma, that there are antecedents where that ethos was achieved? Whether in terms of the articulations at Bandung or the thinking behind Sokhey and Bhore, or even in the smaller experiments that had been underway. Not experiments but just like field efforts that had been underway. And is there a history there that we really must see or be mindful of?

Thelma Narayan: Yes, I think there are lots of, you know, it grew from the NGO movement and many of them are documented. It’s not that you won’t find [it]. But there was a different dynamic. I mean there are umpteen examples—actually in a book that, it’s not a published paper… it’s not a peer reviewed sort of document or book like that. So, you have the Mallur health cooperative[125] where we were working in St. John which is documented. I mean, it is there in the ICMR/ICSSR document of the 1980s. There

124 Read the report of the external evaluation study of the Mitanin programme of Chhattisgarh carried out by SOCHARA here.

125 Mallur Health Cooperative, in Kolar district, Karnataka, linked to St. John’s Medical College, where the existing successfully run dairy milk cooperative was used as a base, and a health cooperative covering four villages was added on. This started in 1973 where locally run and managed health services were linked to elements of the local rural economy, ensuring sustainability (annotation provided by Dr Thelma)

were two documents from ICMR. The Health for All is a different one, that's an ICMR [document]. That's a 'Health For All—An Alternative Approach'.

Devaki Nambiar: Yes, yes. It's not that?

Thelma Narayan: No, it's not that. These are two documents—they are two monographs actually. I'm sorry I made a mistake. It's not ICMR ICSSR. It's just ICMR, it's two ICMR monographs of the 1970s which document a whole range of NGOs, you know and, and it was in a way, it was pre-Alma Ata.

Devaki Nambiar: Right!

Thelma Narayan: Which is also very fascinating. India's experiments with all of this were actually pretty early. It's not linked to Alma Ata alone. In fact, India contributed to Alma Ata, and Alma Ata affirmed what many groups were already doing within the country, you know. So, there's 'Health for a Million' in Kerala. Have you heard of that? It was near what was then called Trivandrum[126]... Thiruvananthapuram. There's a 'Health for one million'[127] project. Then there's an Indo-Dutch project[128] in Hyderabad. Then, there's the Padhar NGO in Madhya Pradesh. And there was one in UP as well. These were all documented. But they were not research studies at all. It was like NGO initiatives where they trained community health workers, and often the reason was there were no other resources available. So that there was a genuine link with communities, you know. I don't know if I answered your question.

Devaki Nambiar: Yes yes, that's exactly [what] I mean. We need to dig up these two monographs. So, we'll set about doing that. I think they might be—

Thelma Narayan: Actually, if you send me an email, Misimi, later, I'll give you the actual reference. But maybe next week, not this week.

Devaki Nambiar: We can definitely do that. So, I think those antecedents and even then—

Thelma Narayan: There was one more report by ISHA—that's the Indian Society of Health Administrators[129]—which brought out a document in the 1990s which has a very nice thing on community participation.

Devaki Nambiar: CHC, of course, has been, you know, doing that—some of the work of documentation also. We talk about that a bit, so we can make sure that it is reflected.

Thelma Narayan: Yes, so, some of this is based on the library that we have. Even these two monographs. Of course, we got to, in fact somebody from SOCHARA—I mean, St. John's, because [unclear audio] was experiments with St. John's—.... We were involved in it as faculty from St. John's. These documents, we have them with the SOCHARA sort of library. It's called CLIC[130], Community Health Library and Information Centre. And the archiving project which Ravi is anchoring. Yes, we've got actually documents from a lot of the work done, including by CHAI, the Catholic Health Association of India[131], which came up with an extremely sort of progressive definition of community health, which is also... no, that was post-Alma Ata. In St. John's, we started training community health workers when we were young faculty, before Alma Ata in 1977. When the Raj Narain initiative[43] was there with the Government of

126 "Thiruvananthapuram, the capital of the state of Kerala was earlier called Trivandrum as was 'rechristened by the English". See: https://trivandrum.nic.in/en/history/

127 Health for One Million (HOM) is a health service initiative of the Diocese of Marthandam in Tamil Nadu, its activities taking place throughout Tamil Nadu's Kanyakumari district. HOM aims to address health needs of poor people under a broader umbrella of "total development of the people." See: http://healthforonemillion.com/history.aspx

128 The Indo-Dutch Project for Child Welfare was pioneered in 1969 within the Chevella Development Block in Hyderabad (now in the state of Telangana). The project's aim was "to provide comprehensive child care within the community including preventive, curative, social, and environmental care for the children of the area and also antenatal services for expectant mothers." The Institute of Child Health, Hyderabad led the health and nutrition component of the project. Source (and read more): Mathur YC, Madhavi V. Village-Level Production of Supplementary Food (Indo-Dutch Project for Child Welfare, Hyderabad). Trop Doct. 1976 Apr 1;6(2):84–6. https://journals.sagepub.com/doi/10.1177/004947557600600216?url_ver=Z39.88-2003&rfr_id=ori:rid:crossref.org&rfr_dat=cr_pub%20%200pubmed

129 The Indian Society of Health Administrators (ISHA) was amalgamated in 1979 with the aim of strengthening the capacity of "health professionals and administrators towards effective management of resources." See: http://www.doccentre.net/Trg/ISHA-Brief.htm

130 Read more about the Community health Library and Information Centre (CLIC) and access its resources here: https://www.sochara.org/clic/CLIC_Resources

131 The Catholic Health Association of India (CHAI) is a network of healthcare and social service institution across India. See: https://www.chai-india.org/

India community health volunteers. You've seen that training manual?

Devaki Nambiar: I haven't seen the manual, no. I've seen documentation of the program.

Thelma Narayan: Oh, well, the manual is very nice. We do have it in our library and so I've never checked online whether it's available. It starts with that Chinese poem. Now that's funny that we have a Government of India major thing starting with a Chinese poem. You know? From a go to the people, live among them, love them.

Devaki Nambiar: Yes, yes.

Thelma Narayan: That one. It has a whole chapter on local health traditions. It's got in detail like this is what a community health worker can do, with all the [incomplete]. It was ahead of its time but, of course, the way it was actually rolled out, as they say, you know, the way the community health volunteers were selected ended up with it being all male. And often, the Gram Panchayat, you know... it didn't actually meet its goals. It's aspirational. Whereas the ASHAs now—there was the Madhya Pradesh evaluation, have you seen that? Of the Jan Swasthya Rakshak[132].

Devaki Nambiar: Yes, that's excellent. I've seen that. But if you mention it, then we'll bring that in. That's great.

Thelma Narayan: Because a big shift came from that evaluation. That's the time when they said, "We'll have all women, not males." Actually, SOCHARA had done the evaluation. And so, Chhattisgarh had all women, the Mitanins. And the ASHAs learnt a lot from Chhattisgarh, I think so. Now, having all women, I think, did alter the community engagement dynamic. Though we pointed out some of the difficulties on the ground, but if you see now, the later work, the Mitanins today are really matured and their relationship is quite different, I would say. I won't romanticise it totally, but it is very different, you know. Just the gender shift made a big [difference]. There are some points in community engagement which I think need to be highlighted, you know. That, the 1970s, were never evaluated formally. The Raj Narain Community Health Volunteers—CHVs, as they call them, Community health volunteers—, so, that was not evaluated. Whereas Jan Swasthya Rakshaks and Mitanins were evaluated. ASHAs were evaluated but I think that [incomplete]. Have you read the ASHA evaluation report[133]?

Devaki Nambiar: Yes, the one that Ritupriya ma'am did?

Thelma Narayan: Actually... Rajani Ved, I think.

Devaki Nambiar: Oh! Rajani and Shwetha. Sorry. Or am I mixing up the AYUSH one which Ritupriya Ma'am led?

Thelma Narayan: I think in this ASHA evaluation, I met some researchers who were part of it, I think the qualitative aspect got a bit subsumed, you know. I think any work that's done on this theme definitely needs to have a qualitative lens. It cannot be done with a quantitative approach to research.

Devaki Nambiar: Yes. No, I totally agree. I mean, we actually looked very closely at your evaluations of JSR[132] as well as Mitanin, when we were trying to look at the scale up of the Mitanin program, and then its evolution into ASHA. Kabir led that, you'll recall, some years after. Yes, and there was just no—we were just thinking, we were so glad we used a Realist methodology, and we were so glad we did that, because there was so much that we had completely not anticipated when we started, you know. And I think even with the ASHA evaluation, it would be interesting to see, perhaps, some of the things that didn't make it into the report. But there has been more and more research on and with ASHAs—that's much more open-ended now. But I think that some of those things, as you were saying, have gotten—some of those dynamics have gotten locked in in some parts of India, right? In terms of, you know, is—are they being instructed to do X or Y, or how are those dynamics playing out? How, for instance, does it work with the other

132 The Jan Swasthya Rakshak scheme was initiated by the Madhya Pradesh government in 1995 as part of the Integrated Rural Development Programme (IRDP) through which unemployed rural youths were enrolled to provide curative, preventive and promotive health services in all the villages of Madhya Pradesh. Read the review report of the scheme by SOCHARA here. http://sochara.org/uploads/aboutuploads/wEGUFQ_21.The JSR scheme of MP 1997 (1).pdf See: https://www.sochara.org/what_we_do/Community_Health_Workers

133 The evaluation of the ASHA programme was carried out by the National Health Systems Resource Centre (led by Dr. Rajani Ved). Read the report here.

forms of community action or designed forms of community action, like VHSNCs, you know, Mahila Arogya Samitis, and you know? Are we able to roll this out at scale [emphasis], if you will. Or is it inherently something that's meant to be very local, very contextual and—so, I think, yes.

Thelma Narayan: There, I think, when institutionalisation is to some extent necessary, but it can also be problematic, you know. I think the scope for, say, self-reflexivity, I mean, I don't think anybody would actually do that... I can't imagine it being done in a formal program; I mean, in the government program. Of spending that amount of time with ASHAs and others, VHSNC members to really have that sort of an in-depth discussion. But I think, as we go forward, that should be something that needs to be done because it then speaks to the [incomplete] I really hope your work influences... and I'm sure it will... this Lancet Citizens' Commission[134].

Devaki Nambiar: Yes, I'm working with Sapna and Arnab on the other piece.

Thelma Narayan: The district case studies?

Devaki Nambiar: Yes, so we're doing that, we are planning actually that fieldwork out at present. And I had mentioned to Vikram a few times that we're doing the Witness Seminar. So, Arnab and Sapna in any case will be looking through what we have...and we'll share it. Though I think we may need to create some sort of shorter document or a summary of some of the points for the commission, so you have more to work with. I think it will be harder for you all to just have transcripts. So yes, we'll definitely be able to do that...

Thelma Narayan: One of the things, I don't know whether this would be even possible, but they are thinking of these public webinars. You may have seen the one on AYUSH. So, I don't know whether your team will be interested in doing like a public webinar.

Devaki Nambiar: Oh absolutely. I think that would be great. I was a discussant—on the one that Barbara and Sumit [audio unclear] did based on their study and it was great interaction. This would be... but again, you know Thelma, I feel like, you know maybe Misimi and I can put some of the points together. But the whole point of this is to have the witnesses speak. So perhaps we would have you speak and then show some of the summary results and then some of the other witnesses. I don't know. We would have to think about what is a meaningful way to put this out to the public, that honours the method. We'll have to think about that a little bit. And just see, yes, but that's a great idea.

Thelma Narayan: No because it's also one way. See ultimately, the Lancet Citizen's Commission is also trying to be research based, and Witness Seminars is sort of relatively new in India. I've been in one or two—the one CEHAT was doing on TB or something recently. But it would also be a way of showcasing the methodology itself being discussed. Also, the topic, you know.

Devaki Nambiar: And we've got some things—I know there are a fair number of timelines—but we've added—made one—another one. And I think, there are some thematic areas that are coming out. And then some of these debates in fact—Misimi was reminding me earlier, even with respect to the seminar that we had earlier—you had talked a little bit about this idea, about this notion of countervailing power. And that on the one hand, with respect to the direction NRHM was going in, and the steer which was given. But on the other hand, some of the ideological fractures within the movement and how those were negotiated or even reconciled with or perhaps even not—and how those functions. All of these are part of the grand narrative of reform. Right? I think it's important particularly in a citizens' commission, right? to raise some of these nuances and the granularity of what has happened and what continues to happen in India. I don't know how to present that though. And I don't know if you want to as part of this seminar—talk a little bit about these two ideas. One of countervailing power and what did that mean, what did that look like. And then second, some of that journey how some of these ideological differences and debates came up and how they were—how

134 The witness refers to the Lancet Citizen's Commission on 'Reimagining India's health system'. See: https://www.thelancet.com/pdfs/journals/lancet/PIIS0140-6736(20)32174-7.pdf. See note 118.

they sort of—progressed.

Thelma Narayan: You know, one of the reasons... in my thesis which is again in the mid 1990s, I really grew—did a lot of my own work in this voluntary sector space. St. John’s is also in a way voluntary sector, but we left and then SOCHARA, CHC[135] has been [incomplete] It was a thought that came based on reading etc. That what was the difference that was happening as a result of the work of all these NGOs across the country, and I was focusing my work using TB as a case study. And then I thought that actually we were not a countervailing power. The voluntary sector. I mean that was one of the... in a way a hypothesis. And, we were doing it, it was very helpful to the communities where these initiatives took place. No doubt in those communities there was a change. Even Tuberculosis in certain areas, there was a decline due to the work of the [incomplete] Because once when I asked in NTI why this particular district had a lower TB prevalence, they said it’s because CMC Vellore’s there and the way they do their program, you know. They had a community-based program. They were just saying very nonchalantly. So, I was quite taken aback by that. I thought it was a positive thing.

If you think of the whole country—which we have to—I mean, as any Chairman or HM and even now, with the Lancet Citizens’ Commission, we have to think of the entire population. Then we were not a countervailing power, and the policies were going in a different direction. Privatisation had just, sort of started in the 80s in a bigger way, and so therefore that formulation [audio unclear] not countervailing power. Rather that there is a need for a countervailing power. And JSA in its own way came up as a result. JSA was beyond MFC, MFC was more like a thought current, as we say. Some of us felt we can’t only be a thought current, interesting though it is. There has to be something further. Something beyond that, you know. So MFC played a really important role, but JSA had a much stronger participation. Now it’s not, like, a political... it’s not that sort of participation, I don’t think people are going to vote for JSA. But it had its own dynamic and it played a role, a significant role, for instance in getting ‘communitization’ in the National Rural Health Mission. Where did that come from, why did the Government of India suddenly think of ‘communitization’? You think the same bureaucrats suddenly thought this through. I mean they think they do. But I don’t think so, I think it’s in that dynamic between civil society and very good and progressive civil servants, you may say that. IAS officers and others. And it had political traction, some degree. I wouldn’t say great. Because if you think of it, the governments of the day never ever spoke about the NRHM or the ASHAs. They never acknowledged. I always think it is rather strange. You know, because it had made a difference. So, I think there was a little bit of countervailing power at that time. But my take is that—I’m not speaking as a researcher now but as a [incomplete]—that countervailing power has got a bit stagnant. And we need to—and as you say—the failures and whatever differences that took place—and bound to take place. I mean there’s nothing unusual about it. But, I think sometimes, even when talking of community participation, one has to keep the larger picture in mind and that’s the tough call. Because to keep the larger picture of, whether it’s privatisation which is now like ongoing in a very aggressive way. How does one expect a community health worker to actually debate and discuss that? It’s not that it’s impossible. But how many initiatives are actually giving time towards unravelling that.

I think only [audio unclear] had the capacity of, you know. I remember, Anant Bhan had written, they used to tell the health workers about say pharmaceutical pricing. But it’s the rare sort of thing—it’s not the run of the mill thing. But a lot of the Hesperian Foundation[136] documents actually... they do talk about some of these issues, through the medium of popular education. So, now why shouldn’t citizens, or community actually know those. As much as one knows about the COVID,

135 The Community Health Cell (CHC) “is a functional unit of Society for Community Health Awareness, Research and Action (SOCHARA)”, collaborating with non-governmental and governmental organizations, campaign groups, and people’s movements “to make them part of this ‘Health for All’ movement.” See: https://www.sochara.org/clusters/Community_Health_Cell_CHC_Bengaluru

136 Read about the Hisperian Foundation here: https://hesperian.org/

you know, this Coronavirus – those [policies] are as dangerous as the virus.

Devaki Nambiar: Yes!

Thelma Narayan: Those policies or that policy trend, is as dangerous as a virus. That's in my [view] I mean others may have different views. They may think it's an answer to the problem but, I don't know if I'm getting a bit [incomplete].

Devaki Nambiar: No, I mean I think there's something about the—the approach by which to deal with all of these—whether it is a virus—or whether it is the encounter in the health system or what one is being offered. So, I don't think it is per se endorsement of one or the other. But, what are all the considerations or how does one approach it? When you have a frontline health worker who has a set of targets that she's already responsible for and so on. There's, you know, there's already a lot to put on that person and then anticipate some of these other things. Though that said, so many of them have anticipated a lot of—for example in NCDs, they've been dealing with for many very years. Because those are the concerns of their communities. They've been figuring out ways to respond to those needs. But…yes, to have that approach I suppose—is hard to anticipate. And even align around, like what do we all decide is the common curriculum for this part. This broader part—not just the skills, and the competencies—but the approach or something. So that's much harder, I think. Though as you said some folks like Amitha, Dr Anant and all have been doing it. So, yes.

Thelma Narayan: But none of this is at a scale. But it's seen as being more dangerous you know. And then we break down to a lack of trust in the health system. Which is another sort of very important area nowadays. But then in order for there to be trust in the system, the system has to be trustworthy.

Devaki Nambiar: Yes.

Thelma Narayan: And that has to be experienced as being trustworthy. So, I don't know whether that is happening because I think if one asks communities… do they really trust, even the private sector, which they are sometimes forced to use. There's a lot of doubts. So that's why I think research really does help to uncover some of these things. And it has to be a constant endeavour. I think for community participation—I mean why would a community want to participate? We are talking from a health sector point of view, aren't we?

Devaki Nambiar: Yes.

Thelma Narayan: Why would they want to participate? Only if they felt—one is the ownership, but also the trust, that they will be listened to, that they are equal partners in this. Is that really happening, do you think? I don't know, Devaki, you're closer to the ground than I am.

Devaki Nambiar: Well, it's so variable, right. So, a lot of my work is in Kerala, where things look a certain way. I think for many subgroups even in that state—if you've done some research in Wayanad on the tribal ASHA 'Oorumithram' project[137]. There's a lot of effort being put into selection and things like that of those ASHAs. But there's still not that trustworthiness of the system per say. You know, there are still gaps. There are still ways in which the community feels let down or unconcerned—like it's not as relevant to their lives. And some of that, as you've been saying, and I heard you, Dr. Ravi talk about this for many years. It has to do with the disease orientation of the health system, right? So then—there—you don't consider the health system in the context of health. You don't see it connected to your traditions, to your practices of self-care. You are not… that's not what it is. So then that connection with the "hard to reach" or whatever populations that we have decided to call that—that connection isn't there, let alone with those. It's not even really there in urban centres, right. So, that continuity and that sort of [incomplete]

Thelma Narayan: So, there's this huge cultural disconnect. I mean, I have no bones about talking about cultures—it has nothing to do with the

137 "Oorumithram" is Kerala's community health worker program, also called the Hamlet ASHA program, focussed on the areas of the state with tribal communities. See: https://gh.bmj.com/content/6/Suppl_5/e006261

political dynamic of the day. It's just that, 'us' and 'them' will always be there, sort of alienation. Because as you said, the self-care—traditions or experiences are different you know. They're not non-existent. I mean, they could be—I mean someday, science could help in the process. But it has to be done in a sensitive manner—so that I'm not sure it happens. But what you're saying about Kerala is very interesting, I mean it's...

Devaki Nambiar: Yes, so I mean even—I mean there anyone can, can't but be impressed by the sort of attention to detail, the use of not just... I mean and I would say research could be used more; but a lot of experiential and tacit knowledge of implementers is used. And I think—yes, that is valued, and it is used. But then, there are legacy factors for how things are the way they are in Kerala—and then you know, so. It's very hard even in India then to contend with just that variation of scale, of effort, of attention to detail from place to place—even within a district. Even, you know, let alone sort of the—the variation across the country. And I think that's where even what you're saying about the...this you know, acting as a countervailing force. The prevailing force in each place looks sort of different. So being a coherent countervailing force and even applying oneself at the national level with the awareness that there's so much sub-national variation—it becomes hard for a movement to reckon with the fact that there is that variation. That's a very important part of what community action is about. And then trying to cohere at the national level, you know what I mean? So when you're trying to make that case for the variation—at the same time—so it's—I think it's even like in terms of an argument it's kind of so complicated. And then you have to deal with how policy making works and what are the contingencies and idiosyncrasies of how that works and you know. So ... I mean, in some of the interactions we've had, or at least I've managed to have, with some of the national level civil servants—they've said—just do it at the subnational level—it's really, this is not—this is not the field of play, you know. This is okay—this can be sort of generic, but the real field of play is much more local. It's district, it's Taluk, it's state even [incomplete]

Thelma Narayan: That's true. By the way have I told you about the district health assembly[138] going on presently in Tamil Nadu?

Devaki Nambiar: No. So please add that.

Thelma Narayan: Oh, yes, yes! You must! I mean, then you'll really be up to speed because this is an initiative that the [incomplete]. I mean there's a new government regime now in Tamil Nadu. And though now finally this is coming from the health system reform project[139] which is supported by the World Bank. This idea was mooted a few years ago. I forget exactly when but maybe two to three years ago. And it didn't take off. So, during the—I think they may have had like four district health assemblies which were virtual and which none of us know about and it's not... I mean we haven't seen any documentation.

But now—I'm part of that planning. I mean, as part of the AGCA, I do some of the Tamil Nadu things. I used to when Rakhal, Amir, and all of them were [incomplete], so now it's really gained steam and they were planning 10 district health assemblies in the month of January, starting end of December. Four have already been held. Our team went for the first one in Tiruvannamalai. I actually have a draft report of that which our team has written. I can share that with you. Because it'll be like an unpublished document.

Devaki Nambiar: Fantastic. What is the structure of these? How have they...? So, the genesis is sort of that work and I think it was—

Thelma Narayan: I mean, interestingly, the objective of the whole thing is to listen to citizen voice. Which is really something, you know. And they've got this state level committee, but they have district level organising committees. Each district is slightly differently done, which is excellent. The spirit of the whole thing is there. And it was so well organised.

138 The Government of Tamil Nadu convenes district and state health assemblies (meetings) "to increase citizen's voice and develop participatory public policy" by involving the community, civil society, academia and other partners in collective decision making and planning in health. Read more about the health assemblies in the state here: https://tnhsp.org/tnhsrp/images/files/go/GO.157-Health-Assembly.pdf

139 The Tamil Nadu Health System Reform Program (TNHSRP) is a project implemented by the state's Department of Health and Family Welfare (DoH&FW) and supported by the World Bank. It aims at improving the quality of care, non-communicable disease and reproductive and child care, focussing primarily on the SDG 3 targets. See: https://tnhsp.org/tnhsrp/

Because I joined online—they had that option. But it was an in-person meeting, with about maybe 150 people or so. And the first one in Tiruvannamalai—they had every block. It was Tiruvannamalai and one more district, which was the next neighbouring [district]. They've been creating some new districts in Tamil Nadu, recently. So it was like two districts and 18 blocks. All 18 were represented and some of them had virtual representation, via Zoom. You could see different rooms with like 15 people sitting there, block this that, and you know. It was quite amazing. And they had done mock drills before this. Village Panchayat, they've had meetings. But that's where maybe a little bit of guided democracy is what I would call it. It is that they were all raising issues about the infrastructural needs. Lots of very practical issues, you know, that were needed from people's point of view. And, then there was one in Ooty which was really amazing. In fact, all of them are amazing, to tell you the truth. The 4 that have been held and I think one or two they didn't put on that recording option on Zoom.

Devaki Nambiar: Oh my god, yes! Dr Sundar told me about the one in Ooty on the 31st. I was in Coonoor, so he was like, just go. I was like, I have small children. I didn't have childcare, so I didn't go. But you are right, this is what that was.

Thelma Narayan: It was a fantastic one.

Devaki Nambiar: You're saying they didn't put on the recording—the recording option for all of them?

Thelma Narayan: <laughs> No, except, I think, the last one. But there are going to be three or four, there are going to be a few more. I don't know if any of you would be interested in going for it—would you?

Devaki Nambiar: I could find out. I know that at least Dr. Sundar had told me about—he had only told me about this one. I didn't know there was a whole set. But one can of course find out. I hope they're doing documentation and…

Thelma Narayan: I don't know how they're doing that [documentation]. So here actually we had Karthik from our team who went on a really short notice. And he did a nice report and the others who went added to it. We've got some pictures and all of that. But there may be some more that are coming and Dr. Sangeeta, who is a Deputy Director there, she'd asked me if… now, my mother is 98, so I can't actually travel. She's not doing too well in the past two months, so it's difficult for me to go. As you know, childcare, it's the same thing, but reverse. So, I'll let you know if there are others that are happening.

Devaki Nambiar: Please! I think we will and, actually, for the citizens' commission Tamil Nadu, of course, is one of the states that's chosen. So, we will be trying to do some documentation. But if there are some of these kinds of things happening, you know in vivo or whatever, then we should sort of start to document that.

Thelma Narayan: It's all in Tamil. So, you'll understand, I think, na?

Devaki Nambiar: I can follow Tamil, but I won't be able to transliterate and do all of that. I don't speak Tamil. I'll be able to follow. But we have a number of colleagues who would be quite comfortable and would be able to communicate and document. We are also, I think, for the Citizens' Commission[118], we were planning to reach out to Murali sir, and to connect with his… and then we may have some, team members and—

Thelma Narayan: You can go, na?

Devaki Nambiar: Anyway, so.. But yes, if that's ongoing this month, that would be—I mean it sort of brings us to a very—

Thelma Narayan: Here and now sort of thing.

Devaki Nambiar: Yes, and a sort of hopeful note. It remains to be seen but I mean I think just creating these kinds of spaces and however they emerge, keeps—[background sound] sorry. Keep some sort of momentum around this and to some extent I was also thinking Thelma if it can also be done in slightly different ways in different places. You know? So it's almost not that same formula where people are wondering, "how are we supposed to do this," but rather saying "how do we want to do this?" You know? I think, inviting that kind of approach.

Thelma Narayan: So here, there’ve been like people with disability who are bringing up their issues, there are citizen’s voices. There was one group was all into like, having herbal gardens in health facilities. Very interesting ideas. And there was some citizens’ forum in Ooty which has raised huge amounts of money because Ooty being sort of a tourist-y place also, maybe I don’t know. It was something different about Ooty. That’s the thing.

Devaki Nambiar: There is something different about Ooty and the Nilgiris.

Thelma Narayan: <laughs> So, they got a lot of money, and they redid the whole district hospital. It was quite amazing to listen to the ways—and that’s where citizen contribution has helped, you know. Right to getting the emergency room, and the OT and everything. I mean real detail. But very good quality. And it wasn’t done… this was certainly not in any patronising way. I think these are amazing case studies. So, I think in India, given—I am very optimistic about citizen participation, because I think you can’t subdue the energy of Indians—of the citizens, you know? Whatever the larger macro picture…

Devaki Nambiar: Oh, how nice to hear that. Yes. And yes, I think there are little pieces here that we should—that we should follow up on. We’ve sort of tumbled our whole hour away…And I just realised—I don’t—but I think a lot of what we had wanted to kind of bring in, and actually a lot of this, to be honest, Thelma, will be in the annotation. I think there’s a lot of documentation that has to now be brought in. So I think we’ll do a transcription of this, share it with you, and request you on some of the annotations. I know some of these are actually manually in the SOCHARA archives. So we’ll just make reference to that. And then in cases where it is sort of digitised—or available online or something like that—we’ll make reference to that. But I’ll just loop back to Misimi in case we’ve sort of jumped over any of the pieces you were thinking we should expand on.

Misimi Kakoti: Devaki, I think we have sort of covered the main two key points that we had decided. And I feel that this is how you imagined, how a Witness Seminar was supposed to be, right? Like very candid conversations. Also, I feel that, I mean, Thelma has really like closed the conversation into—like it was a very interesting loop because she had started with the freedom struggle movement and then closed with the saying that you know, that we all have very high hopes from, you know, the energy of citizens participation in the country. And I think that’s a—I mean, for me at least, that’s a very important key takeaway.

Devaki Nambiar: Yes. And I daresay, especially for people in our position it’s nice to end on an optimistic note—we end up having very sort of—wistful conversation with people saying “anyway, that was then.” You know? And we are like. That’s okay. That’s what’s going to happen now. But there are some great things happening now. I think, so, thank you. Thank you for that.

Proceeding ends.

HEAL

Health equity action Lab

The George Institute
for Global Health

@GeorgeInstIN
www.georgeinstitute.org.in
@GeorgeInstIN
