# Supplementary material for: How communitization begets and endures sarkarikaran: a witnessed history of community action for health in India’s national rural health mission
Source: BMC Health Serv Res. 2025 Jul 8;25:940. doi: 10.1186/s12913-025-13058-0 (PMC12235939; doi:10.1186/s12913-025-13058-0)
Supplement: Supplementary file 3 — Supplementary Material 3. [file 12913_2025_13058_MOESM3_ESM.docx]

**(Second) Witness Seminar on Community Participation in Health in India:**

**Community Based Accountability Mechanisms under the**

**National (Rural) Health Mission**

**Guiding Questions**

*Note: Witnesses may choose across these categories of questions and present their reflections as they deem appropriate*

*A. Emergence*

1. What was the role of community action and voice in the genesis of NRHM in 2005?
2. How was the “communitization” – more specifically, the community based accountability framework – brought into NRHM? Who were the key players? Key institutions?
3. How was the initial design of and the institutional support mechanisms for community action in health evolved (e.g. Community Based Monitoring Process (CBMP) pilots, Advisory Group on Community Action (AGCA) and others, Village Health, Sanitation and Nutrition Committees (VHSNC), Hospital Management Committees/Rogi Kalyan Samitis (RKS), Mahila Arogya Samitis (MAS))?

*B. Evolution and Institutionalisation*

1. What were the experiences from implementing the pilot and scaling it up across states (considering different contexts of states)? Were goals achieved? What were the challenges? Lessons learned? Key variations in approach across states?
2. How were community-accountability processes received/carried forward by state implementers? What were notable experiences/episodes?
3. With regard to perspectives of communities, what were key experiences/episodes in the years after the basic model was rolled out? What about civil society organisations? Other community formations?

*C. Evaluation and Impact*

1. What were the key successes and failures of the CBMP process under NRHM? How is the success measured and defined? What implications did the integration of NUHM with NRHM into NHM have on ‘communitisation’?
2. Has power sharing and participation among the stakeholders (civil society/government/community) been impacted due to these shifts? How?
3. What is the legacy of community action for health in NRHM? What are the lessons and insights for India in the current phase of Pradhan Mantri Atmanirbhar Swasth Bharat Yojana (PMASBY)? For the world?
